# Lessons Learned in Orbitrap MS-Based Isotope Ratio Analysis of Organic Acid Mixtures

**DOI:** 10.1021/acs.analchem.5c07111

**Published:** 2026-03-21

**Authors:** Hugo G. Machado, Elliott P. Mueller, Júlio C. O. Ribeiro, Giovanni B. Bevilaqua, Gabriel F. dos Santos, Alexandre A. Ferreira, Ygor S. Rocha, Surjyendu Bhattacharjee, John M. Eiler, Boniek Gontijo

**Affiliations:** † Chemistry Institute, 67824 Federal University of Goiás, Goiánia, Goiás 74690-900, Brazil; ‡ Division of Geological and Planetary Sciences, 6469 California Institute of Technology, Pasadena, California 91125, United States; § Department of Geological Sciences, 1877 University of Colorado Boulder, Boulder, Colorado 80205, United States; ∥ Division of Geochemistry, PETROBRAS Research and Development Center (CENPES), PETROBRAS, Rua Horácio Macedo, Ilha do Fundão, Rio de Janeiro, RJ 21941-915, Brazil

## Abstract

Stable isotope analysis is a vital tool across chemistry, geology, and environmental science, but conventional Isotope Ratio Mass Spectrometry (IRMS) techniques have limited capabilities for site-specific or multiply substituted (“clumped”) isotope analyses, and are particularly limited for analyses of complex mixtures without prior analyte purification. This study addresses this gap by employing a high-resolution Orbitrap mass spectrometer to directly measure the ^13^C/^12^C ratio in a model naphthenic acid (1,2,3,4-tetrahydro-2-naphthoic acid, THN) within complex organic matrices. We applied a “zero-enrichment” experimental design to evaluate accuracy and precision by comparing pure standards to the same compound in synthetic samples resembling natural waters. Complementary experiments using low-molecular-weight organic acids and natural rumen fluid were conducted to define the method’s limits under controlled and severe ion-suppression conditions. The results demonstrated that matrix effects and ion statistics can substantially degrade both accuracy and precision under certain conditions. At very low analyte concentrations, incomplete ion accumulation led to heightened δ^13^C variability, a condition analogous to a “blank effect”. Paradoxically, adding 1% NH_4_OH improved the precision of ^13^C/^12^C measurements (reducing the relative standard error from ∼0.80‰ to ∼0.63‰ at 0.1 μM THN), despite a reduced signal, by promoting more stable deprotonation and minimizing ion suppression. We also identified that coaccumulated ions, even when baseline-resolved, such as a matrix-derived fragment at *m*/*z* 177, degrade precision by perturbing the space-charge balance. Removing this interference fully restored precision, underscoring the need to control coaccumulating ions. Crucially, experiments with small organic acids demonstrated that moderate ion suppression does not lead to isotopic bias, which emerges only when severe suppression reduces analyte ion counts below a critical statistical threshold. Finally, we identified an “isotopic stability plateau”—an optimal signal range where δ^13^C measurements are most precise and accurate, poised between noise-dominated and space-charge-distorted regimes. This work demonstrates that Orbitrap-MS can perform reliable isotope analysis in complex organic mixtures when instrumental and chemical parameters are carefully optimized, opening new applications in petroleum geochemistry, environmental forensics, and other topics.

## Graphical abstract

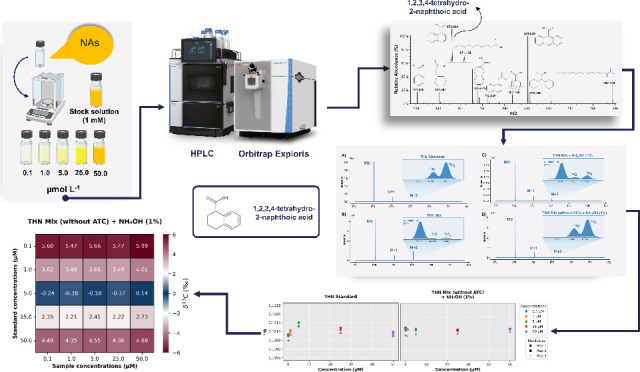

## Introduction

Stable isotope analysis is a powerful approach for investigating chemical, environmental, and geological processes at the molecular level. Monitoring natural variations in isotopic composition allows researchers to unravel reaction mechanisms,^1,2^ reconstruct paleoenvironmental and climatic histories,^3,4^ and ensure forensic traceability in areas such as food production, forensics, and petroleum geochemistry.^5–8^ In the oil industry, for instance, isotopic signatures are increasingly used to track the origin, transformation, and migration of hydrocarbons, as well as to assess biodegradation and water–oil interactions within reservoirs.^9,10^ These diverse applications rely on the accurate measurement of isotope ratios, which is typically performed using mass spectrometry.

Isotope Ratio Mass Spectrometer (IRMS) remains the gold standard for isotopic quantification in a wide range of samples. The greatest strengths of IRMS are its combination of excellent sensitivity and reproducibility. However, application of this technique to most compounds requires the complete conversion of analytes into simple gases—such as H_2_, CO_2_, N_2_, SO_2_, and O_2_—which are then introduced into the mass spectrometer and ionized, typically using electron ionization (EI).^11^ While highly effective for molecule- or material-average isotope analysis, this approach generally destroys intramolecular-level information such as site preferences or nonstatistical multiple substitutions, as atomic sites in analytes are generally transformed into relatively simple compounds that do not retain elements of original molecular structure^12–15^ (specialized techniques of GC-pyrolysis-GC-combustion-IRMS are exceptions to this rule for some compounds^16^). These limitations are particularly critical when studying complex environmental or geochemical samples, where the identity and distribution of specific molecular isotopologues can carry invaluable information. And they are particularly acute in cases where analytes are not easily purified, such as when chromatographic peaks coelute in chromatograms of complex organic mixtures.

To overcome the limitations of IRMS and enable the analysis of distinct molecular isotopologues, new analytical tools are needed.^17^ The Orbitrap mass spectrometer (Orbitrap-MS) has emerged as a powerful tool for isotope ratio analysis on intact molecules. This instrument combines high resolving power and high mass accuracy with diverse ionization techniques, including both Electron impact and softer electrospray ionization (ESI), enabling the analysis of molecular isotopologues without their prior conversion into simple gases.^15^ Thus, Orbitrap-MS offers a complementary approach to traditional IRMS, expanding the scope of isotopic applications across fields such as environmental science, biochemistry, and petroleum geochemistry.

Orbitrap-MS has shown significant potential for high-precision isotope ratio analysis across a variety of compound classes.^17,18^ Its key advantages are its extraordinarily high mass resolving power (up to 100× that of even specialized high resolution IRMS platforms), high sensitivity, and integration into platforms that enable several modes of ionization, online GC or HPLC, and MS/MS experimental designs. The high mass resolving power enables the resolution of most easily imagined isobaric interferences up to masses of 100s of u, while its high sensitivity allows for the detection of low abundance isotopologues and study of subnmol sample sizes. Furthermore, its compatibility with soft ionization techniques, such as ESI, preserves the molecular integrity of target compounds.^12,15,17,19^ These capabilities have spurred the development of methods for diverse analytes, including oxyanions and amino acids.^20–23^ For instance, Neubauer et al. (2020)^15^ employed ESI-Orbitrap-MS to analyze oxyanions such as nitrate, sulfate, and phosphate, measuring δ^34^S, δ^18^O, and δ^15^N values with precisions of 1–2‰ relative to international reference standards. Similarly, Mueller et al. (2022)^12^ reported the simultaneous analysis of δ^2^H and δ^13^C in acetate, achieving precisions better than 3 ‰ and 0.5 ‰, respectively. These results highlight the method’s robustness for isotope analysis of polar compounds, paving the way for its application to increasingly complex sample types.

Despite recent advances, the application of Orbitrap-MS in isotopic studies remains largely focused on method development using reference standards. While approximately a dozen studies have begun to transition toward applied research,^12,15,17,21,24–32^ the literature still predominantly addresses inorganic species, relatively simple polar matrices, or relies on extensive off-line purification. Notable examples of this transition include the work of Hilkert et al. (2021),^30^ who developed a nitrate-based method for environmental samples, and Mueller et al. (2024),^32^ who measured acetate in subsurface brines. Even when studies extend the analysis to more complex organic matrices— as demonstrated by dos Santos et al. (2025)^29^ in natural lipid characterization—there is often an acknowledgment that further research is required to refine methodologies and evaluate potential matrix effects in truly challenging sample types. A critical gap thus remains in the application of Orbitrap-MS to complex organic mixtures, where analytical artifacts still hamper the accurate measurement of intact molecular isotopologues.

The complex organic matrix of waters produced from petroleum-field wells represents a significant yet untapped frontier for isotope-ratio Orbitrap-MS. This water contains a diverse array of polar organic molecules, most notably naphthenic acids (NAs), which result from the oxidation and microbial degradation of petroleum hydrocarbons.^33,34^ NAs are relevant to two important issues: they are geochemical tracers for reservoir processes such as biodegradation and migration, and they pose a significant environmental threat due to their acute and chronic toxicity to aquatic life, with adverse effects reported at concentrations exceeding 2 mg/L.^35,36^ The isotopic composition of intact NAs (including δ^13^C, δ^18^O, and perhaps, δD, site-specific, and multiply substituted properties) could provide insights into both the origin and fate of this class of organic compounds. However, direct isotopic analysis of these molecules in such complex mixtures requires a validated Orbitrap-MS methodology. Overcoming this analytical barrier is essential for advancing geochemical models and performing more accurate environmental risk assessments in offshore oil production.

Here, we present and validate a method for the direct isotopic analysis of naphthenic acids by ESI-Orbitrap-MS, including consideration of complex mixtures. We selected 1,2,3,4-tetrahydro-2-naphthoic acid (THN), a representative naphthenic acid commonly identified in produced water,^37,38^ as our model compound. Our approach demonstrates, for the first time, accurate isotopologue measurements of an intact organic acid in a mixture without requiring chemical derivatization, chromatographic separation, or off-line purification. By establishing the analytical workflow and figures of merit for THN, this work expands high-resolution Orbitrap isotope analysis to real-world samples and complex mixtures of polar compounds, offering new possibilities for petroleum geochemistry and environmental forensics.

In addition to validating the workflow on a representative NAs, complementary experiments were performed using a simple organic acid system (acetate, propionate, and butyrate). This additional validation confirmed that Orbitrap-based isotope ratio analysis provides robust and accurate results across both complex petroleum-derived matrices and small organic acid mixtures, strengthening the generality and reliability of the methodology.

## Materials and Methods

### Chemicals and Materials

LC-MS grade methanol (MeOH) was purchased from Bio Scie Company (Anápolis, Brazil). The benzoic acid isotopic reference standard was purchased from the International Atomic Energy Agency (Vienna, Austria). Benzoic acid, 9-anthracenecarboxylic acid (ATC), 1-naphthoic acid, 1-naphthaleneacetic acid, cyclohexylacetic acid, 1,2,3,4-tetrahydro-2-naphthoic acid, 2-methyloctadecanoic acid, decanoic acid, dicyclohexylacetic acid, and 3,5-dimethyladamantane-1-carboxylic acid standards were obtained from Sigma-Aldrich (St. Louis, U.S.A.). All chemicals, their respective exact masses, and structures are shown in Table S1.

### HPLC and MS Instrumentation

Analysis was conducted on a Vanquish Neo UHPLC system (Thermo Fisher Scientific, United States) coupled to an Orbitrap Exploris 240 Mass Spectrometer (Thermo Fisher Scientific, United States) equipped with an OptaMax NG electrospray ionization (ESI) source (Thermo Fisher Scientific, United States). The entire system was controlled by Xcalibur software (Thermo Fisher Scientific, United States). For the small organic acid measurements, a Vanquish HPLC was coupled to a ESI HF Q-Exactive Orbitrap MS. For each injection, 100 μL of sample was pulled though the sample loop and then injected into the 5 μL/min flow of LC-MS grade methanol (Fisher Chemical, Optima), which carried the sample directly into the Orbitrap MS. The total run time was 10 min, with the first and final 2 min intervals culled from data analysis to introduce and flush out the analyte, respectively. Thus, only the interval between 2 and 8 min was used to calculate the isotope ratios.

The ESI source was operated in negative-ion mode with a spray voltage of 2.8 kV, a capillary temperature of 280 °C, and an S-lens RF level of 70%. Sheath, auxiliary, and sweep gas flow rates were set to 0, 1, and 0 (arbitrary units), respectively. The mass spectrometer operated in Full Scan mode over the *m*/*z* range 173–178, targeting the deprotonated molecule ([M-H]^−^). Data were acquired at a resolution of 60,000 (at *m*/*z* 200), with an automatic gain control (AGC) target of 10^6^, a maximum injection time of 100 ms, and averaging two microscans per scan.

For the smaller organic acid measurements, the ESI source was operated in negative ion mode with a spray voltage of 3.0 kV, a capillary temperature of 320 °C, and an S-lens RF level of 60%. Sheath, auxiliary (100 °C, and sweep gas flow rates were set to 10, 3, and 1 (arbitrary units), respectively. The mass spectrometer operated in Full Scan mode over a scan range of *m*/*z* 57–62 (acetate [M-H]^−^), 72–75 (propionate [M-H]^−^), and 85–90 (butyrate [M-H]^−^). Data were acquired at a resolution of 60,000 (at *m*/*z* 200) for acetate and propionate, and 120,000 (at *m*/*z* 10 to the sixth), with a maximum injection time of 100 ms and one microscan.

### Experimental Design: Simulated Zero-Enrichment via Matrix-Modified THN Solutions

The experimental design was based on a ‘zero-enrichment’ approach, which is a bracketing analysis using the same compound as both sample and standard. This strategy enables the direct assessment of analytical accuracy—since the expected isotope delta value (δ^13^C) is theoretically zero (O ‰)—and precision, which is determined from the standard deviation across the replicate δ^13^C measurements.^19,39^

To investigate the influence of chemical matrix effects on isotope ratio analysis, a more complex experimental design was implemented. Simulated sample matrices were prepared containing the target analyte, THN, combined with a mixture of the other acids listed in Table S1. All solutions were prepared from a single stock of the THN standard, providing a common isotopic reference to assess its behavior under different chemical environments.

Four sets of solutions were prepared, in which the concentration of each coexisting acid was fixed at 10 μmol L^–1^, while the THN concentration was varied across five levels (0.1, 1.0, 5.0, 25.0, and 50.0 μmol L^–1^):

(1) Pure THN: the THN standard in solvent alone, serving as the reference.
(2) MIX: THN combined with the mixture of all other acids from Table S1.
(3) MIX + NH_4_OH: Solution (2) fortified with 1% (v/v) ammonium hydroxide to enhance deprotonation.
(4) MIX -ATC + NH_4_OH: Solution (3) prepared without 9-antracenecarboxylic acid (ATC) to evaluate its specific role as a potential isobaric interference.

The analytical sequence was designed to evaluate matrix-induced shifts in the δ^13^C values of THN. In this case, virtual bracketing pairs were created by comparing matrix-containing solutions with the pure THN standard at each tested concentration level. The injections were performed in blocks, starting from the lowest THN concentration (0.1 μmol L^–1^) and progressing to the highest (50.0 μmol L^–1^). Within each concentration level, the injection order of the four solution types (Pure THN, MIX, MIX + NH_4_OH, and MIX-ATC + NH_4_OH) was randomized to minimize systematic bias. This entire analytical sequence was repeated five times to yield five independent replicate measurements for each condition. A detailed injection sequence is provided in the Supporting Information (Table S3).

### Expansion of Experimental Design: Low-Molecular-Weight Acids and Biological Matrix Validation

A complementary set of experiments using low-molecular-weight organic acids was conducted to test the method’s generality. Multiple mixtures of acetic, propionic, and butyric acids were prepared, holding the acetate concentration constant (50.0 μmol L^–1^) while varying the relative proportions of propionate and butyrate. Each mixture was then analyzed against a pure acetate reference, allowing direct assessment of any δ^13^C or δ^2^H bias caused by coeluting acids.

Additionally, a natural rumen fluid sample was analyzed to evaluate isotope ratio behavior in a complex biological matrix containing the same short-chain organic acids. This test provided a real-world validation of the controlled experiments by examining whether suppression effects observed under laboratory conditions could be reproduced in an authentic biological environment. The rumen fluid sample enabled assessment of acetate δ^13^C stability across a range of butyrate:acetate ratios, including scenarios with extreme ion suppression.

Together, these complementary tests served as a benchmark alongside the THN matrix experiments, demonstrating that while moderate ion suppression does not compromise isotopic accuracy, severe suppression in real matrices can lead to measurable deviations.

### HPLC-ESI-Orbitrap MS

The NAs MIX samples were analyzed by direct infusion. A sample volume of 50 μL was injected and delivered to the ion source at a constant flow rate of 5 μL min^–1^. The total run per sample was 15 min, comprising a 10 min data acquisition window followed by a 5 min methanol system flush. For data processing, the ion current signal corresponding to the 2–8 min time segment of the acquisition was extracted to exclude the initial solvent front and the final wash period.

For acetate-propionate-butyrate mixtures, instrument settings are in Table S4, with the Orbitrap operating in negative-ion mode and the mass range adjusted to *m*/*z* ∼ 57–62 to target the acetate [M-H]^−^ ion (*m*/*z* 59) and its ^13^C isotopologue.

### Data Processing

All raw data processing was performed using *IsotoPy*, a custom software developed in Python3 by our research group at the Federal University of Goiás (available at http://www.isotopy.com.br, see additional information in the Supporting Information (SI)). To enable native processing of the vendor-specific files, *IsotoPy* incorporates the RawFileReader library (C#/.NET), provided by Thermo Fisher Scientific via http://www.github.com/thermofisherlsms/RawFileReader.

The quantification framework of the software is based on the ion counting model established in Thermo Fisher’s IsoX software, as described in a recent work by Kantnerová et al. (2024),^40^ which defines the theoretical ion count for an isotopologue as

1 $$\mathrm{ion count}=\frac{S}{N}\times 3\times \sqrt{\frac{R_{n}}{r}}\times \sqrt{\mu }$$

where *S* is the signal intensity of the peak, *N* is the corresponding baseline noise, *r* is the mass resolution of the measurement, and μ is the number of microscans. $\sqrt{R_{n}/r}$ corrects for changes in ion statistics relative to a reference resolution (*R*_*n*_) of 240,000,^40^ at which the empirical constant 3 was determined.^17,41^ For the small organic acid measurements, a constant of 4.4 was used following Eiler et al. (2017).^17^ However, since this constant is removed in the calculation of the isotope ratio, this discrepancy does not affect our results.

The isotope ratio (^13^R) is then calculated as the quotient of ion counts for the heavy (^13^C) and light (^12^C) isotopologues. As both signals are acquired under identical experimental conditions, and using constant parameters (i.e., resolution, microscans, and instrumental settings) are identical for both isotopologues, thus instrumental biases associated with these parameters cancel out. This simplifies the expression to depend solely on the relative signal-to-noise values as outlined in eq 2:

2 R13=ion countsC13ion countsC12=SC13/NC13SC12/NC12

Beyond this core calculation, *IsotoPy* automates the entire analytical workflow, including scan filtering, statistical analysis, outlier rejection, and the final calculation of δ^13^C values relative to certified standards. This high-throughput, automated process was essential for ensuring the reproducibility of the measurements reported herein. Further technical details are provided in the Supporting Information.

### Calculation of δ-Values

The isotope delta (δ) value expresses the relative difference in isotopic composition between a sample and a reference material. Equation 3 defines its calculation as

$$\delta_{\mathrm{sample}/\mathrm{STD}}=[\left(R_{\mathrm{sample}}/R_{\mathrm{STD}}\right)-1]\times 1000$$ 3

where *R* denotes the isotopic ratio of heavy to light isotope (e.g., ^13^C/^12^C), sample refers to the analyte of interest, and STD to the reference. The result is reported in parts per mile (‰).^12,30^

To correct for instrumental drift over time, δ^13^C values were calculated using a standardization strategy inspired by the approach proposed by Csernica et al. (2023).^19^ The acquisition sequence consisted of seven blocks acquired alternately between standards (odd-numbered blocks) and samples (even-numbered blocks). Instead of using simple bracketing,^30^ a linear regression was constructed using the average isotope ratios of the four standard blocks (1, 3, 5, and 7). The resulting model was then used to predict the expected isotope ratios at the sample blocks (2, 4, and 6). These predicted reference values were then compared to the measured sample ratios using eq 3 to compute δ^13^C. The final δ^13^C value reported corresponds to the average of the three individual δ^13^C values derived from this model-based correction, and the standard deviation among these values is referred to as the reproducibility error. This approach enables time-resolved normalization, effectively correcting for instrumental drift and minimizing systematic error. A detailed description of each virtual bracketed analysis is provided in the Supporting Information.

## Results and Discussion

Reliable isotope ratio analysis by Orbitrap mass spectrometry demands not only high instrumental precision but also a meticulously controlled ionization environment. In complex organic matrices, such as the naphthenic acid and low-molecular-weight organic acids mixtures investigated here, phenomena such as ion suppression, isobaric interferences, and space-charge effects can introduce systematic biases and increase variability, compromising both the accuracy and the precision of the analysis. Therefore, a central challenge is to identify conditions under which an analyte’s measured isotope ratio remains consistent (i.e., unbiased and reproducible), regardless of matrix composition. Achieving this state, which we term “isotopic stability plateau” is fundamental for ensuring data reliability. This section presents a systematic approach to identify such stable conditions, using 1,2,3,4-tetrahydro-2-naphthoic acid (THN) as the model analyte in NAs MIX, and acetate in low-molecular-weight organic acids mixtures.

### Ionization Behavior of Naphthenic Acids: Role of p*K*_a_ and Structure

The ionization behavior of the acid mixture was first evaluated by full-scan ESI-Orbitrap-MS analysis in negative ion mode (Figure 1). All ten target naphthenic acids were successfully detected, with the THN peak appearing well resolved and intense. The observed intensities followed a trend consistent with known p*K*_a_ values: acids with lower p*K*_a_, such as 9-anthracenecarboxylic acid (p*K*_a_ = 3.65), produced stronger signals, while weaker acids showed reduced ionization. Additionally, molecular structure played a secondary role: aromatic acids typically yielded more stable signals than aliphatic or branched species. THN, with an intermediate p*K*_a_ of 4.57 and aromatic structure, demonstrated both consistent detection and moderate response. This performance makes THN a suitable representative probe for assessing matrix-induced effects.

**Figure 1:**
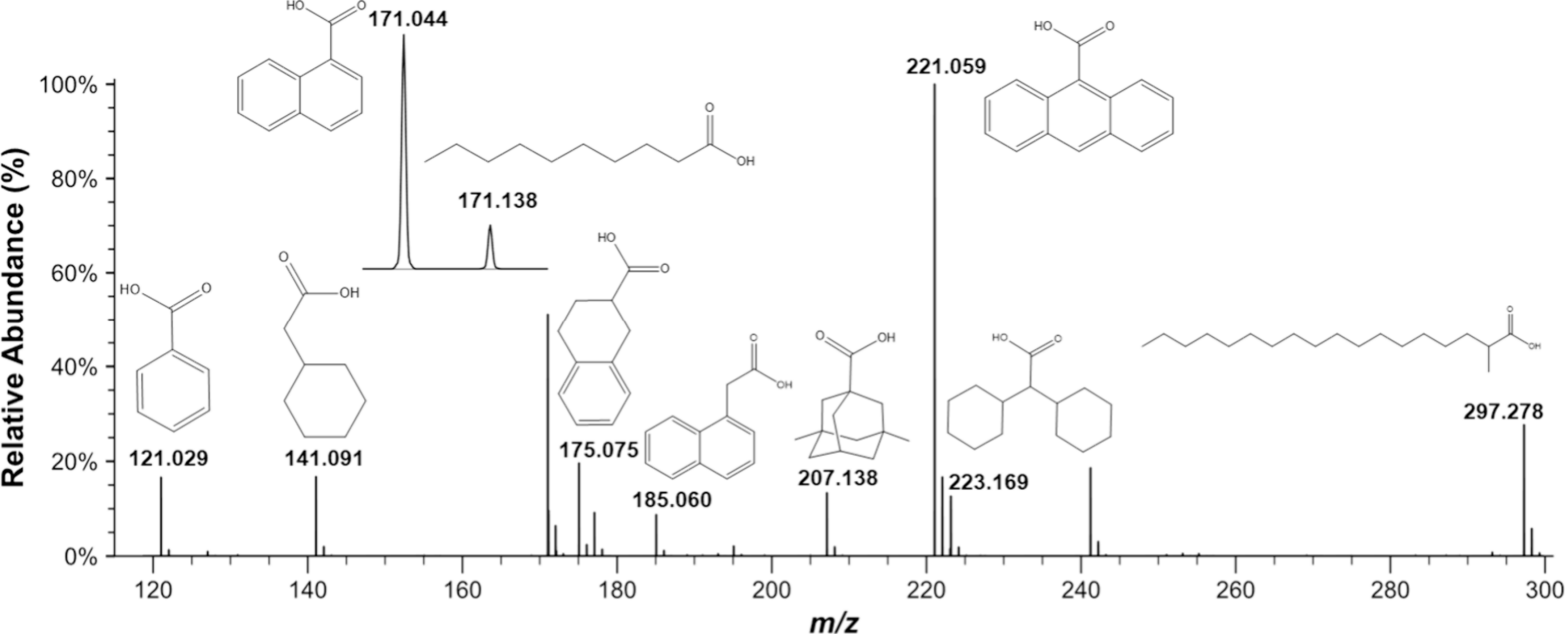
Negative-ion mode ESI-Orbitrap mass spectrum of the acid mixture (structures shown in neutral form; peaks correspond to deprotonated [M-H]^−^ species.

Unlike the naphthenic acid mixtures, the low-molecular-weight organic acids shared similar p*K*_a_ values (4.75, 4.88, 4.82). However, their ionization efficiencies differed significantly. These differences were driven instead by molecular structure, where the extended hydrophilic side chains of propionate and butyrate made them more amenable to ESI than acetate. However, acetate was still quantifiable in organic acid mixtures.

As a preliminary step, the isotope profile of THN was analyzed to confirm that its principal isotopologues could be baseline-resolved. Figure 2 displays the high-resolution mass spectrum, where the monoisotopic peak [M_0_ – H]^−^ at *m*/*z* 175.076 is clearly distinguished from the singly ^13^C isotopologue M + 1 at *m*/*z* 176.079. The measured mass difference of 1.003 Da is in excellent agreement with the theoretical mass difference between the two isotopologues, confirming the peak assignments. This result demonstrates that the instrument provides sufficient resolution to distinguish the two most abundant isotopologues of THN, validating its use as a model compound in subsequent matrix effects studies.

**Figure 2:**
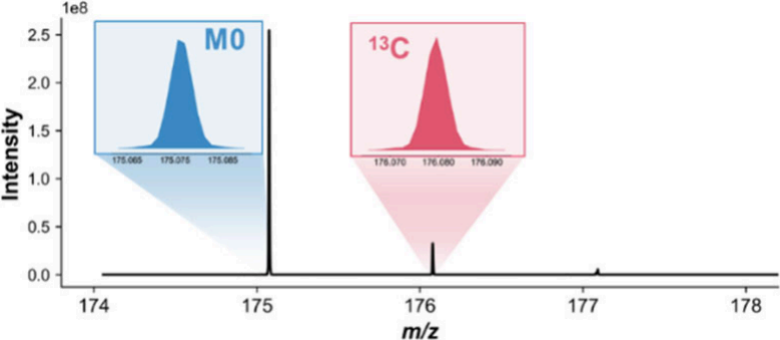
High-resolution ESI-Orbitrap mass spectrum of THN isotopologues: monoisotopic M0 at *m*/*z* 175.076 and single ^13^C M+1 at *m*/*z* 176.079.

As a further validation, the isotope profile of acetate was also examined within the acetate-propionate-butyrate mixtures. The monoisotopic and ^13^C isotopologue peaks of acetate are baseline-resolved under the applied conditions and did not deviate from the *m*/*z* ratios found in pure standards of acetate, confirming that the Orbitrap resolution was sufficient to distinguish the principal isotopologues of this smaller acid system as well. Representative high-resolution spectra and corresponding total ion chromatograms (TICs) are provided in the Supporting Information (Figure S4).

### Ion Statistics and Isotopic Precision

Injection time (IT) is a key diagnostic parameter in Orbitrap analysis, as it reflects how long the instrument needs to accumulate enough ions to satisfy the AGC target, which specifies the number of charged ions to be accumulated in the C trap and then injected into the Orbitrap. Injection time, therefore, provides a sensitive, indirect indicator of overall ionization efficiency under a given set of conditions.

In the pure THN Standard, IT exhibited the expected inverse relationship with concentration, decreasing from ∼32 ms at 0.1 μM to ∼0.8 ms at 50 μM. When the coeluting acid matrix was introduced, ion suppression became evident: IT values increased across the concentration range, particularly at 0.1 μM, where THN-MIX injections frequently approached the upper time limit of 100 ms (Figure 3). This behavior indicates that, under matrix-rich conditions, ionization efficiency decreases substantially, forcing the instrument to extend accumulation time to reach the AGC target.

**Figure 3:**
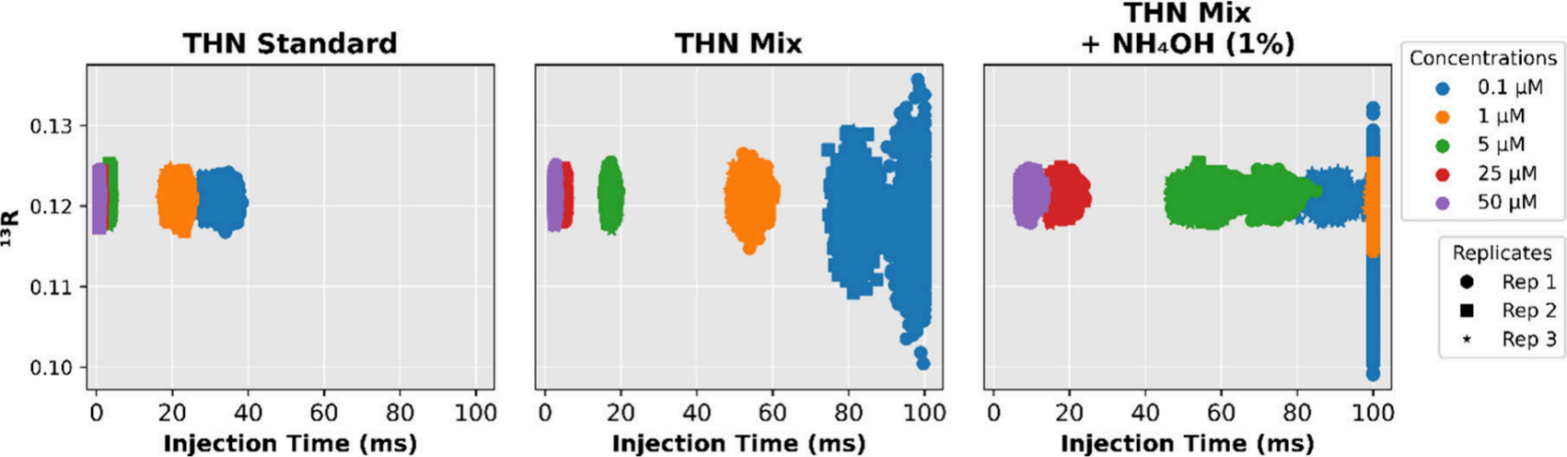
Relationship between injection time and isotopic ratio (^13^C/^12^C) across all systems and concentrations. Each concentration is shown in a different color; replicate injections are distinguished by marker symbols.

This effect directly impacted isotopic precision. As shown in Table S2, the acquisition error (relative standard error of ^13^R across scans) was markedly higher for the THN-MIX at low concentrations—0.80‰ at 0.1 μM compared to 0.17‰ for the standard. To assess whether this imprecision was purely statistical, the observed acquisition error was compared with the theoretical shot-noise limit.^12,15,17,19^ For the pure THN standard, the measured error remained near the shot-noise limit (ratio ≈ 1.06) across all concentrations, whereas in the matrix-containing solution, the ratio exceeded 1.35 at 1 μM, confirming that ion population insufficiency dominated the noise behavior. This pattern is characteristic of the “blank effect”, where low ion counts and elevated background noise amplify isotope ratio variability.^41^

Adding 1% ammonium hydroxide introduced a distinct behavior. Although the selected ion current (SIC) decreased significantly — e.g., from 3.4 × 10^8^ to 1.1 × 10^8^ at 50 μM (Figure 4) of THN-MIX solutions — the isotopic precision paradoxically improved. At 0.1 μM, the acquisition error decreased from 0.80‰ in THN-MIX to 0.63‰ in THN-MIX + NH_4_OH. This apparent behavior arises because the alkaline environment modifies both the chemical ionization regime and the instrumental ion-accumulation regime.

**Figure 4:**
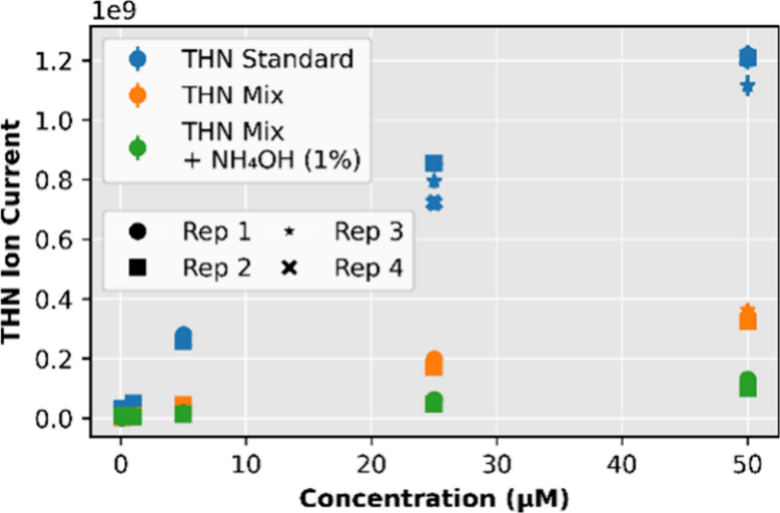
Mean THN Ion Current (SIC) as a function of concentration for each sample type.

From a chemical standpoint, under basic conditions, all carboxylic acids in the mixture, including THN (p*K*_a_ ≈ 4.6), are deprotonated, reducing ionization competition and stabilizing droplet formation.^42^ The presence of ammonium ions, however, increases solution conductivity and introduces charge competition that can lower total signal intensity.^43^ Despite the reduced absolute signal, the ionization process becomes more uniform and less prone to transient fluctuations^44^ — an effect that explains the improved reproducibility observed for most THN-MIX + NH_4_OH replicates.

Instrumentally, however, this same condition brings the system closer to the upper the AGC control boundary at low analyte concentrations. To evaluate this behavior, we examined the product TIC × IT (Figure S6), which approximates the total ion load delivered to the C-trap and provides a direct diagnostic of AGC regulation. For both THN standard and THN-MIX, TIC × IT values remained close to the AGC target (∼1.1 × 10^6^, in arbitrary units) across all concentrations, indicating stable AGC-controlled accumulation. In contrast, for THN-MIX + NH_4_OH, all three replicates at 1 μM and the first two at 0.1 μM fell below this target, indicating that these injections approached or reached the 100 ms IT limit. The deviation was most pronounced for replicate 1 at 0.1 μM (2.07 × 10^5^) and replicate 1 at 1 μM (4.94 × 10^5^). Only replicate 3 at 0.1 μM remained within the AGC-controlled regime, maintaining TIC × IT values near the target and injection times below 100 ms (see Figure 3 and Figure S6). This replicate also exhibited isotopic precision comparable to the analyses at higher concentrations, reinforcing the conclusion that deviations from AGC control drive the observed loss of precision at low signal levels.

When the instrument operates outside the AGC control regime, the number of ions reaching the Orbitrap depends directly on instantaneous fluctuations in total ion current (TIC), leading to increased scan-to-scan variability in ion population and, consequently, in isotope-ratio measurements. This explains why, in the THN-MIX + NH_4_OH data set, most replicates exhibited improved precision due to chemical stabilization, whereas those that exceeded the IT ceiling showed degraded reproducibility. In other words, the interplay between chemical and instrumental effects defines a dual regime: alkaline conditions enhance the statistical stability of ion generation, but only as long as AGC control remains effective.

### Effects of Mixed Organic Acid Matrices on Acetate Isotopic Ratios

Complementary experiments were conducted to evaluate how coexisting short-chain organic acids influence acetate isotope measurements. In Figure 5, each panel illustrates the results of varying the acetate fraction in mixed solutions:

**Figure 5:**
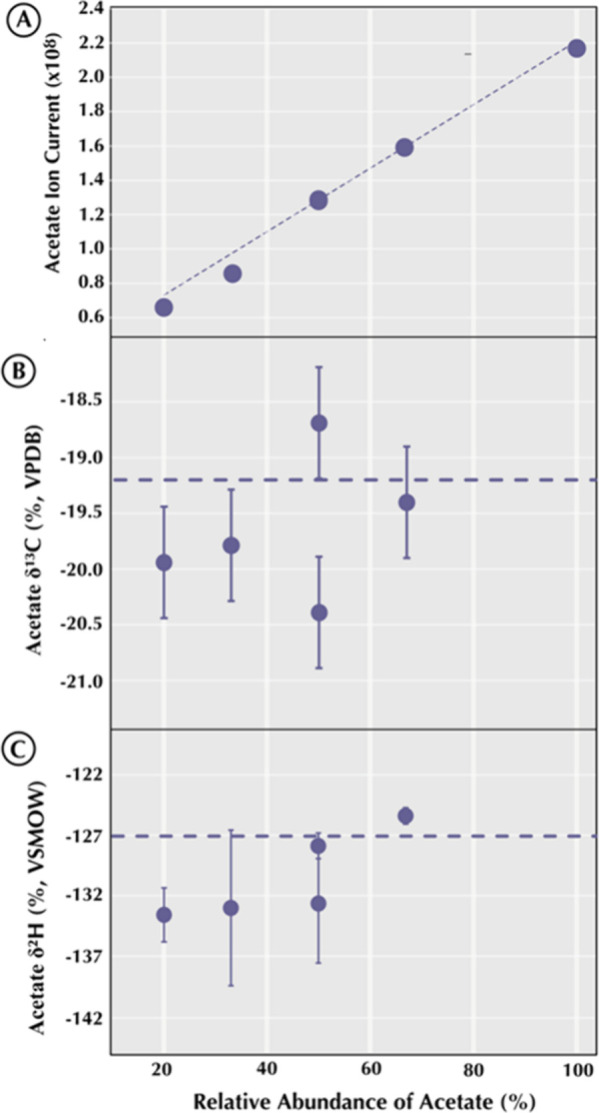
Five mixtures of acetate, propionate, and butyrate at varying proportions (holding acetate constant at 50 μM), with known δ^13^C and δ^2^H values, were measured against a pure acetate standard: (A) acetate ion current (SIC) vs acetate fraction; (B) acetate δ^13^C vs acetate fraction; (C) acetate δ^2^H vs acetate fraction.

As shown in Figure 5, as acetate’s relative abundance decreases, its ion current drops nearly linearly. In fact, the SIC can be up to three times lower than in the pure-acetate standard, demonstrating strong competition for ionization (acetate “loses” intensity to the other acids). The measured δ^13^C of acetate remains constant (within analytical error) across all mixtures. There is no systematic offset or trend. Similarly, the acetate δ^2^H values show no significant dependence on mixture composition. These results demonstrate that although adding other carboxylic acids greatly suppresses acetate’s signal (Figure 5A), it does not induce measurable isotope fractionation (Figure 5B,C). In other words, moderate ion-suppression alone is insufficient to bias isotope ratios.

Only under conditions of extreme suppression do the isotope values begin to deviate, as confirmed in the rumem fluid experiments. In this real matrix, where acetate was present together with substantial amounts of butyrate and propionate, the δ^13^C values of all three acids clustered within a narrow, accurate range under most conditions. However, when the ratio of butyrate and acetate currents exceeded approximately 5:1, acetate’s ion signal was driven down to critically low levels, and its δ^13^C values diverged sharply from the expected composition (Supporting Information, ).

This finding demonstrates that the isotopic stability observed in controlled mixtures also extends to complex biological matrices, and that bias only emerges when suppression is so severe that the analyte-specific ion population falls below the statistical threshold for robust isotope ratio determination.

### Matrix Ion Interference: The Impact of Coaccumulated Ions

During the evaluation of THN at intermediate concentrations (5 μM), where ion populations are sufficient to suppress “blank effects”, we observed a consistent and unexpected signal at *m*/*z* 177.07 within the narrow acquisition window used for THN quantification. This feature appeared in both the THN-MIX and THN-MIX + NH_4_OH solutions but was absent in the THN standard, suggesting a matrix-derived origin. Based on its mass and consistent presence across replicates, the signal was tentatively assigned to 9H-anthracen-9-ylium (see Figure S2), a fragment originating from the ATC acid, the most acidic compound in the mixture. To test this hypothesis, we prepared a modified version of the matrix excluding ATC (THN-MIX without ATC + NH_4_OH). In this new condition, the *m*/*z* 177.071 signal was no longer detected, confirming that the ion was indeed linked to the presence of ATC in the solution (Figure 6).

**Figure 6:**
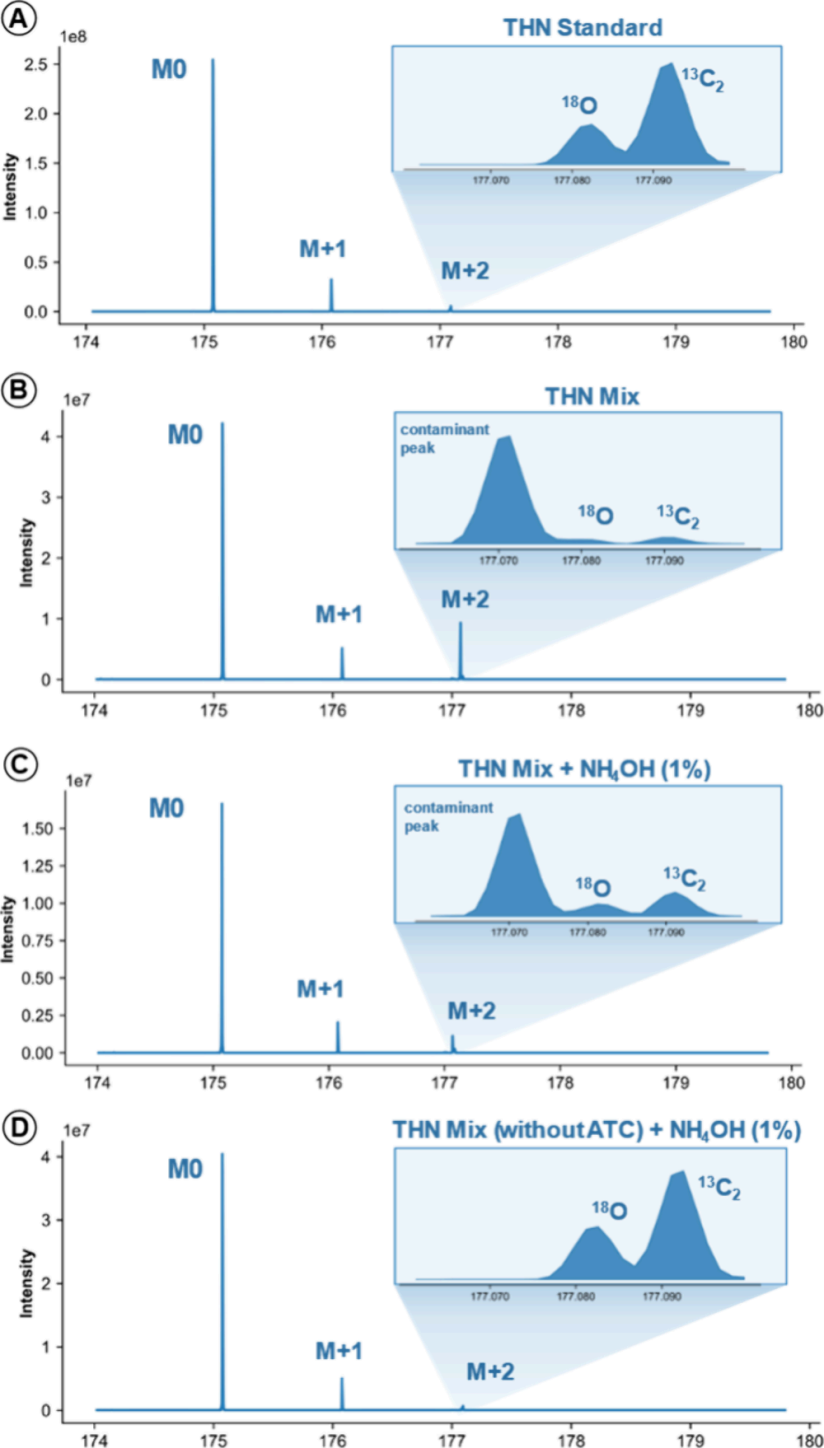
Extracted mass spectra of the 174–180 *m*/*z* region for four experimental conditions at 5 μM: (A) THN Standard, (B) THN MIX, (C) THN MIX + NH_4_OH (1%), and (D) THN MIX without ATC + NH_4_OH (1%). The insets highlight the region around *m*/*z* 177, showing the expected isotopologues of THN (e.g., ^18^O and ^13^C_2_). In spectra B and C (both containing ATC), a distinct contaminant peak at *m*/*z* 177.07 is clearly observed.

Although this contaminant does not coelute with THN and is fully resolved by *m*/*z*, its persistence within the ion accumulation window likely has measurable consequences for the analytical outcome.^14^ Kantnerová et al. (2024)^40^ and Hofmann et al. (2020)^14^ emphasized that even fully resolved ions, when present during ion accumulation, can perturb ion dynamics and induce space-charge effects in the Orbitrap.

In our experiments, such an influence is most evident when comparing the THN-MIX to the modified matrix without ATC. At 5 μM, a concentration where blank effects are no longer a dominant factor, the presence of the *m*/*z* 177.07 peak in the MIX correlates with increased variability in both injection time and isotopic ratio (^13^R). Upon removal of ATC, the 177.071 signal vanishes, and the overall analytical stability markedly improves, as evidenced in Figure 7 (see Figure S1 and Table S2 for more details). These observations suggest that the ATC-derived fragment, while not directly interfering with the mass peaks of interest, may be disrupting the accumulation or injection efficiency during ion processing (likely through subtle space-charge interactions or competitive ion focusing effects).

**Figure 7:**
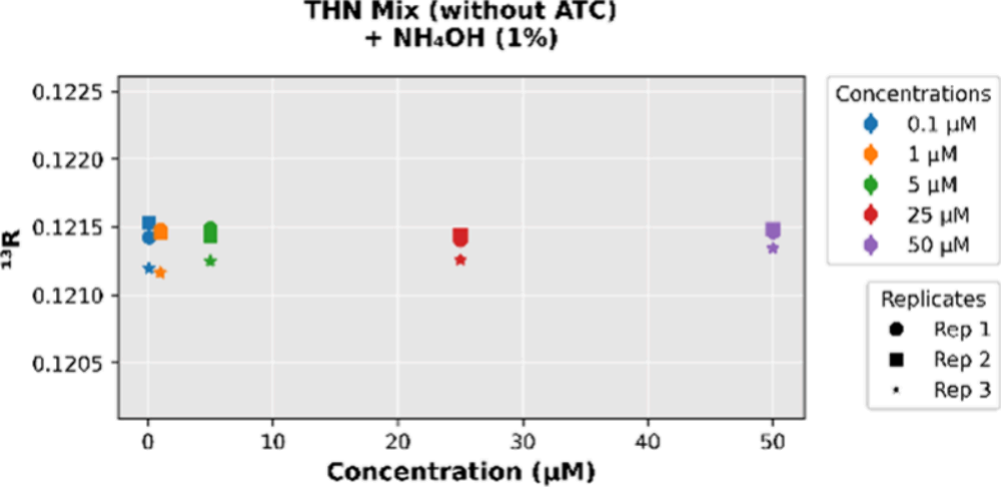
Stability of the isotope ratio (^13^R) across concentrations for the THN MIX (without ATC) + NH_4_OH (1%) system.

These observations align with complementary acetate experiments (Figure 8), in which a carbonate ion contaminant at *m*/*z* 59.98—falling within the quadrupole window of the acetate isotopologues—produced a systematic suppression of δ^13^C by up to 25% when the carbonate ion’s relative abundance exceeded ∼10%. Furthermore, the measured δ^13^C of acetate had an inverse correlation with the abundance of the carbonate ion, both across injection blocks (Figure 8B) and on a scan-by-scan basis (Figure 8C). Despite being baseline-resolved, the contaminant perturbed Orbitrap ion dynamics and reduced isotopic accuracy. Together with our THN observations, these results demonstrate that systematic isotope shifts occur only when contaminants fall within the selected mass window and coaccumulate with the analyte ion population.^14^

**Figure 8:**
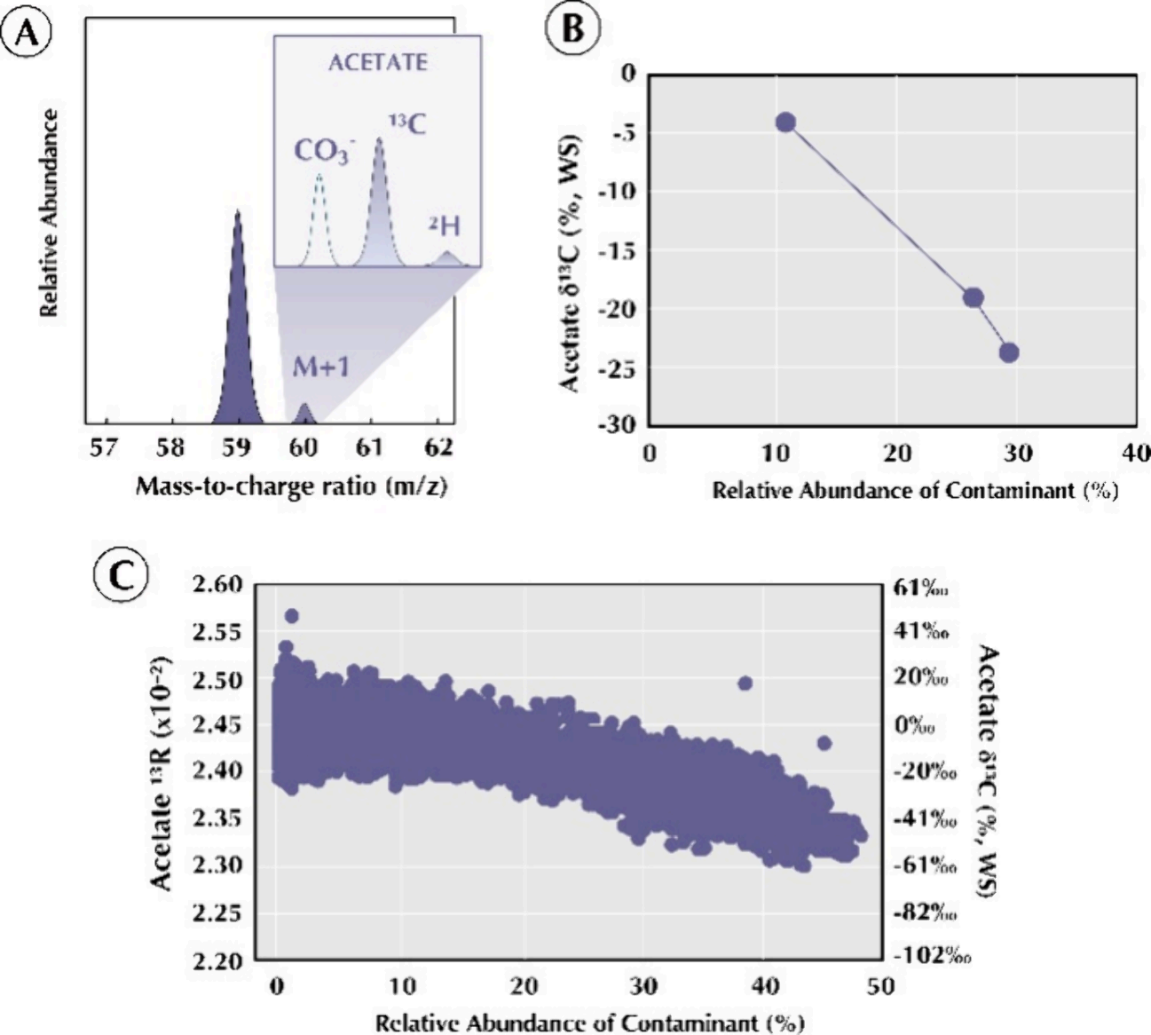
Carbon isotope ratio of acetate is suppressed by mass-resolved contaminants, including the carbonate anion (A). This leads to (B) progressive increases in the abundance of the carbonate ion contaminant across three acquisition results in lower ^13^R measured. (C) Collated scans across the three acquisitions highlight this correlation further, demonstrating that it does not have consistent isotope ratios unless the carbonate ion is <10% of the SIC value. This trend was not observed for hydrogen isotope ratios.

These results demonstrate a critical principle for high-accuracy analysis: the localized ion dynamics within the instrument can be more detrimental to isotope accuracy than generalized, bulk matrix effects. It is essential to distinguish between these two phenomena. Bulk matrix effects primarily arise during the electrospray process (ESI source), where high concentrations of coexisting species lead to ionization competition. While this “bulk” suppression reduces the absolute analyte signal and increases statistical noise, our results show it does not inherently bias the isotope ratio if ion counts remain sufficient.

In contrast, localized space-charge perturbations occur postionization, specifically within the mass-selective ion optics, the C-trap, and the Orbitrap analyzer. In isotope ratio workflows, the quadrupole is typically set to a narrow mass window, allowing only the analyte and nearby coaccumulated ions to reach the C-trap. Within the C-trap, these ions compete for a share of the fixed AGC target (e.g., 10^6^ charges). Mass-resolved contaminants within this window, such as the ATC fragment at *m*/*z* 177.07 or the carbonate anion at *m*/*z* 59.98, can perturb ion accumulation dynamics and introduce scan-to-scan variability in the analyte population. Finally, inside the Orbitrap analyzer, the close physical proximity of these co-oscillating ion packets exacerbates localized space-charge effects. This can distort ion trajectories and frequencies, leading to systematic shifts in measured ^13^C/^12^C ratios despite baseline resolution.

The improvement observed upon removing these contaminants strongly supports this conclusion. In the case of the MIX, eliminating ATC simplified the ion population, yielding more stable injection dynamics and mitigating localized space-charge effects between peaks. For the acetate mixtures, carbonate elimination was achieved by adjusting electrospray conditions: lowering the spray voltage (<3.2 kV) and introducing a mild sweep gas flow reduced the carbonate signal to <0.1% of the total ion current, effectively restoring isotopic accuracy. Together, these results highlight that precise and reliable isotope ratio measurements depend not only on managing total ion current, but also on controlling the composition and behavior of the ions admitted into the analyzer.^14^

### The Isotopic Stability Plateau: A Unifying Principle for Trap-Based MS

Interestingly, even under idealized conditions (i.e., in the absence of matrix components or known contaminants), the THN standard showed noticeable variation in its measured ^13^C/^12^C ratio (^13^R) across the concentration range. At low THN concentrations, the measured ^13^R values were highly scattered due to limited ion counting statistics and background noise, indicating poor precision in this regime. In contrast, at higher concentrations, a clear systematic decline in the measured ^13^R was observed. For example, as THN concentration increased from 5 μM to 50 μM, the ^13^R dropped from approximately 0.1214 to 0.1208 – a relative decrease of about 5‰ (as depicted in Figure 9). This reproducible downward shift at high intensity signals a systematic bias, suggesting that, beyond the “blank-effect” of low-count variability, instrumental factors influence the accuracy of isotope ratio measurements even in the absence of extraneous matrix interferences.

**Figure 9:**
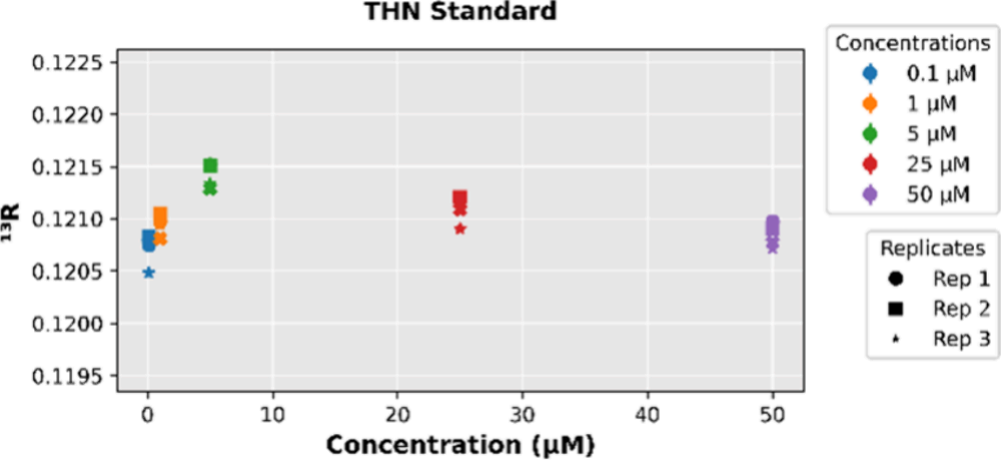
Variation in the isotope ratio (^13^R) across concentrations for the THN Standard, illustrating a nonmonotonic shift in ^13^R.

One plausible explanation for the high-concentration bias is the inherent nonlinearity of the Orbitrap at elevated signal intensities. It is well established that trap-based mass analyzers like Orbitrap can exhibit slight but systematic isotope ratio biases when the ion population becomes too large. As ion abundance increases, space-charge effects within the ion optics and analyzer can perturb ion motion and mass accuracy, skewing the quantification of minor isotopologues. This behavior has been documented in other high-precision isotope measurement platforms (for instance, laser ablation MC-ICP-MS), where excessive ion loads induce measurable isotope ratio bias even under carefully controlled conditions.^45^ In extreme cases, when the incoming ion flux is sufficiently high that the accumulated charge surpasses the level intended by AGC control before the minimum injection time is reached, nonideal ion accumulation and partial detector saturation can occur. These effects disproportionately reduce the detection of minor isotopologues during ion collection and transmission, thereby biasing the measured ^13^R downward. Notably, this bias is not random noise but a reproducible, signal-dependent deviation. For example, in gas-phase IRMS instruments, “linearity” calibrations are crucial to mitigate such intensity-dependent errors; a trade-off is often required between sensitivity and accuracy, with instrument parameters (e.g., extraction lens voltage) tuned to ensure a linear isotope response across a range of signal intensities.^46^ The Orbitrap results here are consistent with those phenomena: high-intensity conditions introduce a small yet systematic isotope ratio error due to physical limits in the analyzer’s ion handling.

Additionally, even in sample types with minimal matrix complexity, concentration-dependent deviations in δ^13^C values have been previously reported. For instance, in measurements of aromatic hydrocarbons, δ-values decreased systematically at high mass loads despite the absence of chemical overlap or matrix interferences. These shifts were attributed to subtle instrumental artifacts, such as baseline drift and broader integration windows at elevated intensities, further illustrating how nonideal detector behavior can propagate into isotopic outcomes.^47^

All measurements in this study were conducted with fixed instrument settings across concentrations and with a randomized sample order to minimize procedural bias. Under these controlled conditions, a clear optimum emerges. The δ^13^C heatmap (Figure 10), constructed by comparing THN MIX (without ATC) + NH_4_OH (1%) samples to each concentration of the THN standard, highlights that the 5 μM standard consistently produces δ-values closest to zero, reflecting high accuracy (minimal systematic offset) at this intermediate signal intensity. This observation confirms that the isotope ratio (^13^R) is most stable at an intermediate concentration. It reinforces the idea that both low and high ion populations introduce distinct analytical challenges: statistical limitations in the former and nonlinear or space-charge-related effects in the latter. Additional, detailed trends in δ-values across all experimental conditions are provided in the Supporting Information, further illustrating the interplay between ion-count statistics and instrumental nonlinearity on isotope-ratio precision and accuracy.

**Figure 10:**
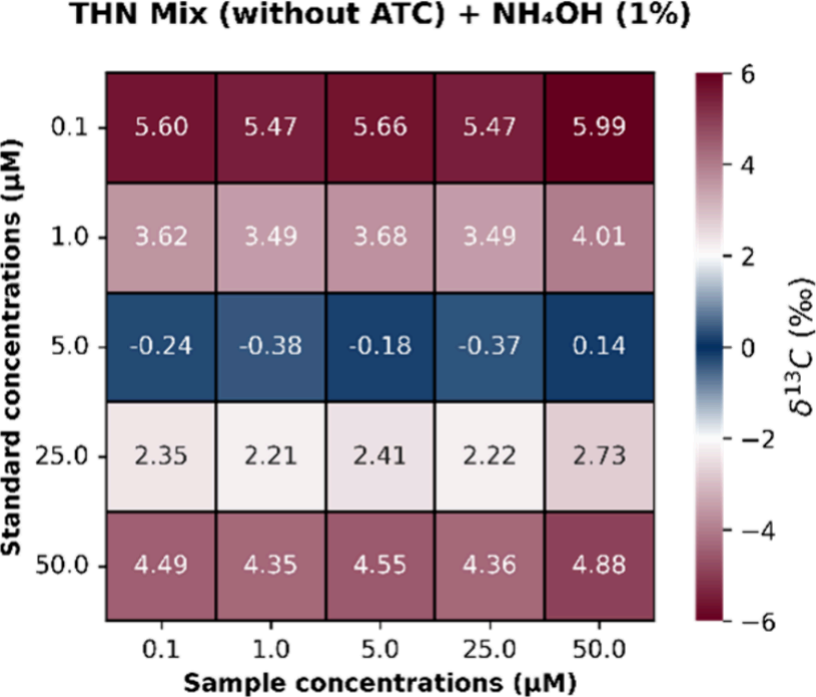
Heatmap of δ^13^C values calculated using the THN Standard as reference and the THN MIX (without ATC) + NH_4_OH (1%) as sample, across all concentration pairs. Blue regions indicate conditions yielding δ-values near zero.

## Conclusion

This study demonstrates that high-resolution Orbitrap mass spectrometry can be effectively employed for stable carbon isotope ratio analysis in complex organic mixtures, using 1,2,3,4-tetrahydro-2-naphthoic acid (THN) as a model naphthenic acid. We showed that accurate ^13^C/^12^C measurements on intact molecules are attainable, but only under carefully controlled ionization conditions. Matrix effects were found to significantly influence isotopic data quality: in the presence of other acids, THN’s ionization efficiency dropped (evidenced by longer injection times), and isotopic precision worsened at low concentrations due to limited ion counts.

Introducing 1% ammonium hydroxide to the matrix improved the situation — although it reduced absolute signal intensity, it promoted a more chemically stable ionization regime, minimizing transient fluctuations in ion generation. At the same time, our complementary analysis of TIC × IT revealed an instrumental threshold behavior: at very low concentrations, the THN-MIX + NH_4_OH system occasionally reached the 100 ms injection time limit, leaving the AGC feedback loop and producing subtarget ion loads. These conditions corresponded precisely to cases of reduced isotopic precision, confirming that loss of AGC regulation can amplify statistical noise when the signal approaches the detection limit. Together, these results emphasize that both chemical and instrumental regimes jointly dictate isotopic precision under matrix-suppressed conditions.

Complementary tests with small organic acids (acetate, propionate, butyrate) and a natural rumen fluid sample showed consistent trends: acetate’s δ^13^C remained constant across varying mixtures despite drastic signal suppression, and only extreme suppression (very high butyrate:acetate ratios in the rumen fluid) caused its δ to diverge. These results confirm that generalized bulk matrix suppression alone does not induce isotope fractionation, whereas very low analyte ion counts (severe suppression) do.

We also found that localized space-charge effects and ion population perturbations within the instrument can specifically perturb isotope measurements. Co-accumulating contaminants, such as an anthracene-carboxylate fragment (*m*/*z* 177.07) in the THN matrix or a carbonate anion (*m*/*z* 59.98) in the acetate experiments, biased the measured δ^13^C when present above ∼ 10% of the analyte signal. Removing or minimizing these ions restored accurate ratios, underscoring that localized space-charge effects from even fully resolved ions—facilitated by narrow quadrupole mass windows—can degrade isotope accuracy more than bulk matrix suppression. In this context, further studies on frequency and intensity shifts due to ion accumulation perturbations in the C-trap and localized space-charge in the Orbitrap analyzer are necessary to fully comprehend these dynamics and improve the efficacy of the isotope ratio analysis.

Importantly, we identified an optimum analyte signal window: very low signal yields poor precision (ion-statistical noise), and very high signal introduces subtle nonlinear biases (space-charge/detector effects). Between these extremes, intermediate signals (∼5 μM for THN in this study) produced the most precise and accurate isotope ratios.

In summary, our findings highlight the feasibility of Orbitrap-MS for precise isotopic analysis of both petroleum-related and small organic acids and delineate the practical challenges that must be managed. Reliable measurements require balancing ion counts (avoiding both starvation and overload) and controlling isobaric interferences. The zero-enrichment bracketing approach (using the same compound as sample and reference) was invaluable for diagnosing biases. This study not only provides the first Orbitrap-MS isotope ratios of a naphthenic acid in a complex mixture, but also suggests strategies (e.g., mild alkalinization, matrix simplification, optimized signal level, contaminant suppression) to maintain accuracy.

## Supplementary Material





## Notes

Additional experimental details supporting Orbitrap isotope-ratio measurements, including chemical composition and structures of the analyzed naphthenic acids, isotopic precision metrics across experimental conditions, complete analytical injection sequence, Orbitrap ion-source parameters, supplementary figures showing isotopic behavior, contaminant identification, chromatograms, and mass spectra, and description of the IsotoPy data-processing workflow (PDF)
Complete IsotoPy analysis report containing processing parameters, total ion chromatograms, ion statistics, outlier filtering, and isotope-ratio results for all injections (PDF)

## References

[1] Ouyang W. Y., Kümmel S., Adrian L., Zhu Y. G., Richnow H. H. (2023). Carbon
and Hydrogen Stable Isotope Fractionation of Sulfamethoxazole during
Anaerobic Transformation Catalyzed by Desulfovibrio Vulgaris Hildenborough. Chemosphere.

[2] Yan X., Li W., Zhu C., Peacock C. L., Liu Y., Li H., Zhang J., Hong M., Liu F., Yin H. (2023). Zinc Stable
Isotope Fractionation Mechanisms during Adsorption on and Substitution
in Iron (Hydr)­Oxides. Environ. Sci. Technol..

[3] Heydarizad M., Raeisi E., Sori R., Gimeno L. (2019). An Overview of the
Atmospheric Moisture Transport Effect on Stable Isotopes (Δ18O,
δ 2H) and D Excess Contents of Precipitation in Iran. Theor. Appl. Climatol..

[4] Arosio T., Büntgen U., Nicolussi K., Moseley G. E., Saurer M., Pichler T., Smith M. P., Gutierrez E., Andreu-Hayles L., Hajdas I., Bebchuk T., Leuenberger M. (2024). Tree-Ring
δ 18O and δ 2H Stable Isotopes Reflect the Global Meteoric
Water Line. Front. Earth Sci. (Lausanne)..

[5] Santos V. H. J. M. d., Pontin D., Engelmann P. d. M., Cescani V. K., Zielinski J. P. T., Barili R., Melo C. L., Dalla Vecchia F. (2024). Exploring
the Potential of Stable Isotope Methods for Identifying the Origin
of CO2 in the Carbonation Process of Cementitious Materials within
the Carbon Capture and Storage Environment. Appl. Geochem..

[6] Hoffman D. W., Rasmussen C. (2024). Position-Specific
Carbon Stable Isotope Analysis of
Glyphosate: Isotope Fingerprinting of Molecules within a Mixture. Anal. Bioanal. Chem..

[7] Li J., Fu X., Bai Y., Zhang H., Liu Z., Zhao R. (2024). Dual Effect
of Hydrothermal Fluid on Shale Oil Reservoir in Gulong Sag, Songliao
Basin: Constrained by C-O Isotope and Geochemistry. Energies (Basel)..

[8] Geist J., Molkentin J., Döring M., Haase I. (2025). Egg Authentication
under Seasonal Variation Using Stable Isotope Analysis Combined with
Machine Learning Classification. Food Control.

[9] Holdsworth C. M., John C. M., Snæbjörnsdóttir S., Johnson G., Sigfússon B., Leslie R., Haszeldine R. S., Gilfillan S. M. V. (2024). Reconstructing the Temperature and Origin of CO2 mineralisation
in CarbFix Calcite Using Clumped, Carbon and Oxygen Isotopes. Appl. Geochem..

[10] Rudra A., Wood J. M., Biersteker V., Sanei H. (2024). Oil Migration from
Internal and External Source Rocks in an Unconventional Hybrid Petroleum
System, Montney Formation, Western Canada. Int.
J. Coal Geol..

[11] Wendt K., Trautmann N. (2005). Recent Developments in Isotope Ratio Measurements by
Resonance Ionization Mass Spectrometry. Int.
J. Mass Spectrom..

[12] Mueller E. P., Sessions A. L., Sauer P. E., Weiss G. M., Eiler J. M. (2022). Simultaneous,
High-Precision Measurements of Δ2H and Δ13C in Nanomole
Quantities of Acetate Using Electrospray Ionization-Quadrupole-Orbitrap
Mass Spectrometry. Anal. Chem..

[13] Zeichner S. S., Wilkes E. B., Hofmann A. E., Chimiak L., Sessions A. L., Makarov A., Eiler J. M. (2022). Methods and Limitations of Stable
Isotope Measurements via Direct Elution of Chromatographic Peaks Using
Gas Chromotography-Orbitrap Mass Spectrometry. Int. J. Mass Spectrom..

[14] Hofmann A. E., Chimiak L., Dallas B., Griep-Raming J., Juchelka D., Makarov A., Schwieters J., Eiler J. M. (2020). Using Orbitrap Mass Spectrometry to Assess the Isotopic
Compositions of Individual Compounds in Mixtures. Int. J. Mass Spectrom..

[15] Neubauer C., Crémière A., Wang X. T., Thiagarajan N., Sessions A. L., Adkins J. F., Dalleska N. F., Turchyn A. V., Clegg J. A., Moradian A., Sweredoski M. J., Garbis S. D., Eiler J. M. (2020). Stable Isotope Analysis of Intact
Oxyanions Using Electrospray Quadrupole-Orbitrap Mass Spectrometry. Anal. Chem..

[16] Gilbert A., Yamada K., Yoshida N. (2013). Accurate Method
for the Determination
of Intramolecular 13C Isotope Composition of Ethanol from Aqueous
Solutions. Anal. Chem..

[17] Eiler J., Cesar J., Chimiak L., Dallas B., Grice K., Griep-Raming J., Juchelka D., Kitchen N., Lloyd M., Makarov A., Robins R., Schwieters J. (2017). Analysis of
Molecular Isotopic Structures at High Precision and Accuracy by Orbitrap
Mass Spectrometry. Int. J. Mass Spectrom..

[18] Eiler J. M., Griep-Raming J., Juchela D., Makarov A. (2023). Analysis of Molecular
Isotopic Structure by Fourier-Transform Mass Spectrometry. Handbook of Isotopologue Biogeochemistry.

[19] Csernica T., Bhattacharjee S., Eiler J. (2023). Accuracy and Precision of ESI-Orbitrap-IRMS
Observations of Hours to Tens of Hours via Reservoir Injection. Int. J. Mass Spectrom..

[20] Silverman S. N., Phillips A. A., Weiss G. M., Wilkes E. B., Eiler J. M., Sessions A. L. (2022). Practical Considerations for Amino Acid Isotope Analysis. Org. Geochem..

[21] Wilkes E. B., Sessions A. L., Zeichner S. S., Dallas B., Schubert B., Jahren A. H., Eiler J. M. (2022). Position-specific Carbon Isotope
Analysis of Serine by Gas Chromatography/Orbitrap Mass Spectrometry,
and an Application to Plant Metabolism. Rapid
Commun. Mass Spectrom..

[22] Zeichner S. S., Chimiak L., Elsila J. E., Sessions A. L., Dworkin J. P., Aponte J. C., Eiler J. M. (2023). Position-Specific Carbon Isotopes
of Murchison Amino Acids Elucidate Extraterrestrial Abiotic Organic
Synthesis Networks. Geochim. Cosmochim. Acta.

[23] Weiss G. M., Sessions A. L., Julien M., Csernica T., Yamada K., Gilbert A., Freeman K. H., Eiler J. M. (2023). Analysis of Intramolecular
Carbon Isotope Distributions in Alanine by Electrospray Ionization
Orbitrap Mass Spectrometry. Int. J. Mass Spectrom..

[24] Bernet N. M., Soldini C., Felder T. M. O., Lapčíková K., Neubauer C., Queen W. L., Kaegi R., Tamburini F., Hofstetter T. B. (2025). Oxygen
Isotope Analyses of Phosphate and Organophosphorus
Compounds by Electrospray Ionization Orbitrap Mass Spectrometry. Anal. Chem..

[25] Walters W. W., Bowen A., Weatherly M. E. (2026). High-Resolution
Mass Spectrometry
for Nitrate Aerosol Isotopologue Quantification: Method Development,
Calibration, and Application to Atmospheric Samples. Anal. Chem..

[26] Wei Z., Wang B., Zhu L., Hong Y., Wang Z., Yan H., Peng Y., Hattori S., Bao H. (2025). A Sub-Liter Pretreatment
Method for Orbitrap-Based Freshwater Phosphate Oxygen Isotope Measurement. Appl. Geochem..

[27] McIntosh O. M., Baczynski A. A., Matney M., McLain H. L., Farnsworth K. K., Dworkin J. P., Glavin D. P., Elsila J. E., Xie H., Freeman K. H. (2025). Stable Nitrogen Isotope Analysis of Amino Acids by
Orbitrap Mass Spectrometry: Application for Extraterrestrial Samples. Rapid Commun. Mass Spectrom..

[28] Phiri T. N., Weatherill J. W., Monford-Sanchez E., Serrano-Contreras J.-I., Melvin C., Kunaka M., Chisenga I., Ngalande P., Mweetwa M. N., Besa E., Haider T., Mandal N., Thompson A. J., Edwards C. A., Bourke C. D., Robertson R. C., Posma J. M., Banda R., Mwiinga M., Kazhila L., Katsidzira L., Bwakura-Dangarembizi M., Amadi B., Garcia-Perez I., Maitland K., Marchesi J. R., Morrison D. J., Frost G., Kelly P. (2024). Novel Gastrointestinal Tools (GI
Tools) for Evaluating Gut Functional Capacity in Adults with Environmental
Enteropathy in Zambia and Zimbabwe: A Cross-Sectional Study Protocol. F1000Res..

[29] dos
Santos G. F., Bevilaqua G. B., Gilbert A., Machado H. G., Julien M., Lima G. S., Lima N. M., Ribeiro J. C. O., Ferreira A. A., Rocha Y. S., Gontijo B. (2025). Advancing Stable Isotope
Analysis with Orbitrap-MS for Fatty Acid Methyl Esters and Complex
Lipid Matrices. J. Am. Soc. Mass Spectrom..

[30] Hilkert A., Böhlke J. K., Mroczkowski S. J., Fort K. L., Aizikov K., Wang X. T., Kopf S. H., Neubauer C. (2021). Exploring the Potential
of Electrospray-Orbitrap for Stable Isotope Analysis Using Nitrate
as a Model. Anal. Chem..

[31] Merder J., Freund J. A., Feudel U., Niggemann J., Singer G., Dittmar T. (2020). Improved Mass Accuracy
and Isotope
Confirmation through Alignment of Ultrahigh-Resolution Mass Spectra
of Complex Natural Mixtures. Anal. Chem..

[32] Mueller E. P., Panehal J., Meshoulam A., Song M., Hansen C. T., Warr O., Boettger J., Heuer V. B., Bach W., Hinrichs K. U., Eiler J. M., Orphan V., Lollar B. S., Sessions A. L. (2024). Isotopic Evidence of Acetate Turnover in Precambrian
Continental Fracture Fluids. Nat. Commun..

[33] Clemente J. S., Fedorak P. M. (2005). A Review of the
Occurrence, Analyses, Toxicity, and
Biodegradation of Naphthenic Acids. Chemosphere..

[34] de
Araújo G. L., dos Santos G. F., Martins R. O., da Silva
Lima G., Medeiros I., de Carvalho R. M., Simas R. C., Sgobbi L. F., Chaves A. R., Vaz B. G. (2022). Electromembrane Extraction of Naphthenic
Acids in Produced Water Followed by Ultra-High-Resolution Mass Spectrometry
Analysis. J. Am. Soc. Mass Spectrom..

[35] Zhao Q. H., Ma S., Wu J. X., Chang W. F., Zhang S. F., Sun X. G., Zhou B., Lun Z. M., Chung K. H., Shi Q. (2023). Molecular
Composition of Naphthenic Acids in a Chinese Heavy Crude Oil and Their
Impacts on Oil Viscosity. Pet. Sci..

[36] Gutierrez-Villagomez J. M., Martyniuk C. J., Xing L., Langlois V. S., Pauli B. D., Blais J. M., Trudeau V. L. (2019). Transcriptome Analysis Reveals That
Naphthenic Acids Perturb Gene Networks Related to Metabolic Processes,
Membrane Integrity, and Gut Function in Silurana (Xenopus) Tropicalis
Embryos. Front. Mar. Sci..

[37] Yang C., Zhang G., Serhan M., Koivu G., Yang Z., Hollebone B., Lambert P., Brown C. E. (2019). Characterization
of Naphthenic Acids in Crude Oils and Refined Petroleum Products. Fuel.

[38] Xu X., Pliego G., Zazo J. A., Liu S., Casas J. A., Rodriguez J. J. (2018). Two-Step Persulfate and Fenton Oxidation
of Naphthenic
Acids in Water. J. Chem. Technol. Biotechnol..

[39] Neubauer C., Kantnerová K., Lamothe A., Savarino J., Hilkert A., Juchelka D., Hinrichs K. U., Elvert M., Heuer V., Elsner M., Bakkour R., Julien M., Öztoprak M., Schouten S., Hattori S., Dittmar T. (2023). Discovering Nature’s
Fingerprints: Isotope Ratio Analysis on Bioanalytical Mass Spectrometers. J. Am. Soc. Mass Spectrom..

[40] Kantnerová K., Kuhlbusch N., Juchelka D., Hilkert A., Kopf S., Neubauer C. (2024). A Guide to
Precise Measurements of Isotope Abundance
by ESI-Orbitrap MS. Nat. Protoc..

[41] Makarov A., Denisov E. (2009). Dynamics of Ions of
Intact Proteins in the Orbitrap
Mass Analyzer. J. Am. Soc. Mass Spectrom..

[42] Henriksen T., Juhler R. K., Svensmark B., Cech N. B. (2005). The Relative Influences
of Acidity and Polarity on Responsiveness of Small Organic Molecules
to Analysis with Negative Ion Electrospray Ionization Mass Spectrometry
(ESI-MS). J. Am. Soc. Mass Spectrom..

[43] Monnin C., Ramrup P., Daigle-Young C., Vuckovic D. (2018). Improving Negative
Liquid Chromatography/Electrospray Ionization Mass Spectrometry Lipidomic
Analysis of Human Plasma Using Acetic Acid as a Mobile-phase Additive. Rapid Commun. Mass Spectrom..

[44] Heumann K. G., Gallus S. M., Rädlinger G., Vogl J. (1998). Precision and Accuracy
in Isotope Ratio Measurements by Plasma Source Mass Spectrometry †. J. Anal. At. Spectrom..

[45] Standish C. D., Milton J. A., Brown R. M., Foster G. L. (2025). Matrix Independent
and Interference Free in Situ Boron Isotope Analysis by Laser Ablation
MC-ICP-MS/MS. J. Anal. At. Spectrom..

[46] Good Practice Guide for Isotope Ratio Mass Spectrometry, 2nd ed.; Dunn, P. J. H. , Carter, J. F. , Eds.; FIRMS: 2018. 10.1080/10256016.2018.1531495 30354799

[47] Kornilova A., Moukhtar S., Saccon M., Huang L., Zhang W., Rudolph J. (2015). A Method for
Stable Carbon Isotope Ratio and Concentration
Measurements of Ambient Aromatic Hydrocarbons. Atmos. Meas. Technol..

