# Supplementary material for: Lessons Learned in Orbitrap MS-Based Isotope Ratio Analysis of Organic Acid Mixtures
Source: Anal Chem. 2026 Mar 21;98(13):9764–75. doi: 10.1021/acs.analchem.5c07111 (PMC13063220; doi:10.1021/acs.analchem.5c07111)
Supplement: Supplementary file 1 [file ac5c07111_si_001.pdf]

Supporting Information

Lessons Learned in Orbitrap MS–Based Isotope Ratio Analysis of Organic Acid Mixtures

Hugo G. Machado [a*], Elliott P. Mueller [b,c], Júlio C. O. Ribeiro [a], Giovanni B. Bevilaqua [a], Gabriel F. dos Santos[a], Alexandre A. Ferreira [d], Ygor S. Rocha [d], Surjyendu Bhattacharjee [b], John M. Eiler [b], Boniek Gontijo [a*]

[a] Chemistry Institute, Federal University of Goiás, Goiânia, Goiás,74690-900, Brazil

[b] Division of Geological and Planetary Sciences, California Institute of Technology, Pasadena, CA, 91125, USA

[c] Departament of Geological Sciences, University of Colorado Boulder, Boulder, CO, 80205, USA

[d] Division of Geochemistry, PETROBRAS Research and Development Center (CENPES), PETROBRAS, Rua Horácio Macedo, Ilha do Fundão, Rio de Janeiro, RJ, 21941-915, Brazil

*Corresponding author.
E-mail address: hugogontijomachado@gmail.com (H. G. Machado)
E-mail address: boniek@ufg.br (B. Gontijo)

Table of contents:

Table S1: Representation of all Chemicals, molecular formulas, exact masses, pKa's and structures of naphthenic acids used in this work S4

Table S2: Summary of isotopic precision metrics across experimental conditions. The first column lists the four tested systems. For each system, results are reported at five THN concentrations (0.1, 1.0, 5.0, 25.0, and 50.0 µmol L^{-1}). The following columns present the acquisition error (relative standard error) of the ^{13}R (‰), the theoretical shot-noise limit (‰), and the ratio between acquisition error and shot-noise, which serves as an indicator of the influence of non-statistical sources of variability. S5

Table S3: Complete sequence of 65 injections. Solution (A): THN Standard; (B): THN-Mix; (C): THN-Mix + NH_4OH (1%); (D): THN-Mix (without ATC) + NH_4OH (1%)....6

Table S4: Ion source settings for acetate-propianate-butyrate system in Orbitrap Exploris 240 MS S7

Figure S1: Isotopic ratio (^{13}R) plotted as a function of analyte concentration (0.1–50%µM) for each of the four tested systems: (A) THN Standard, (B) THN MIX, (C) THN MIX + NH_4OH, and (D) THN MIX without ATC + NH_4OH. Each data point represents the mean ^{13}R value across replicate injections, with error bars denoting standard error. A dashed gray line indicates the mean ^{13}R value of the THN Standard at 5%µM, identified as the optimal concentration for isotopic stability. Systems with ^{13}R values approaching this

reference line are those that achieved higher accuracy in δ¹³C measurements (i.e., $\delta^{13}C \approx 0$)..S8
Figure S2: Molecular structure of the matrix-derived contaminant ion at *m/z* 177.07, identified as 9H-anthracen-9-ylium ($C_{14}H_9^+$), a fragment from 9-anthracenecarboxylic acid (ATC)..S8
Figure S3: Heatmaps of $\delta^{13}C$ values for three experimental systems: A) THN MIX, B) THN MIX + NH_4OH (1%), and C) THN MIX (without ATC) + NH_4OH (1%). Each heatmap shows $\delta^{13}C$ values calculated using the THN Standard as reference and the respective system as sample, across all concentration pairs. Blue regions indicate conditions yielding δ-values near zero, denoting high isotopic agreement with the standard. Red-shifted regions reflect systematic isotopic deviations. The THN MIX system exhibits a more random distribution of δ-values across concentrations. In contrast, the THN MIX (without ATC) + NH_4OH (1%) condition shows a clearer trend toward accurate $\delta^{13}C$ values centered around the 5 μM reference, highlighting the combined benefits of matrix simplification and mild alkalinization for improving isotopic fidelity. S9
Figure S4: a) Representative high-resolution mass spectra of acetate, propionate, and butyrate, the small organic acids present in the analyzed mixture. Insets highlights the monoisotopic and isotopologue peaks. b) Total ion chromatograms (TICs) for each of the three acids within a single analytical block (16min), showing the sequential switching of acquisition windows (57-62, 72-75, and 86-90 m/z). c) TIC of the complete analytical sequence, comprising seven alternating standard-sample blocks and totaling 112min. S10
Figure S5: Three-dimensional scatter plot of $\delta^{13}C$ values measured for acetate, propionate, and butyrate in a rumen fluid sample. Each axis corresponds to the $\delta^{13}C$ composition of one the three shot-chain organic acids. Marker colors represent the butyrate:acetate ion current ratio. Under typical conditions (ratios ≤ 5), $\delta^{13}C$ values of all acids cluster within a consistent range. In contrast, when the butyrate:acetate ratio exceeds ~5, a clear deviation is observed in the acetate $\delta^{13}C$ values, indicating that severe ion suppression drives the measurement outside of the isotopic stability regime..............S11
Figure S6: Relationship between analyte concentration and the product of total ion current and injection time (TIC×IT) for the THN standard, THN-Mix, and THN-Mix + NH_4OH (1%) systems. Each data point represents a replicate acquisition, with error bars indicating the standard deviation across scans. For the THN standard and THN-Mix, TIC×IT values remained close to the AGC target (~ 1.1×10^6, in arbitrary units) over the entire concentration range, confirming stable AGC-controlled ion accumulation. In contrast, for the THN-Mix + NH_4OH system, all three replicates at 1 μM and the first two at 0.1 μM fell well below the expected target, reflecting operation ath the 100ms injection-time limit. The deviation was most pronounced for replicates 1 at 0.1 μM (2.07×10^5) and at 1 μM (4.94×10^5), confirming that these injections were no longer under effective AGC regulation. In contrast, replicate 3 at 0.1 μM maintained TIC×IT values near the AGC

target and injection times below 100ms, consistent with its higher isotopic precision (see Figure 3). S11

Supplementary Text: Data Processing Details ………………………………………...…S11

References…………………………………………………………………………….S12

Table S1: Representation of all Chemicals, molecular formulas, exact masses, pKa's and structures of naphthenic acids used in this work

**Chemical**	**Molecular Formula**	**Exact mass**	**pKa**	**Structure**
9-Anthracenecarboxylic acid	$C_{15}H_{10}O_2$	222.0680	3.65	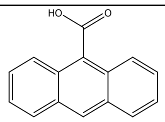
1-Naphthoic acid	$C_{11}H_8O_2$	172.0524	3.70	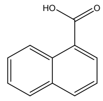
Benzoic acid	$C_7H_6O_2$	122.0367	4.17	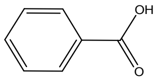
1-Naphthaleneacetic acid	$C_{12}H_{10}O_2$	186.0680	4.23	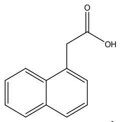
Cyclohexylacetic acid	$C_8H_{14}O_2$	142.0993	4.51	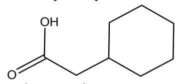
1,2,3,4-Tetrahydro-2-naphthoic acid	$C_{11}H_{12}O_2$	176.0837	4.57	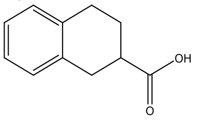
2-Methyloctadecanoic acid	$C_{19}H_{38}O_2$	298.2871	4.60	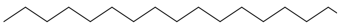
Decanoic acid	$C_{10}H_{20}O_2$	172.1463	4.79	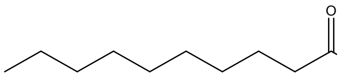
Dicyclohexylacetic acid	$C_{14}H_{24}O_2$	224.1776	4.81	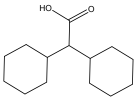
3,5-Dimethyladamantane-1-carboxylic acid	$C_{13}H_{20}O_2$	208.1463	4.88	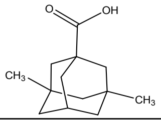

Table S2: Summary of isotopic precision metrics across experimental conditions. The first column lists the four tested systems. For each system, results are reported at five THN concentrations (0.1, 1.0, 5.0, 25.0, and 50.0 µmol L^{-1}). The following columns present the acquisition error (relative standard error) of the ^{13}R (‰), the theoretical shot-noise limit (‰), and the ratio between acquisition error and shot-noise, which serves as an indicator of the influence of non-statistical sources of variability.

	**Concentration (µM)**	**Acquisition Error (‰)**	**Shot-Noise (‰)**	**Acquisition Error / Shot-Noise**
THN Standard	0.1	0.174543	0.161173	1.083286
	1	0.170321	0.159898	1.065227
	5	0.169314	0.160343	1.056088
	25	0.170116	0.160320	1.061255
	50	0.170531	0.160033	1.066103
THN Mix	0.1	0.804716	0.691066	1.172990
	1	0.256434	0.189394	1.354026
	5	0.187967	0.158690	1.184366
	25	0.173632	0.158032	1.098791
	50	0.169095	0.158278	1.068637
THN Mix + NH4 (1%)	0.1	0.632376	0.473612	1.254522
	1	0.295838	0.242254	1.213087
	5	0.188576	0.167990	1.122098
	25	0.171068	0.159251	1.075298
	50	0.177960	0.164302	1.083839
THN Mix (without ATC) + NH_4 (1%)	0.1	0.168278	0.154671	1.087914
	1	0.168931	0.157194	1.074912
	5	0.165256	0.154859	1.067925
	25	0.169100	0.158906	1.065208
	50	0.169788	0.159812	1.063899

Table S3: Complete sequence of 65 injections. Solution **(A)**: THN Standard; **(B)**: THN-Mix; **(C)**: THN-Mix + NH_4OH (1%); **(D)**: THN-Mix (without ATC) + NH_4OH (1%).

Replicate	Solution	Concentration	Replicate	Solution	Concentration
1	C	0.1	2	A	25
1	A	0.1	2	C	25
1	D	0.1	2	B	25
1	B	0.1	2	C	50
1	A	1	2	A	50
1	C	1	2	D	50
1	D	1	2	B	50
1	B	1	3	C	0.1
1	C	5	3	B	0.1
1	A	5	3	A	0.1
1	D	5	3	D	0.1
1	B	5	3	D	1
1	A	25	3	B	1
1	C	25	3	A	1
1	B	25	3	C	1
1	D	25	3	D	5
1	B	50	3	B	5
1	D	50	3	A	5
1	A	50	3	C	5
1	C	50	3	B	25
2	C	0.1	3	A	25
2	D	0.1	3	C	25
2	A	0.1	3	D	25
2	B	0.1	3	A	50
2	C	1	3	B	50
2	A	1	3	C	50
2	B	1	3	D	50
2	D	1	4	A	0.1
2	C	5	4	A	1
2	D	5	4	A	5
2	B	5	4	A	25
2	A	5	4	A	50
2	D	25			

Table S4: Ion source settings for acetate-propianate-butyrate system in Orbitrap Exploris 240 MS

Ion source		Define scan	
Sheath gas (Arb.)	5 (Typical range 1-10)	Scan type	Full scan
Aux gas (Arb)	1 (Typical range 1-10)	Orbitrap resolution	60,000
Sweep gas (Arb.)	1 or 0	Polarity	Negative
Neg ion spray voltage	3000 V (Typical range 2800-3200 V)	Microscans	1
Spray current (observed)	<0.3 μA	Maximum injection time (ms)	1000
Ion transfer tube temp (ºC)	320	RF lens (%)	50
		AGC target	Standard (equals 1E6 absolute AGC target)

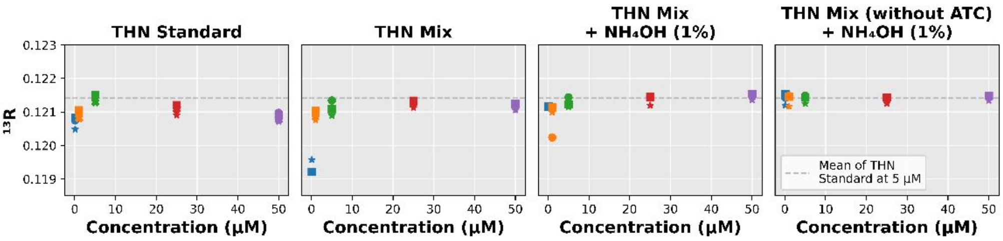

Figure S1: Isotopic ratio (^{13}R) plotted as a function of analyte concentration (0.1–50 µM) for each of the four tested systems: (A) THN Standard, (B) THN MIX, (C) THN MIX + NH_4OH, and (D) THN MIX without ATC + NH_4OH. Each data point represents the mean ^{13}R value across replicate injections, with error bars denoting standard error. A dashed gray line indicates the mean ^{13}R value of the THN Standard at 5 µM, identified as the optimal concentration for isotopic stability. Systems with ^{13}R values approaching this reference line are those that achieved higher accuracy in $\delta^{13}C$ measurements (i.e., $\delta^{13}C \approx 0$).

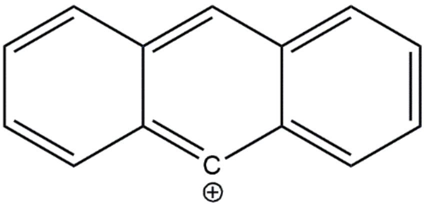

Figure S2: Molecular structure of the matrix-derived contaminant ion at *m/z* 177.07, identified as 9H-anthracen-9-ylium ($C_{14}H_9^+$), a fragment from 9-anthracenecarboxylic acid (ATC).

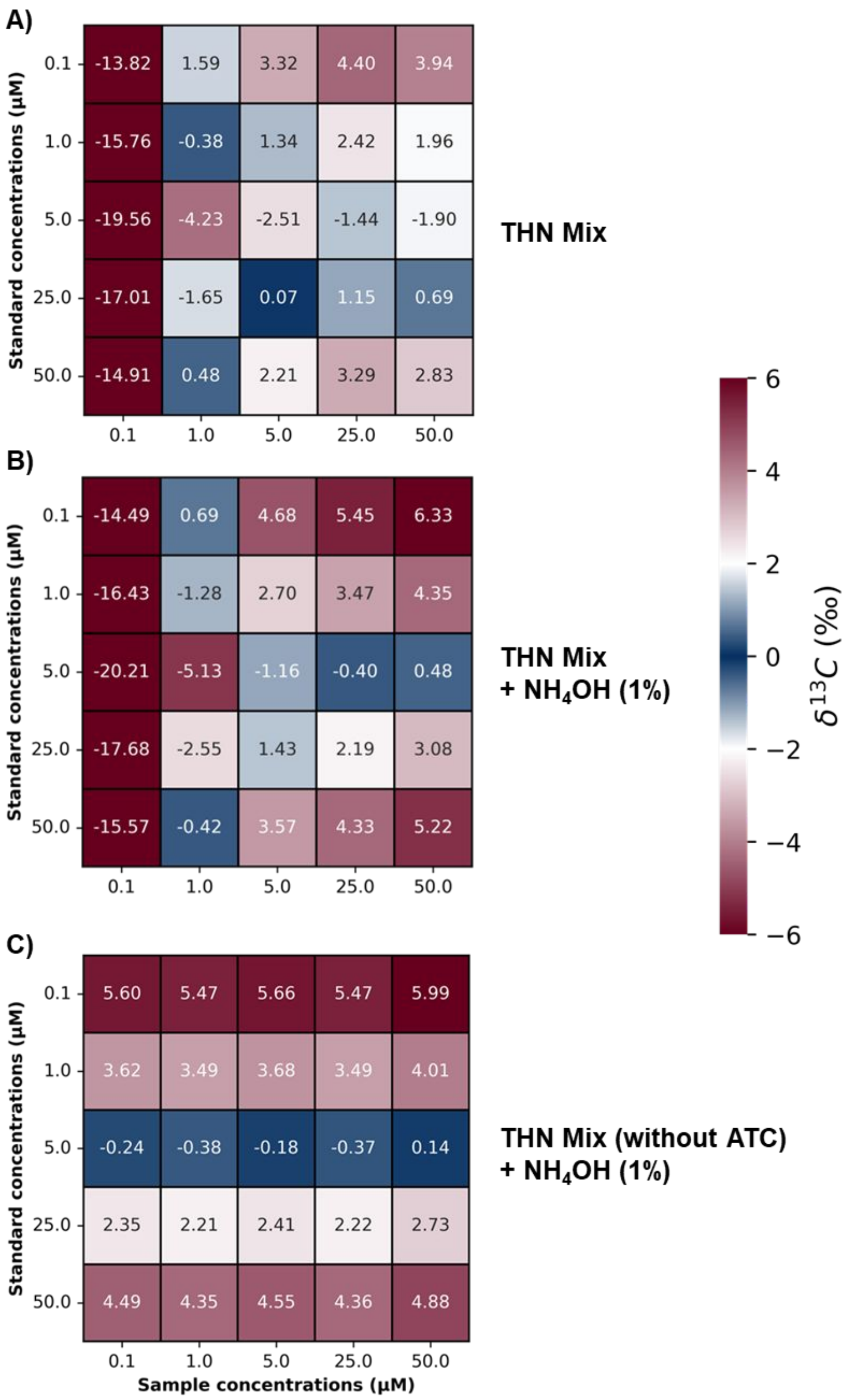

Figure S3: Heatmaps of δ^{13}C values for three experimental systems: A) THN MIX, B) THN MIX + NH_4OH (1%), and C) THN MIX (without ATC) + NH_4OH (1%). Each heatmap shows δ^{13}C values calculated using the THN Standard as reference and the respective system as sample, across all concentration pairs. Blue regions indicate conditions yielding δ-values near zero, denoting high isotopic agreement with the standard. Red-shifted regions reflect systematic isotopic deviations. The THN MIX system exhibits a more random distribution of δ-values across concentrations. In contrast, the THN MIX (without ATC) + NH_4OH (1%) condition shows a clearer trend toward accurate δ^{13}C values centered around the 5 μM reference, highlighting the combined benefits of matrix simplification and mild alkalinization for improving isotopic fidelity.

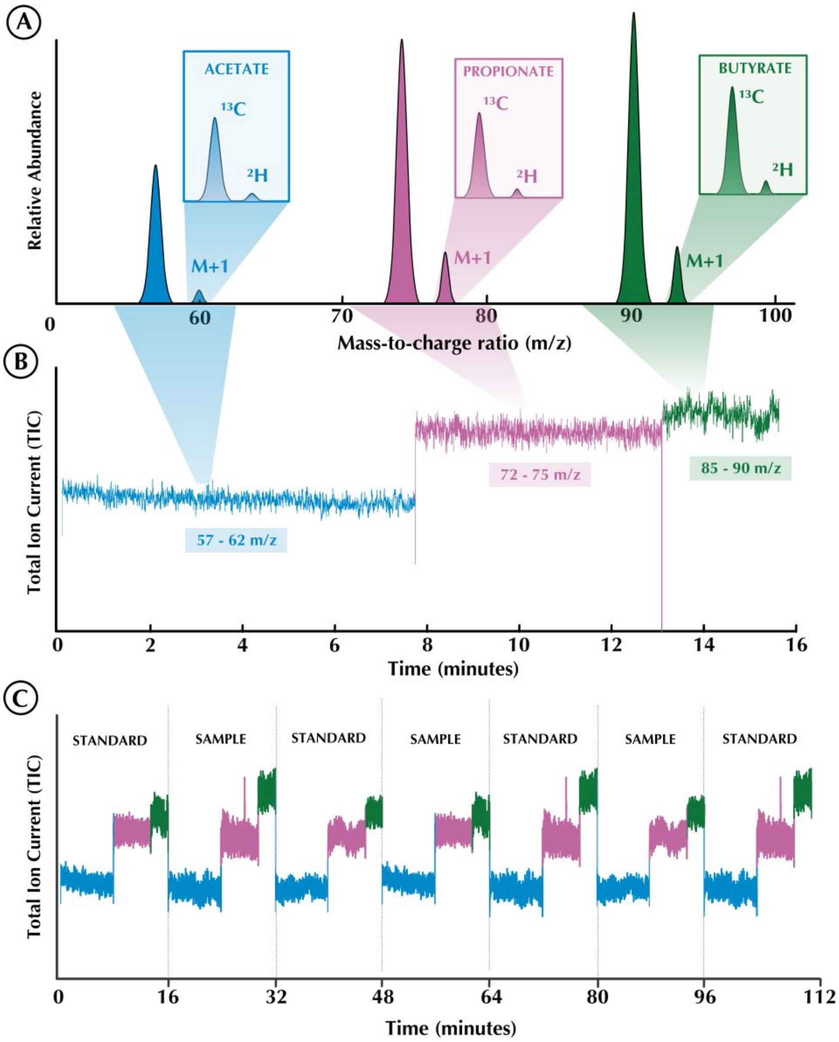

Figure S4: a) Representative high-resolution mass spectra of acetate, propionate, and butyrate, the small organic acids present in the analyzed mixture. Insets highlights the monoisotopic and isotopologue peaks. b) Total ion chromatograms (TICs) for each of the three acids within a single analytical block (16min), showing the sequential switching of acquisition windows (57-62, 72-75, and 86-90 m/z). c) TIC of the complete analytical sequence, comprising seven alternating standard-sample blocks and totaling 112min.

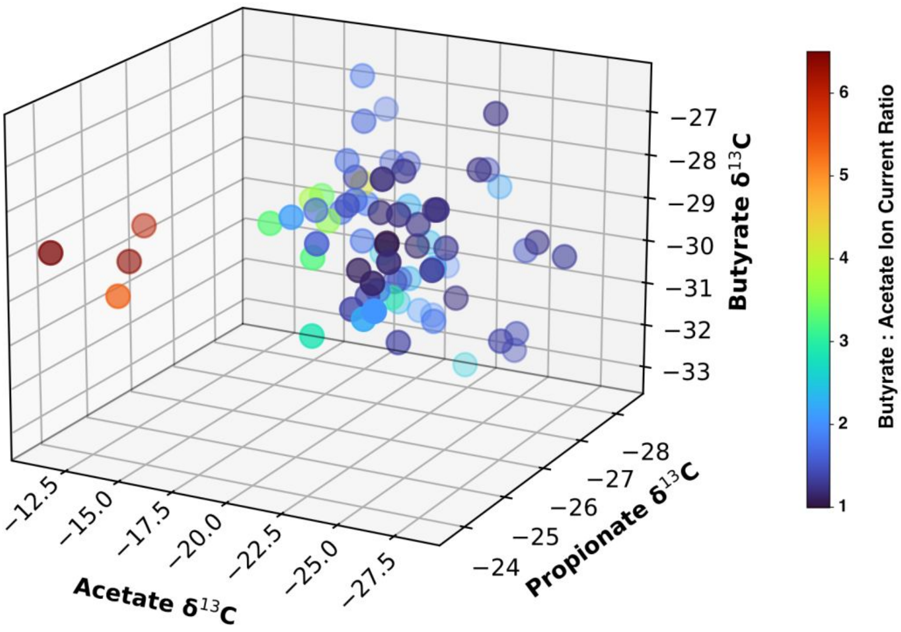

Figure S5: Three-dimensional scatter plot of $\delta^{13}C$ values measured for acetate, propionate, and butyrate in a rumen fluid sample. Each axis corresponds to the $\delta^{13}C$ composition of one the three shot-chain organic acids. Marker colors represent the butyrate:acetate ion current ratio. Under typical conditions (ratios ≤ 5), $\delta^{13}C$ values of all acids cluster within a consistent range. In contrast, when the butyrate:acetate ratio exceeds ~5, a clear deviation is observed in the acetate $\delta^{13}C$ values, indicating that severe ion suppression drives the measurement outside of the isotopic stability regime.

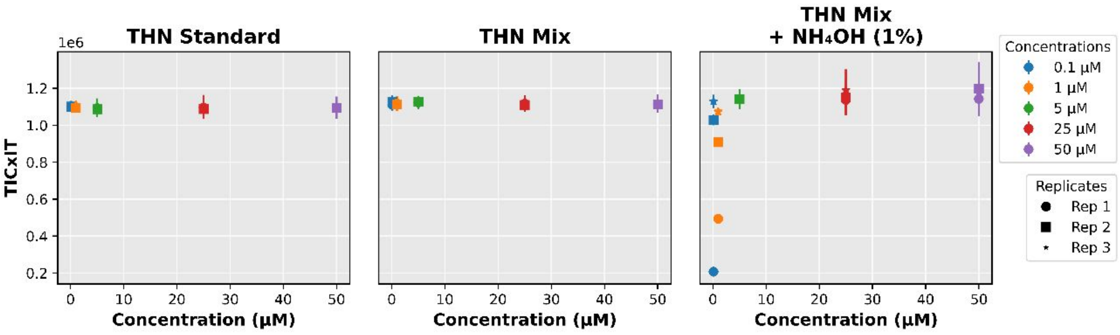

Figure S6: Relationship between analyte concentration and the product of total ion current and injection time (TIC×IT) for the THN standard, THN-Mix, and THN-Mix + NH_4OH (1%) systems. Each data point represents a replicate acquisition, with error bars indicating the standard deviation across scans. For the THN standard and THN-Mix, TIC×IT values remained close to the AGC target (~ 1.1×10^6, in arbitrary units) over the entire concentration range, confirming stable AGC-controlled ion accumulation. In contrast, for the THN-Mix + NH_4OH system, all three replicates at 1 µM and the first two at 0.1 µM fell well below the expected target, reflecting operation ath the 100ms injection-time limit. The deviation was most pronounced for replicates 1 at 0.1 µM (2.07×10^5) and at 1 µM (4.94×10^5), confirming that these injections were no longer under effective AGC regulation. In contrast, replicate 3 at 0.1 µM maintained TIC×IT values near the AGC target and injection times below 100ms, consistent with its higher isotopic precision (see Figure 3).

Data Processing Details

As described in the main text, *IsotoPy* is an in-house software developed by our research group that performs parameter extraction using the Thermo Fisher Scientific RawFileReader library and calculates ion counts using the same equation as the IsoX software. This section provides additional details on the subsequent steps of data processing.

After extracting relevant scan prameters and computing ion counts, *IsotoPy* generates a '.isopy' file, which is structurally similar to the '.isox' files produced by Thermo Fisher's IsoX software. Consequently, users can initiate processing using any of the following file types: '.raw', '.isopy' or '.isox'. If the user opts to begin with a '.raw' file, it is necessary to provide a '.tsv' file that specifies the *m/z* of the isotopologues of interest, their respective charges states, and the mass tolerance window for ion identification in each scan. Depending on the analysis methodology, input format differs:

- For Dual Inlet experiments, the user provides a single file and must specify the number of blocks and the duration of each block.
- For Flow Injection (direct HPLC injection without a chromatographic column), the user must provide one file per block, corresponding to the individual sample or standard injections

Prior to full processing, the data is typically trimmed to remove portions of the chromatogram affected by injection artifacts or signal instability. In Dual Inlet mode, the first minute of each block—known as the switch time—is removed to eliminate artifacts from valve switching. In Flow Injection mode, both the first minutes (dead volume) and the final minutes (post-elution baseline) are excluded, as they correspond to low or unstable signal regions. In this study, all analysis were conducted in Flow Injection method, and each injection was conducted over a 15-minute period, and the chromatogram was trimmed between minutes 2 and 8.

A recurring feature in high-resolution isotope analysis is the occurrence of "zero scans", in which one of the isotopologues is not detected. *IsotoPy* offers two strategies for handling these scans: (1) remove the scan entirely, or (2) assign a zero-intensity value to the missing isotopologue, as proposed by Csernica et al. (2023)[1]. In this study, zero scans were excluded from further processing.

Within the retained spectral range, *IsotoPy* performs outlier scan removal. Mulple filtering strategies are available in the software. For this study, we used the Median Absolute Deviation (MAD) Score method, applying a 2-MAD threshold with a sliding window.

After preprocessing, the isotopic ratio is calculated on a scan-by-scan basis as the ratio between ion counts of the relevant isotopologues. An average isotopic ratio is then computed for each block.

The *IsotoPy* software offers two strategies for δ calculation:

1. In the 'simple bracketing' mode, the average isotope ratio of a given sample block is compared to the average of its immediately adjacent standard blocks, and δ-value is computed accordingly[2].
2. Alternatively, the 'standardization' mode employs a model-based approach in which a linear regression is constructed using the average isotope ratios of all standard blocks (typically blocks 1, 3, 5 and 7). This regression is then used to predict the expected isotope ratios for the sample blocks (e.g., blocks 2, 4 and 6). The predicted standard values are compared to the measured sample ratios, and δ-values are calculated accordingly[1].

In both cases, the final δ-value reported corresponds to the average of the three individual δ-values. The standard deviation among these values is referred to as the error of reproducibility.

In this work, the 'standardization' strategy was used to mitigate potential temporal drifts, as it leverages a broader time-resolved normalization framework using multiple virtual brackets.

A PDF report generated by the *IsotoPy* software is included in the Supporting Information and provides a comprehensive overview of the data analysis pipeline described herein. The report is divided into four main sections:

1. Data Import and User Parameters: This section documents all analysis parameters selected by the user, including those related to the extraction of isotopologue-specific ion data from the raw file (.raw). It provides a clear summary of the input settings that guided the data processing.

2. Pre-processing: This section details the chromatographic trimming and outlier removal steps. It includes a summary table reporting, for each injection: the number of valid scans retained, the number of outlier scans removed, the mean total ion current (TIC), and the TIC relative standard deviation (RSD%). Additionally, a diagnostic plot is provided for each injection, illustrating the selected chromatographic window and the scans identified as outliers.

3. Isotope Ratio Profile: This section evaluates the behavior of the isotopic ratio across the trimmed chromatogram. It includes a table with the total effective ion count per injection, the average isotopic ratio, the relative standard error of the isotopic ratio (acquisition error), the shot-noise limit, and the acquisition error-to-shot-noise ratio. For each injection, two plots are shown: one displaying the cumulative average of the isotopic ratio along the chromatographic time, along with a histogram of its distribution; and a second plot showing the shot-noise and acquisition-error across the injection.

4. Virtual Bracketing and Delta Calculation Summary: This section presents the δ-calculation results for each virtual bracketed acquisition (set of 7 blocks) across the three THN-Mix systems: (i) THN-Mix; (ii) THN-Mix + 1% NH_4OH; and (iii) THN-Mix (without ATC) + 1% NH_4OH. Each of the five Mix concentrations (0.1, 1, 5, 25 and 50 µM) is compared to each of the five THN-Standard concentrations, totaling 25 results per system. For each δ-computation, three plots are provided:

   - The first plot shows the 7-block bracketed acquisition, where odd-numbered blocks (in blue) correspond to the THN-Standard and even-numbered blocks (in red) to the Mix sample. The acquisition error shown corresponds to the average across all seven blocks.

   - The second plot displays the three δ-values calculated using the 'standardization' strategy described earlier. The propagated acquisition error (σ_{PAE}) is also shown, computed following the methodology proposed by Csernica et al. (2023)[1].

   - The third plot represents the average of the three δ-values. The standard deviation among them, referred to as the reproducibility error (ER), is also reported.

References

(1) Csernica, T.; Bhattacharjee, S.; Eiler, J. Accuracy and Precision of ESI-Orbitrap-IRMS Observations of Hours to Tens of Hours via Reservoir Injection. *Int J Mass Spectrom* 2023, *490*. https://doi.org/10.1016/j.ijms.2023.117084.

(2) Hilkert, A.; Böhlke, J. K.; Mroczkowski, S. J.; Fort, K. L.; Aizikov, K.; Wang, X. T.; Kopf, S. H.; Neubauer, C. Exploring the Potential of Electrospray-Orbitrap for Stable

Isotope Analysis Using Nitrate as a Model. *Anal Chem* 2021, *93* (26), 9139–9148. https://doi.org/10.1021/acs.analchem.1c00944.
