# Supplementary material for: Lessons Learned in Orbitrap MS-Based Isotope Ratio Analysis of Organic Acid Mixtures
Source: Anal Chem. 2026 Mar 21;98(13):9764–75. doi: 10.1021/acs.analchem.5c07111 (PMC13063220; doi:10.1021/acs.analchem.5c07111)

Naphthenic Acids Mix
Isotope Analysis report from IsotoPy

Flow Injection

1. Data Import and User Parameters

The following parameters were used as inputs:

parameter	setting
compound	THN-
isotopologues	M0, 13C
most_abundant_isotopologue	M0
harmonization_rule	exclude
ratios	13C/M0
cut_bounds	(2, 8)
remove_outliers_method	mad, N=2
outliers_removed_col	tic
ratio_method	mean

The following parameters were used to extract data from raw files:

Compound	Isotopologue	Exact Mass	Tolerance (mmu)	Ion Charge
THN-	M0	175.07578	0.001	1
THN-	13C	176.07947	0.001	1

2. Pre Processing

Pre processing information for each analysis. Solution (A): THN Standard; Solution (B): THN Mix; Solution (C): THN Mix + NH4OH (1%); Solution (D): THN Mix (without ATC) + NH4OH (1%).

Replicate-Solution-Concentration	Number of scans	Number of outlier scans removed	TIC mean	TIC RSD (%)
1-C-0.1	1268	273	2.07e+06	4.28
1-A-0.1	2140	477	3.11e+07	1.69
1-D-0.1	2349	267	3.27e+07	8.02
1-B-0.1	1288	282	1.14e+07	2.65
1-A-1.0	2142	475	5.75e+07	1.51
1-C-1.0	1295	246	4.94e+06	3.87
1-D-1.0	2283	334	4.60e+07	10.71
1-B-1.0	2081	482	2.05e+07	2.03
1-C-5.0	1722	319	1.56e+07	2.19
1-A-5.0	2119	498	3.16e+08	2.21
1-D-5.0	2236	380	5.40e+07	6.36
1-B-5.0	2167	450	6.38e+07	1.92
1-A-25.0	2127	490	9.43e+08	2.87
1-C-25.0	2304	313	5.81e+07	4.25
1-B-25.0	2130	486	2.13e+08	1.93
1-D-25.0	2059	557	1.03e+08	5.67
1-B-50.0	2166	450	3.92e+08	2.23
1-D-50.0	2036	581	1.75e+08	5.77
1-A-50.0	2165	452	1.32e+09	3.21
1-C-50.0	2115	501	1.19e+08	3.96
2-C-0.1	1301	241	1.03e+07	2.82
2-D-0.1	2206	411	3.55e+07	3.38
2-A-0.1	2090	526	3.41e+07	1.58
2-B-0.1	1544	310	1.38e+07	2.16
2-C-1.0	1283	259	9.08e+06	1.94
2-A-1.0	2210	407	4.76e+07	2.18

Replicate-Solution-Concentration	Number of scans	Number of outlier scans removed	TIC mean	TIC RSD (%)
2-B-1.0	2100	467	2.04e+07	1.85
2-D-1.0	2119	497	4.88e+07	6.56
2-C-5.0	1963	498	1.97e+07	2.27
2-D-5.0	2452	165	5.03e+07	8.29
2-B-5.0	2157	460	6.60e+07	1.85
2-A-5.0	2160	456	3.08e+08	2.22
2-D-25.0	2488	129	9.12e+07	8.86
2-A-25.0	2154	463	8.57e+08	3.31
2-C-25.0	2240	377	6.36e+07	4.41
2-B-25.0	2145	472	2.22e+08	2.00
2-C-50.0	1895	722	1.31e+08	3.58
2-A-50.0	2161	455	1.43e+09	3.03
2-D-50.0	2369	248	1.56e+08	8.52
2-B-50.0	2131	486	4.37e+08	2.47
3-C-0.1	1408	305	1.27e+07	2.47
3-B-0.1	1496	358	1.38e+07	1.86
3-A-0.1	2153	463	3.28e+07	1.32
3-D-0.1	2245	372	3.52e+07	3.52
3-D-1.0	2108	508	5.17e+07	6.50
3-B-1.0	2174	431	2.15e+07	1.79
3-A-1.0	2164	452	5.90e+07	1.45
3-C-1.0	1256	286	1.07e+07	1.72
3-D-5.0	2098	519	5.28e+07	4.66
3-B-5.0	2150	467	6.46e+07	1.75
3-A-5.0	2139	477	3.33e+08	2.43
3-C-5.0	2193	403	2.25e+07	2.45
3-B-25.0	2171	446	2.45e+08	2.08
3-A-25.0	2147	470	1.01e+09	3.12
3-C-25.0	1888	729	7.54e+07	2.88
3-D-25.0	1942	675	9.47e+07	4.04
3-A-50.0	2125	492	1.44e+09	2.82
3-B-50.0	2167	450	4.11e+08	2.21

Replicate-Solution-Concentration	Number of scans	Number of outlier scans removed	TIC mean	TIC RSD (%)
3-C-50.0	1940	676	1.57e+08	2.63
3-D-50.0	1931	685	1.58e+08	4.68
4-A-0.1	2133	483	3.78e+07	1.27
4-A-1.0	2141	475	5.19e+07	2.04
4-A-5.0	2126	491	3.07e+08	2.23
4-A-25.0	2175	442	1.02e+09	3.06
4-A-50.0	2173	443	1.43e+09	3.02

Total Ion Chromatogram (TIC)

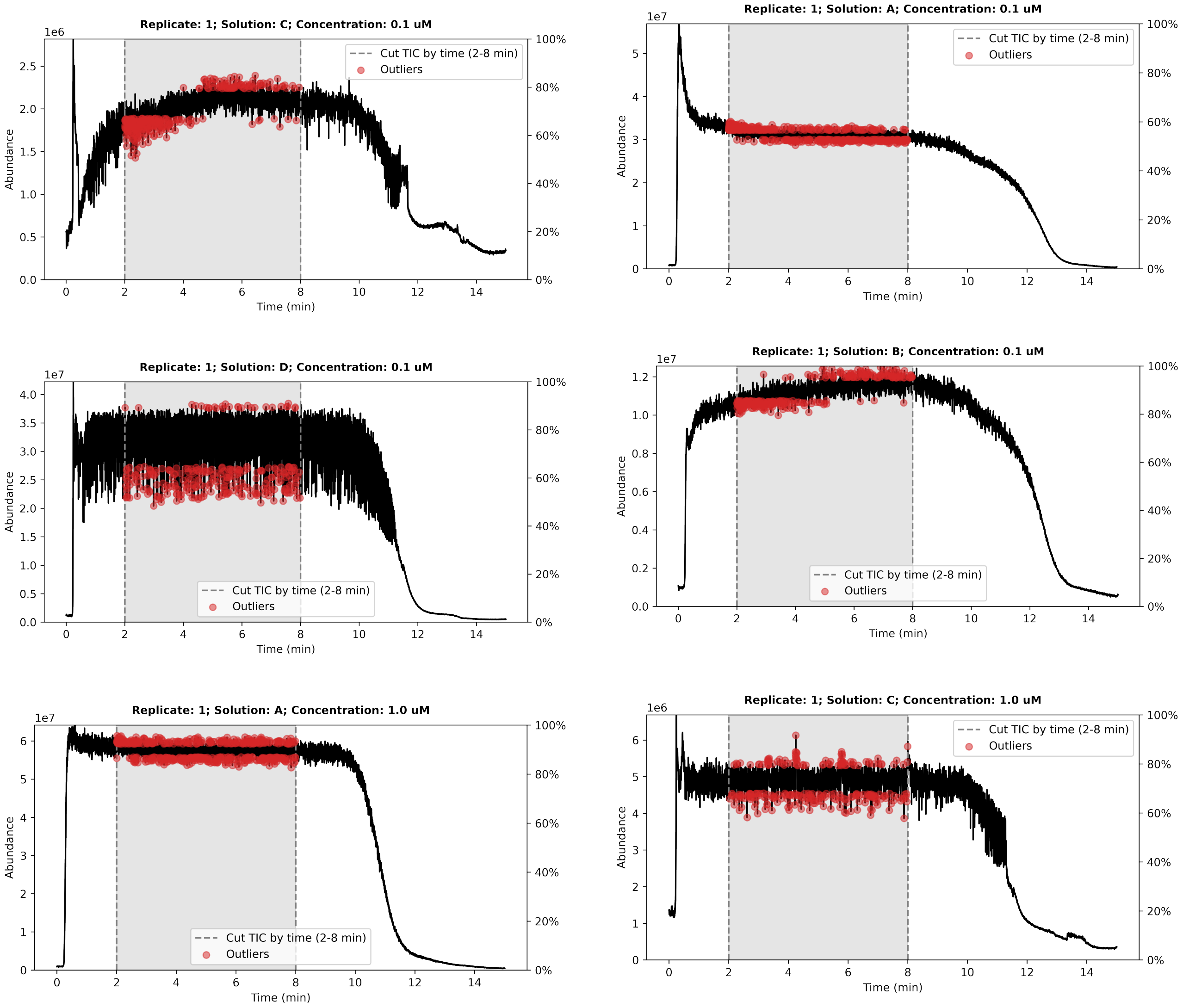

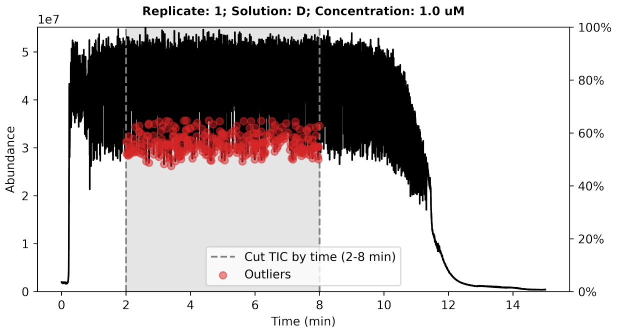
Replicate: 1; Solution: D; Concentration: 1.0 uM
1e7
Abundance
Time (min)
Cut TIC by time (2-8 min)
Outliers

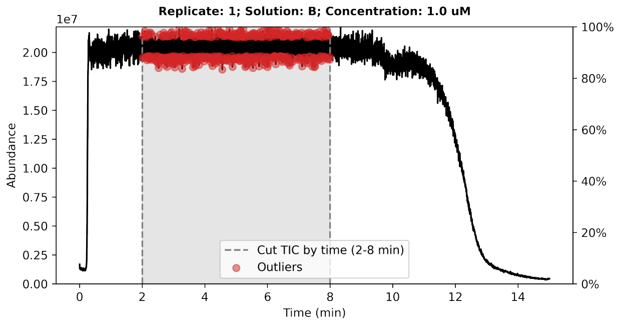
Replicate: 1; Solution: B; Concentration: 1.0 uM
1e7
Abundance
Time (min)
Cut TIC by time (2-8 min)
Outliers

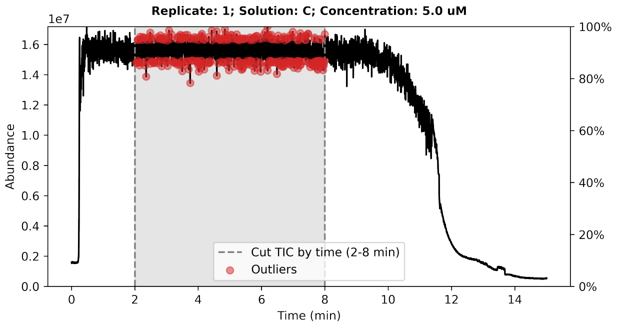
Replicate: 1; Solution: C; Concentration: 5.0 uM
1e7
Abundance
Time (min)
Cut TIC by time (2-8 min)
Outliers

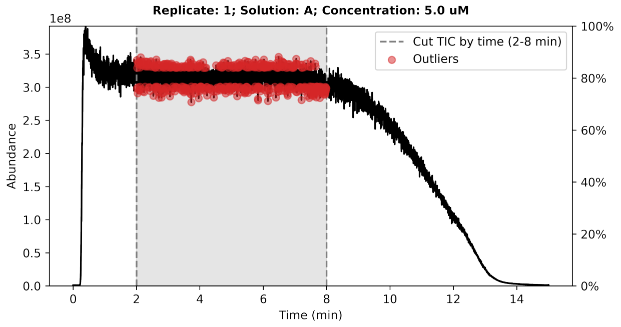
Replicate: 1; Solution: A; Concentration: 5.0 uM
1e8
Abundance
Time (min)
Cut TIC by time (2-8 min)
Outliers

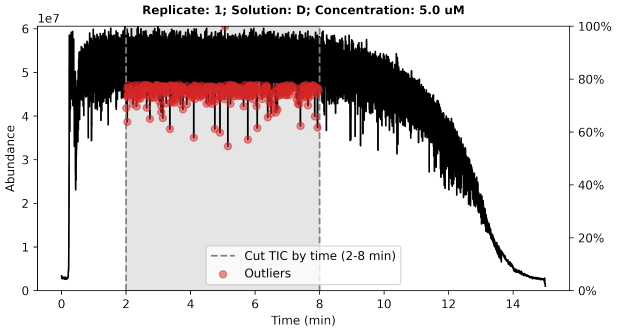
Replicate: 1; Solution: D; Concentration: 5.0 uM
1e7
Abundance
Time (min)
Cut TIC by time (2-8 min)
Outliers

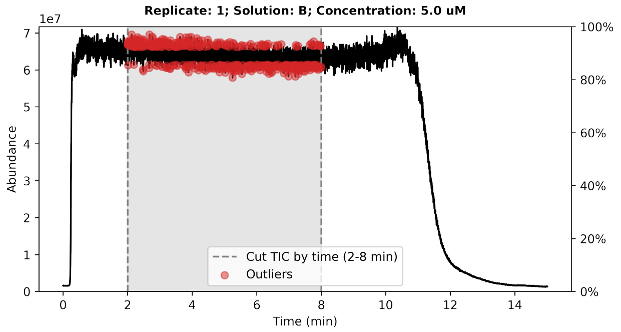
Replicate: 1; Solution: B; Concentration: 5.0 uM
1e7
Abundance
Time (min)
Cut TIC by time (2-8 min)
Outliers

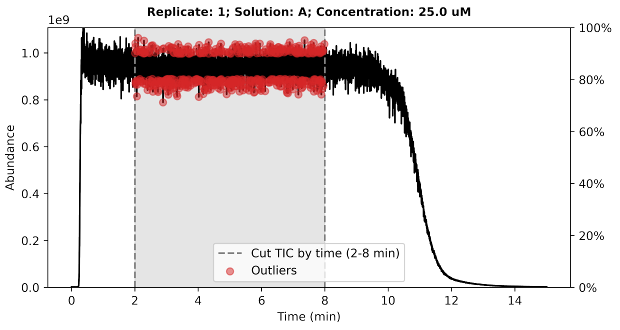
Replicate: 1; Solution: A; Concentration: 25.0 uM
1e9
Abundance
Time (min)
Cut TIC by time (2-8 min)
Outliers

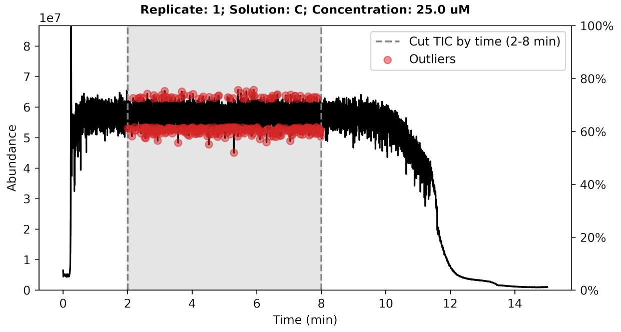
Replicate: 1; Solution: C; Concentration: 25.0 uM
1e7
Abundance
Time (min)
Cut TIC by time (2-8 min)
Outliers

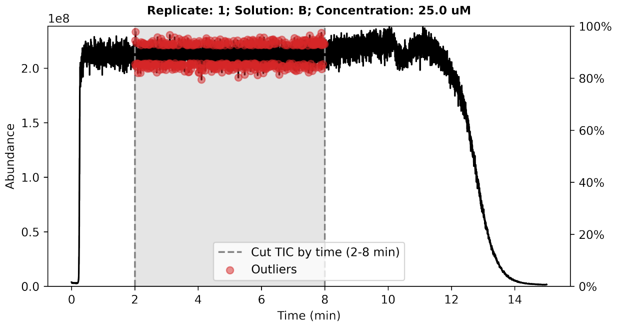
Replicate: 1; Solution: B; Concentration: 25.0 uM
1e8
Abundance
Time (min)
Cut TIC by time (2-8 min)
Outliers

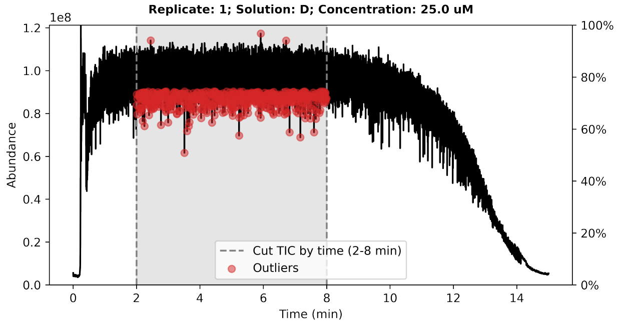
Replicate: 1; Solution: D; Concentration: 25.0 uM
1e8
Abundance
Time (min)
Cut TIC by time (2-8 min)
Outliers

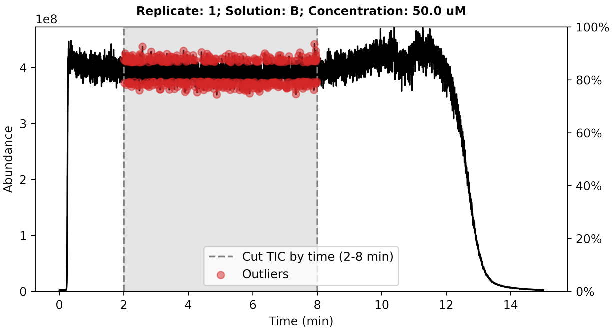
Replicate: 1; Solution: B; Concentration: 50.0 uM
1e8
Abundance
Time (min)
Cut TIC by time (2-8 min)
Outliers

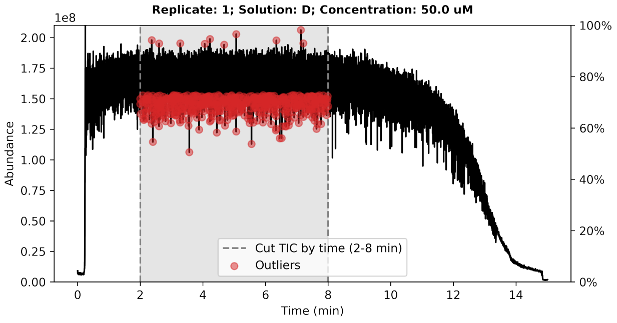
Replicate: 1; Solution: D; Concentration: 50.0 uM
1e8
Abundance
Time (min)
Cut TIC by time (2-8 min)
Outliers

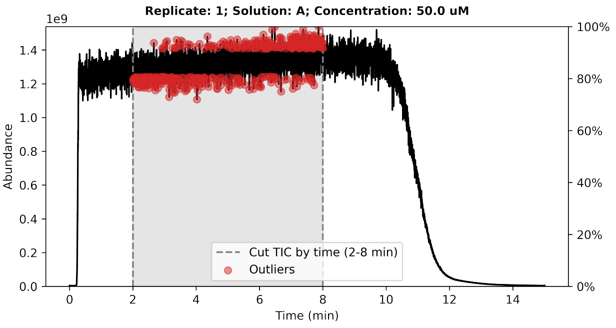
Replicate: 1; Solution: A; Concentration: 50.0 uM
1e9
Abundance
Time (min)
Cut TIC by time (2-8 min)
Outliers

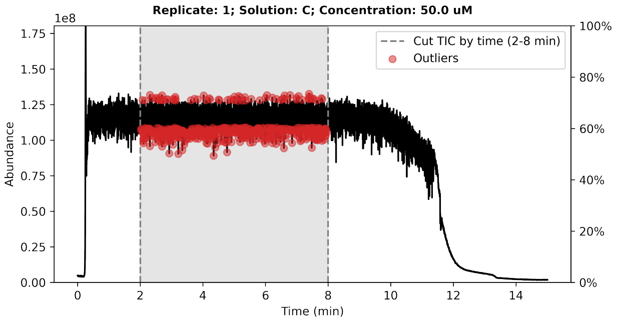
Replicate: 1; Solution: C; Concentration: 50.0 uM
1e8
Abundance
Time (min)
Cut TIC by time (2-8 min)
Outliers

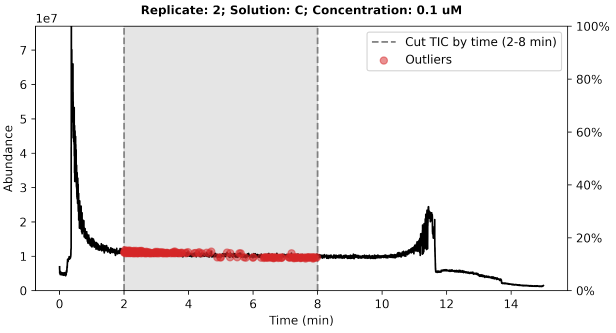
Replicate: 2; Solution: C; Concentration: 0.1 uM
1e7
Abundance
Time (min)
Cut TIC by time (2-8 min)
Outliers

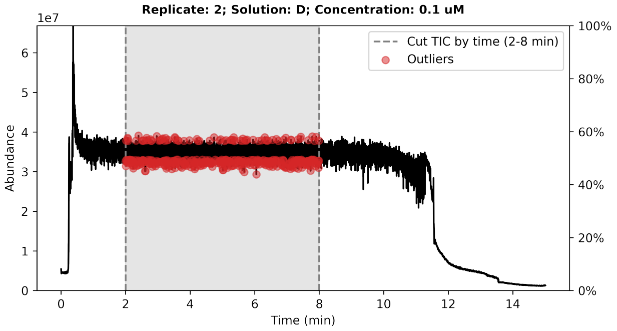
Replicate: 2; Solution: D; Concentration: 0.1 uM
1e7
Abundance
Time (min)
Cut TIC by time (2-8 min)
Outliers

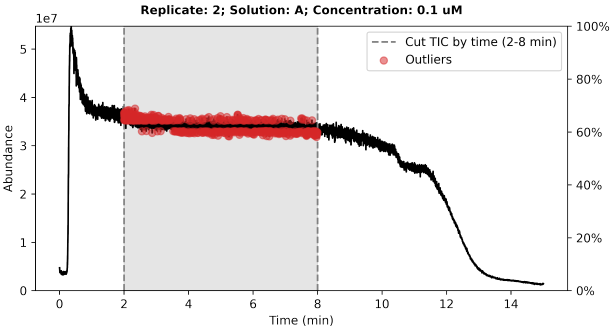
Replicate: 2; Solution: A; Concentration: 0.1 uM
1e7
Abundance
Time (min)
Cut TIC by time (2-8 min)
Outliers

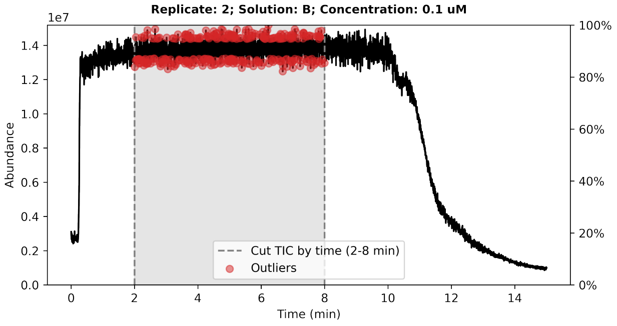
Replicate: 2; Solution: B; Concentration: 0.1 uM
1e7
Abundance
Time (min)
Cut TIC by time (2-8 min)
Outliers

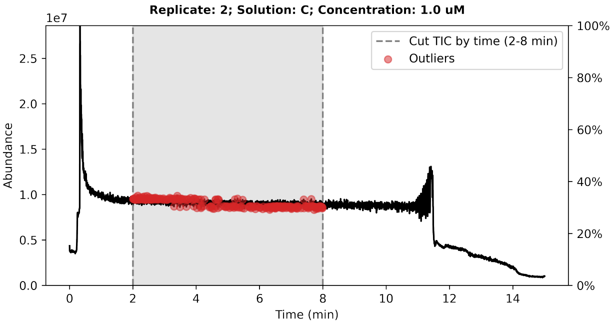
Replicate: 2; Solution: C; Concentration: 1.0 uM
1e7
Abundance
Time (min)
Cut TIC by time (2-8 min)
Outliers

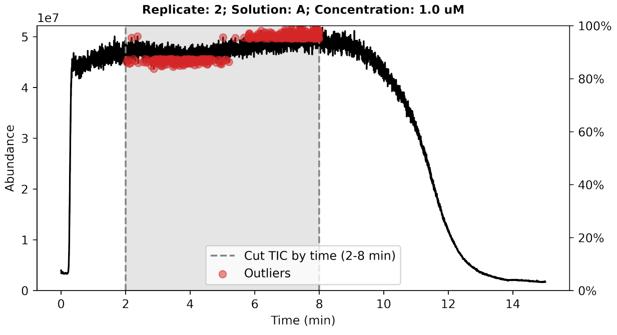
Replicate: 2; Solution: A; Concentration: 1.0 uM
1e7
Abundance
Time (min)
Cut TIC by time (2-8 min)
Outliers

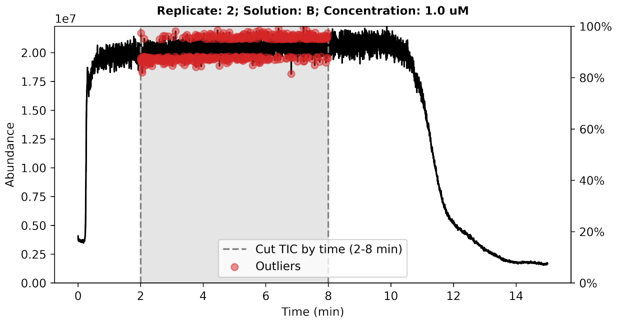
Replicate: 2; Solution: B; Concentration: 1.0 uM
1e7
Abundance
Time (min)
Cut TIC by time (2-8 min)
Outliers

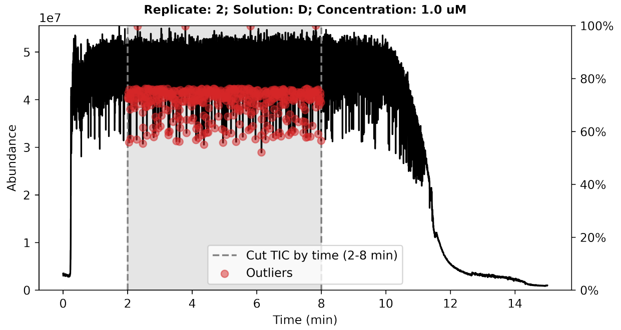
Replicate: 2; Solution: D; Concentration: 1.0 uM
1e7
Abundance
Time (min)
Cut TIC by time (2-8 min)
Outliers

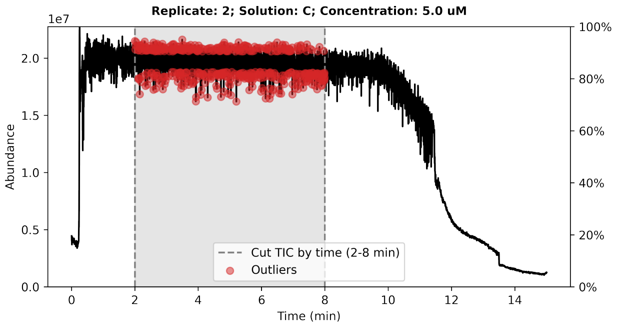
Replicate: 2; Solution: C; Concentration: 5.0 uM
1e7
Abundance
Time (min)
Cut TIC by time (2-8 min)
Outliers

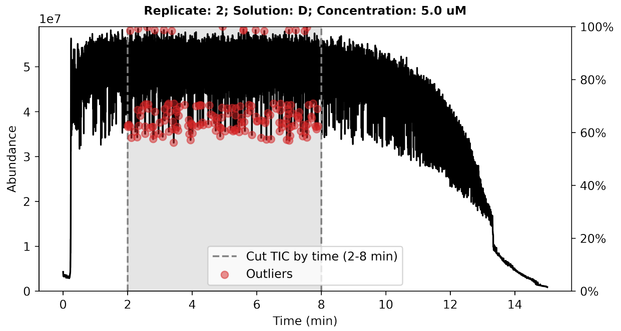
Replicate: 2; Solution: D; Concentration: 5.0 uM
1e7
Abundance
Time (min)
Cut TIC by time (2-8 min)
Outliers

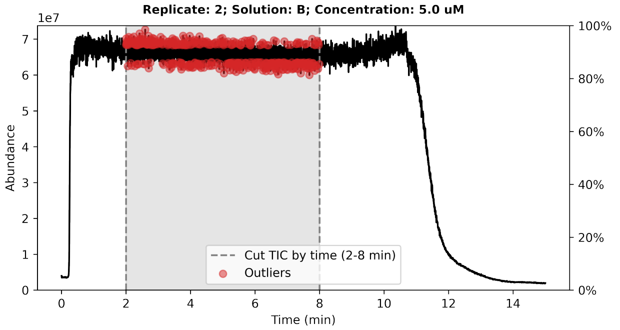
Replicate: 2; Solution: B; Concentration: 5.0 uM
1e7
Abundance
Time (min)
Cut TIC by time (2-8 min)
Outliers

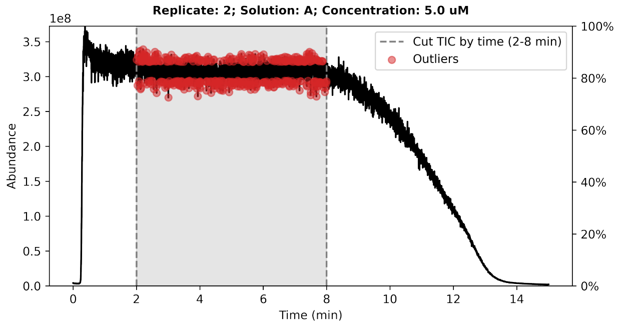
Replicate: 2; Solution: A; Concentration: 5.0 uM
1e8
Abundance
Time (min)
Cut TIC by time (2-8 min)
Outliers

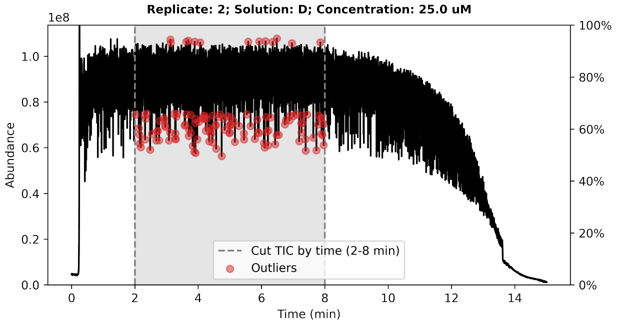
Replicate: 2; Solution: D; Concentration: 25.0 uM
1e8
Abundance
Time (min)
Cut TIC by time (2-8 min)
Outliers

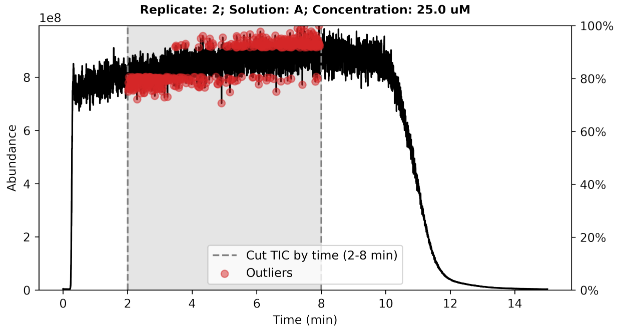
Replicate: 2; Solution: A; Concentration: 25.0 uM
1e8
Abundance
Time (min)
Cut TIC by time (2-8 min)
Outliers

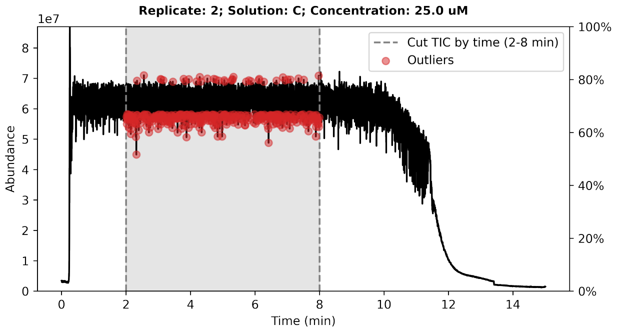
Replicate: 2; Solution: C; Concentration: 25.0 uM
1e7
Abundance
Time (min)
Cut TIC by time (2-8 min)
Outliers

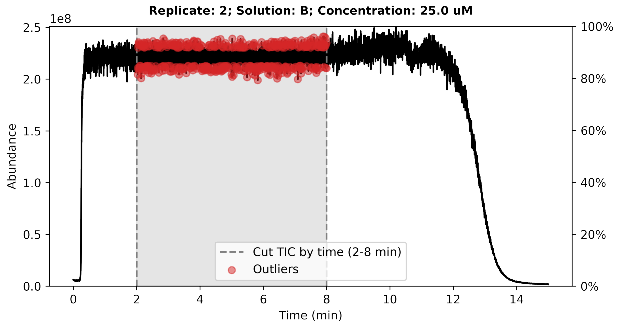
Replicate: 2; Solution: B; Concentration: 25.0 uM
1e8
Abundance
Time (min)
Cut TIC by time (2-8 min)
Outliers

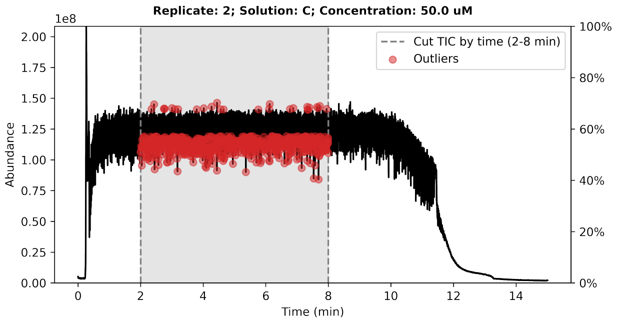
Replicate: 2; Solution: C; Concentration: 50.0 uM
1e8
Cut TIC by time (2-8 min)
Outliers
Abundance
Time (min)

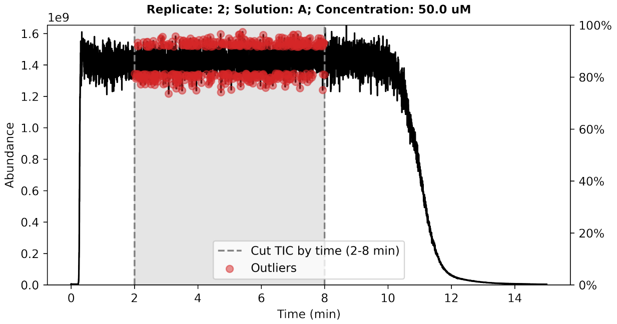
Replicate: 2; Solution: A; Concentration: 50.0 uM
1e9
Cut TIC by time (2-8 min)
Outliers
Abundance
Time (min)

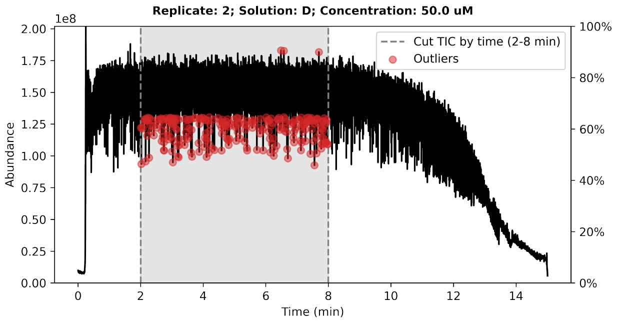
Replicate: 2; Solution: D; Concentration: 50.0 uM
1e8
Cut TIC by time (2-8 min)
Outliers
Abundance
Time (min)

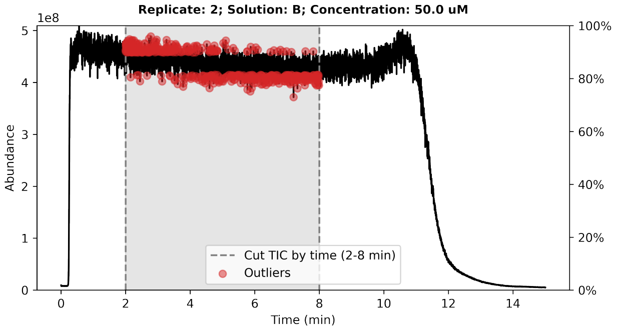
Replicate: 2; Solution: B; Concentration: 50.0 uM
1e8
Cut TIC by time (2-8 min)
Outliers
Abundance
Time (min)

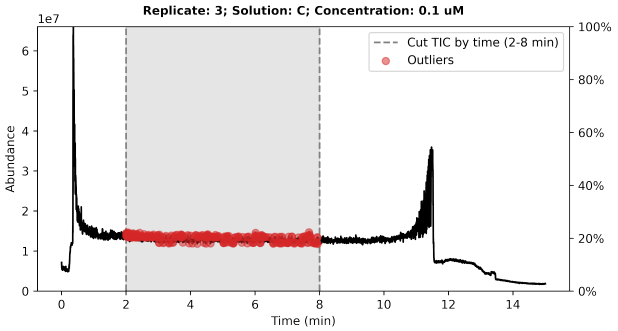
Replicate: 3; Solution: C; Concentration: 0.1 uM
1e7
Cut TIC by time (2-8 min)
Outliers
Abundance
Time (min)

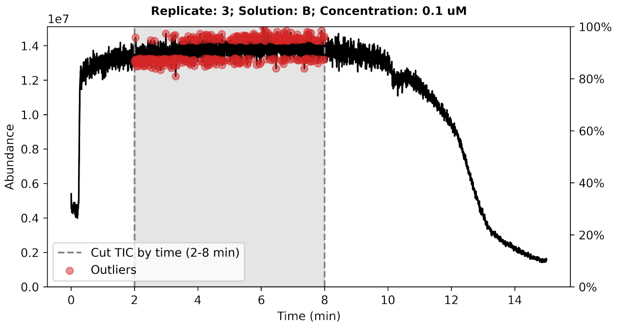
Replicate: 3; Solution: B; Concentration: 0.1 uM
1e7
Cut TIC by time (2-8 min)
Outliers
Abundance
Time (min)

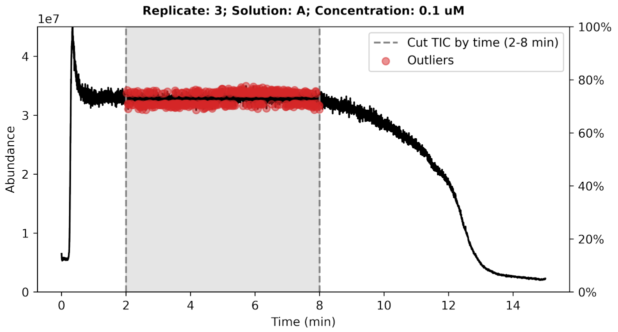
Replicate: 3; Solution: A; Concentration: 0.1 uM
1e7
Cut TIC by time (2-8 min)
Outliers
Abundance
Time (min)

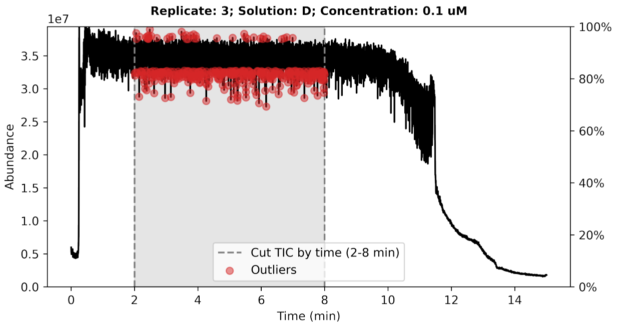
Replicate: 3; Solution: D; Concentration: 0.1 uM
1e7
Cut TIC by time (2-8 min)
Outliers
Abundance
Time (min)

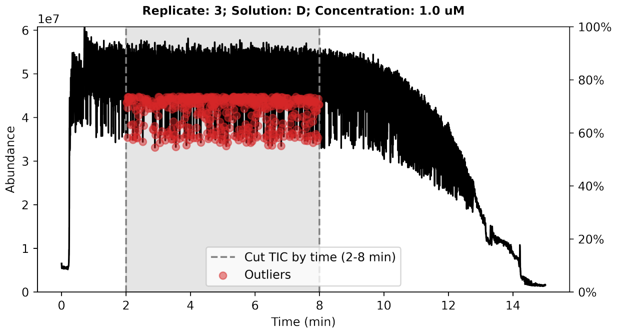
Replicate: 3; Solution: D; Concentration: 1.0 uM
1e7
Cut TIC by time (2-8 min)
Outliers
Abundance
Time (min)

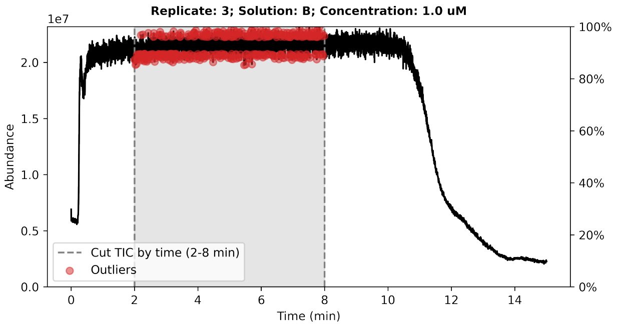
Replicate: 3; Solution: B; Concentration: 1.0 uM
1e7
Cut TIC by time (2-8 min)
Outliers
Abundance
Time (min)

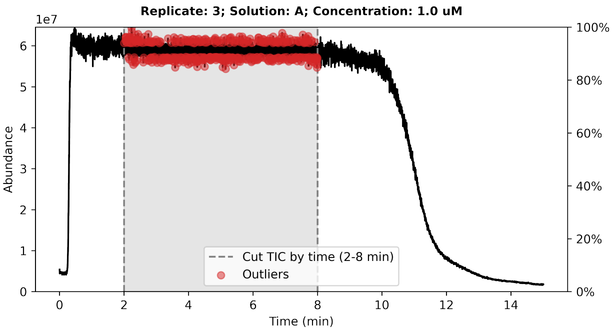
Replicate: 3; Solution: A; Concentration: 1.0 uM
1e7
Abundance
Time (min)
Cut TIC by time (2-8 min)
Outliers

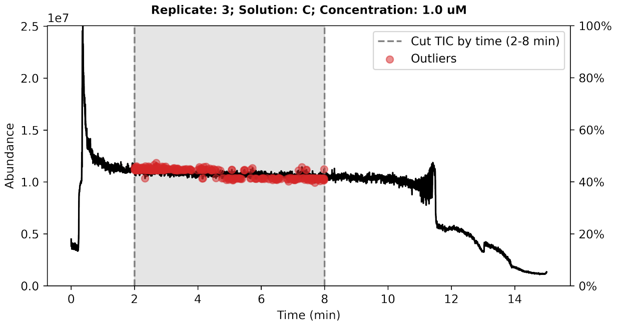
Replicate: 3; Solution: C; Concentration: 1.0 uM
1e7
Abundance
Time (min)
Cut TIC by time (2-8 min)
Outliers

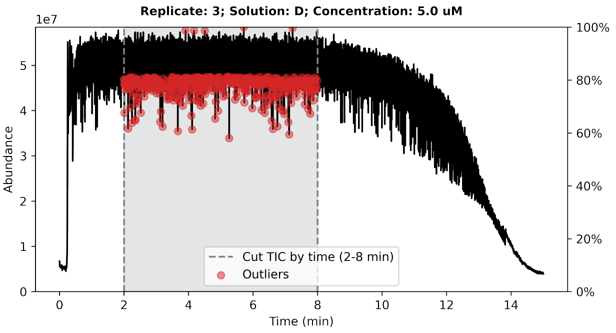
Replicate: 3; Solution: D; Concentration: 5.0 uM
1e7
Abundance
Time (min)
Cut TIC by time (2-8 min)
Outliers

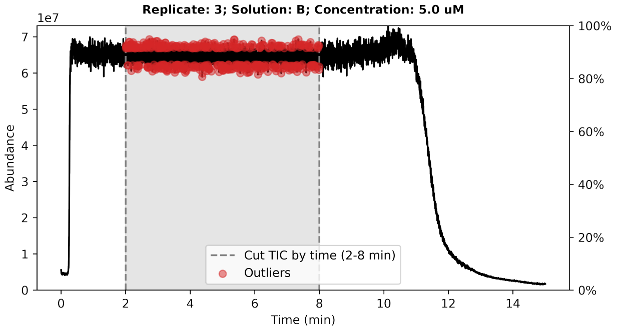
Replicate: 3; Solution: B; Concentration: 5.0 uM
1e7
Abundance
Time (min)
Cut TIC by time (2-8 min)
Outliers

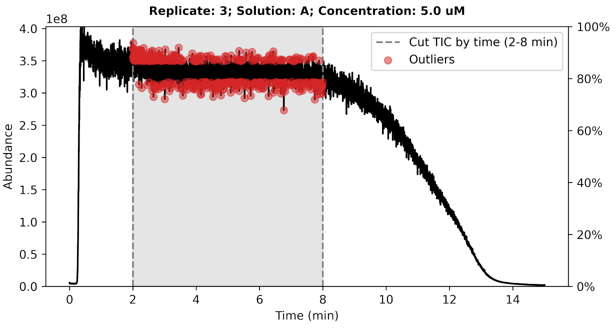
Replicate: 3; Solution: A; Concentration: 5.0 uM
1e8
Abundance
Time (min)
Cut TIC by time (2-8 min)
Outliers

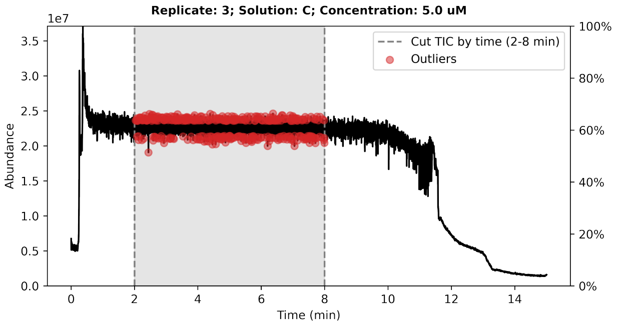
Replicate: 3; Solution: C; Concentration: 5.0 uM
1e7
Abundance
Time (min)
Cut TIC by time (2-8 min)
Outliers

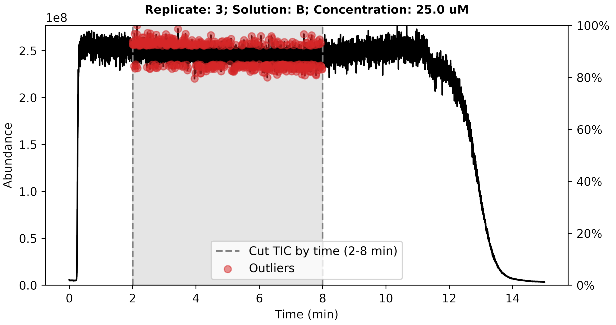
Replicate: 3; Solution: B; Concentration: 25.0 uM
1e8
Abundance
Time (min)
Cut TIC by time (2-8 min)
Outliers

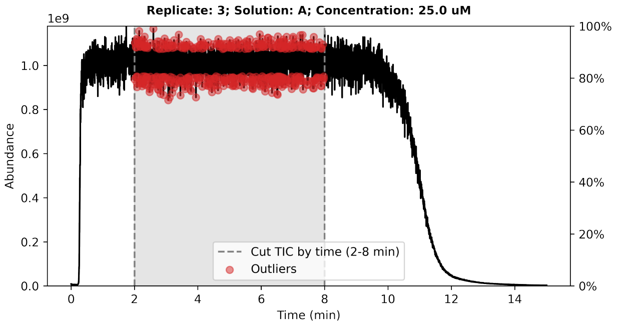
Replicate: 3; Solution: A; Concentration: 25.0 uM
1e9
Abundance
Time (min)
Cut TIC by time (2-8 min)
Outliers

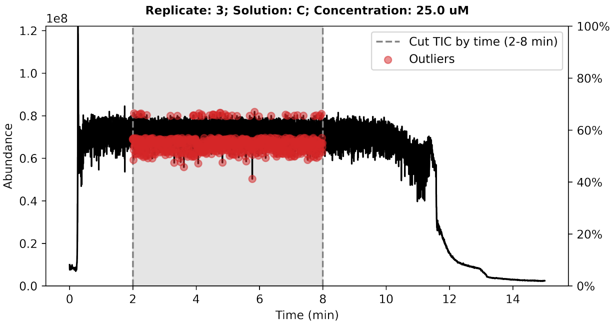
Replicate: 3; Solution: C; Concentration: 25.0 uM
1e8
Abundance
Time (min)
Cut TIC by time (2-8 min)
Outliers

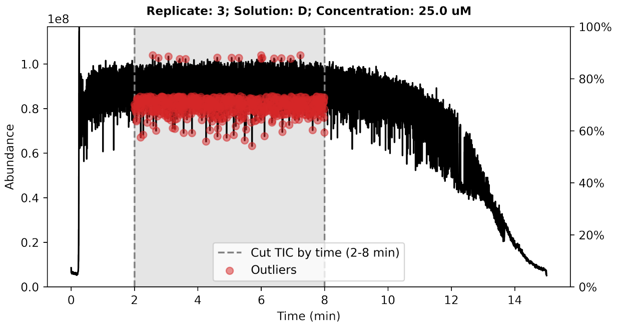
Replicate: 3; Solution: D; Concentration: 25.0 uM
1e8
Abundance
Time (min)
Cut TIC by time (2-8 min)
Outliers

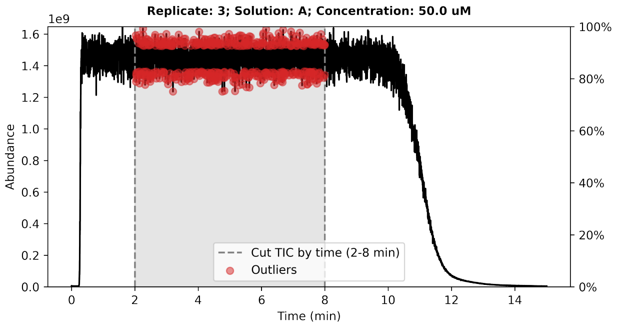
Replicate: 3; Solution: A; Concentration: 50.0 uM
1e9
Abundance
Time (min)
Cut TIC by time (2-8 min)
Outliers

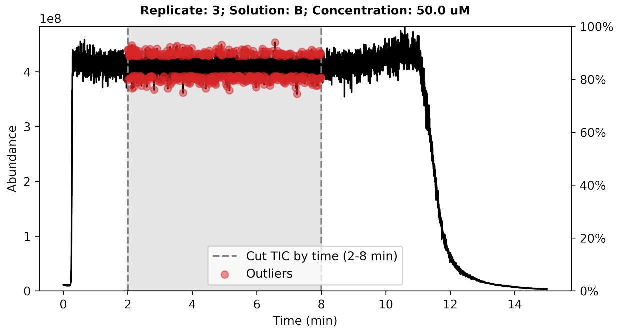
Replicate: 3; Solution: B; Concentration: 50.0 uM
1e8
Abundance
Time (min)
Cut TIC by time (2-8 min)
Outliers

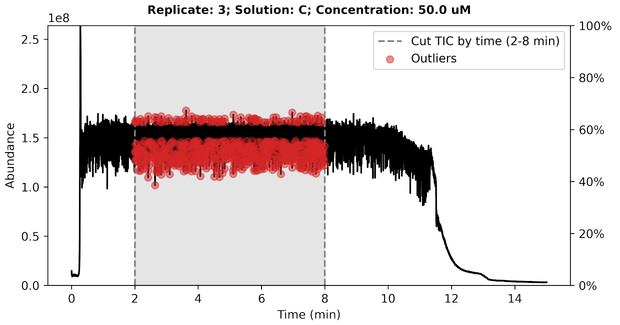
Replicate: 3; Solution: C; Concentration: 50.0 uM
1e8
Abundance
Time (min)
Cut TIC by time (2-8 min)
Outliers

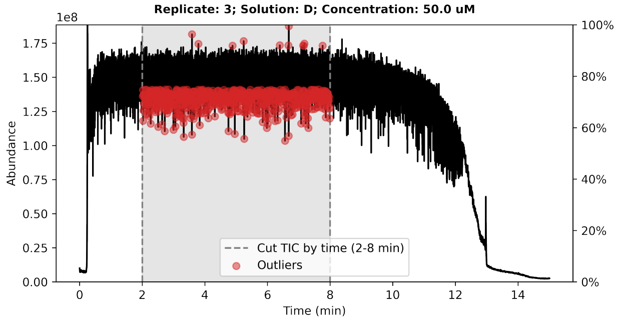
Replicate: 3; Solution: D; Concentration: 50.0 uM
1e8
Abundance
Time (min)
Cut TIC by time (2-8 min)
Outliers

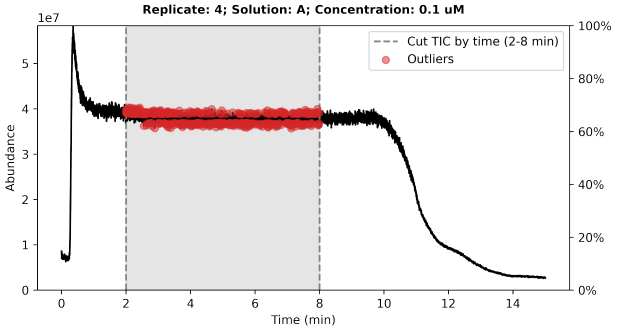
Replicate: 4; Solution: A; Concentration: 0.1 uM
1e7
Abundance
Time (min)
Cut TIC by time (2-8 min)
Outliers

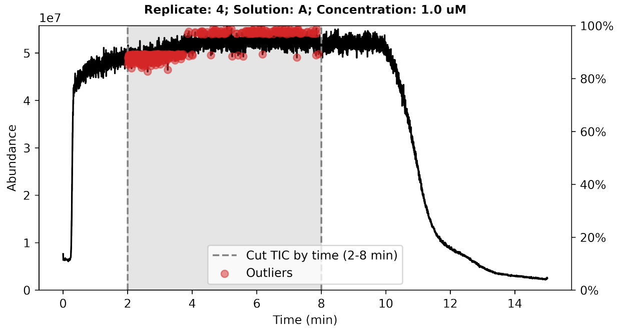
Replicate: 4; Solution: A; Concentration: 1.0 uM
1e7
Abundance
Time (min)
Cut TIC by time (2-8 min)
Outliers

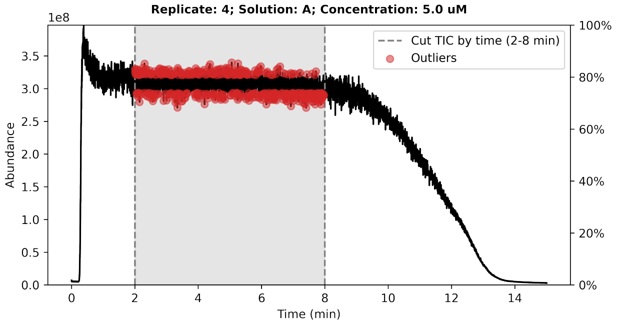
Replicate: 4; Solution: A; Concentration: 5.0 uM
1e8
Abundance
Time (min)
Cut TIC by time (2-8 min)
Outliers

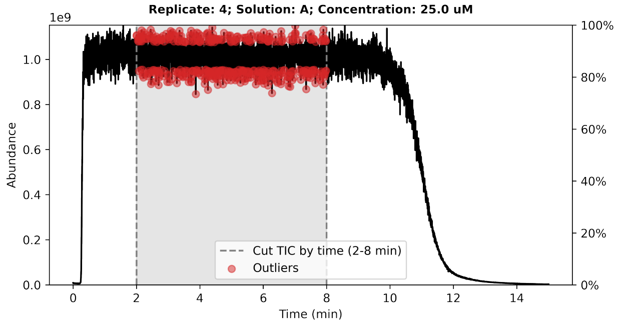
Replicate: 4; Solution: A; Concentration: 25.0 uM
1e9
Abundance
Time (min)
Cut TIC by time (2-8 min)
Outliers

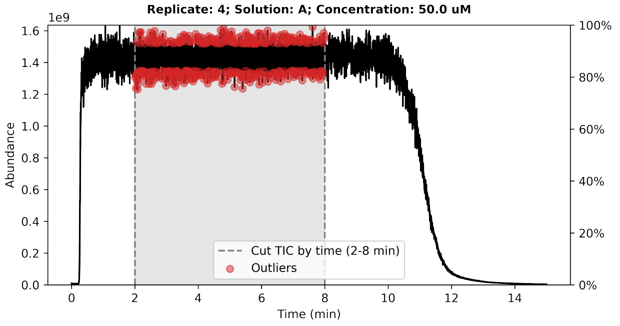
Replicate: 4; Solution: A; Concentration: 50.0 uM
1e9
Abundance
Time (min)
Cut TIC by time (2-8 min)
Outliers

3. Isotope Ratio Profile

The Isotopic Ratio were calculated by 'Mean'

Replicate-Solution-Concentration	Effective number of ions	Isotopic Ratio	Acquisition Error (permil)	Shot-Noise (permil)	AE/SN ratio
1-C-0.1	9.80e+05	0.114576	1.410	1.010	1.396
1-A-0.1	3.97e+07	0.120746	0.176	0.159	1.108
1-D-0.1	4.41e+07	0.121424	0.163	0.151	1.085
1-B-0.1	9.32e+05	0.118333	1.190	1.036	1.149
1-A-1.0	3.97e+07	0.120972	0.168	0.159	1.059
1-C-1.0	1.20e+07	0.120239	0.374	0.289	1.296
1-D-1.0	4.33e+07	0.121482	0.165	0.152	1.083
1-B-1.0	2.22e+07	0.120907	0.288	0.212	1.358
1-C-5.0	3.17e+07	0.121435	0.199	0.178	1.120
1-A-5.0	3.97e+07	0.121518	0.169	0.159	1.062
1-D-5.0	4.21e+07	0.121490	0.170	0.154	1.100
1-B-5.0	4.03e+07	0.121343	0.184	0.157	1.166
1-A-25.0	3.99e+07	0.121196	0.171	0.158	1.077
1-C-25.0	4.35e+07	0.121453	0.167	0.152	1.101
1-B-25.0	4.04e+07	0.121250	0.175	0.157	1.113
1-D-25.0	3.93e+07	0.121404	0.175	0.160	1.098
1-B-50.0	4.11e+07	0.121250	0.170	0.156	1.092
1-D-50.0	3.90e+07	0.121463	0.171	0.160	1.070
1-A-50.0	4.06e+07	0.120975	0.174	0.157	1.110
1-C-50.0	4.02e+07	0.121514	0.176	0.158	1.114
2-C-0.1	2.23e+07	0.121156	0.259	0.212	1.224
2-D-0.1	4.10e+07	0.121530	0.168	0.156	1.079
2-A-0.1	3.85e+07	0.120827	0.176	0.161	1.090
2-B-0.1	2.95e+06	0.119209	0.666	0.582	1.143
2-C-1.0	2.04e+07	0.121141	0.265	0.221	1.197
2-A-1.0	4.10e+07	0.121050	0.168	0.156	1.074
2-B-1.0	3.03e+07	0.121028	0.242	0.182	1.331
2-D-1.0	3.98e+07	0.121456	0.173	0.159	1.089

Replicate-Solution-Concentration	Effective number of ions	Isotopic Ratio	Acquisition Error (permil)	Shot-Noise (permil)	AE/SN ratio
2-C-5.0	3.59e+07	0.121226	0.193	0.167	1.156
2-D-5.0	4.56e+07	0.121431	0.159	0.148	1.075
2-B-5.0	3.98e+07	0.121087	0.187	0.158	1.179
2-A-5.0	3.98e+07	0.121508	0.169	0.159	1.069
2-D-25.0	4.61e+07	0.121440	0.158	0.147	1.073
2-A-25.0	3.96e+07	0.121207	0.171	0.159	1.076
2-C-25.0	4.17e+07	0.121447	0.167	0.155	1.076
2-B-25.0	4.03e+07	0.121336	0.174	0.158	1.107
2-C-50.0	3.64e+07	0.121542	0.179	0.166	1.081
2-A-50.0	4.04e+07	0.120900	0.170	0.157	1.078
2-D-50.0	4.50e+07	0.121485	0.163	0.149	1.095
2-B-50.0	4.02e+07	0.121242	0.170	0.158	1.075
3-C-0.1	2.52e+07	0.121140	0.228	0.199	1.143
3-B-0.1	4.83e+06	0.119574	0.558	0.455	1.227
3-A-0.1	3.87e+07	0.120483	0.177	0.161	1.101
3-D-0.1	4.04e+07	0.121197	0.173	0.157	1.099
3-D-1.0	3.85e+07	0.121163	0.170	0.161	1.052
3-B-1.0	3.30e+07	0.120761	0.239	0.174	1.373
3-A-1.0	3.86e+07	0.120949	0.171	0.161	1.063
3-C-1.0	2.13e+07	0.120991	0.248	0.217	1.146
3-D-5.0	3.79e+07	0.121251	0.167	0.162	1.029
3-B-5.0	3.90e+07	0.120894	0.194	0.160	1.209
3-A-5.0	3.88e+07	0.121337	0.170	0.161	1.056
3-C-5.0	3.94e+07	0.121155	0.174	0.159	1.090
3-B-25.0	3.95e+07	0.121128	0.171	0.159	1.076
3-A-25.0	3.80e+07	0.120904	0.170	0.162	1.050
3-C-25.0	3.41e+07	0.121193	0.180	0.171	1.049
3-D-25.0	3.47e+07	0.121260	0.174	0.170	1.025
3-A-50.0	3.73e+07	0.120716	0.170	0.164	1.040
3-B-50.0	3.86e+07	0.121056	0.167	0.161	1.039
3-C-50.0	3.48e+07	0.121359	0.179	0.170	1.056
3-D-50.0	3.46e+07	0.121342	0.175	0.170	1.027

Replicate-Solution-Concentration	Effective number of ions	Isotopic Ratio	Acquisition Error (permil)	Shot-Noise (permil)	AE/SN ratio
4-A-0.1	3.71e+07	0.120773	0.170	0.164	1.034
4-A-1.0	3.73e+07	0.120810	0.174	0.164	1.065
4-A-5.0	3.74e+07	0.121290	0.170	0.163	1.038
4-A-25.0	3.82e+07	0.121089	0.169	0.162	1.042
4-A-50.0	3.81e+07	0.120773	0.168	0.162	1.036

Replicate: 1; Solution: C; Concentration: 0.1 uM

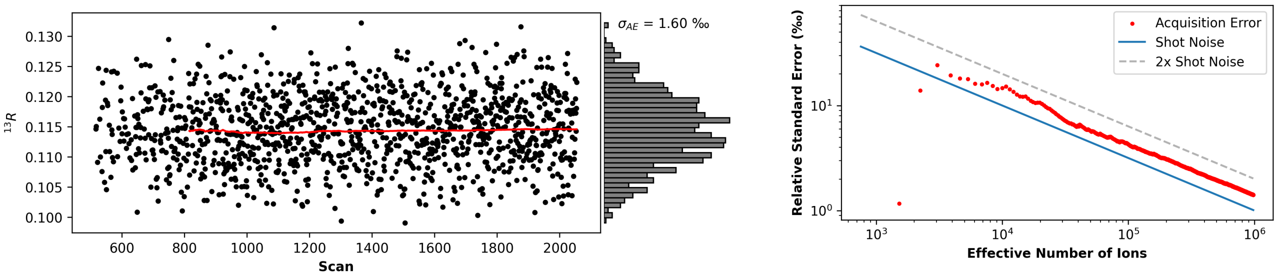

Replicate: 1; Solution: A; Concentration: 0.1 uM

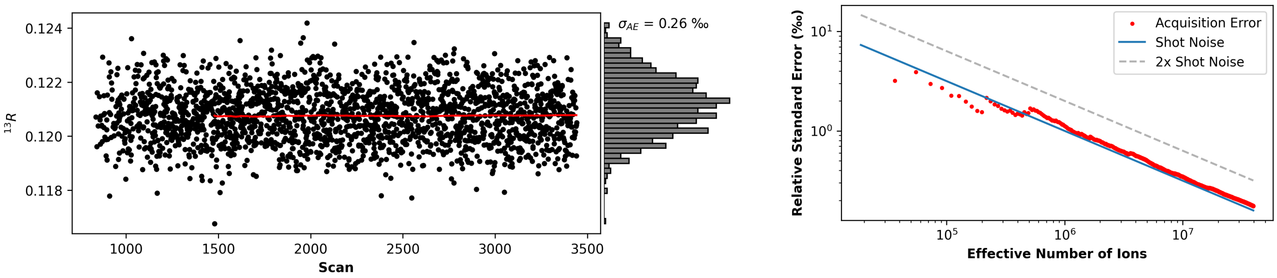

Replicate: 1; Solution: D; Concentration: 0.1 uM

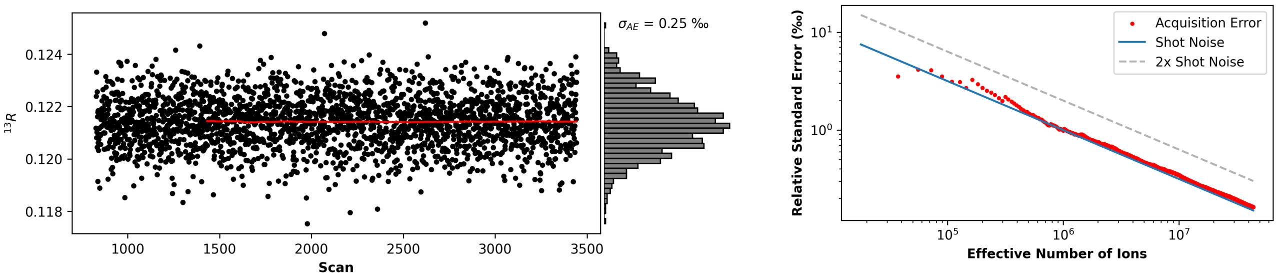

Replicate: 1; Solution: B; Concentration: 0.1 uM

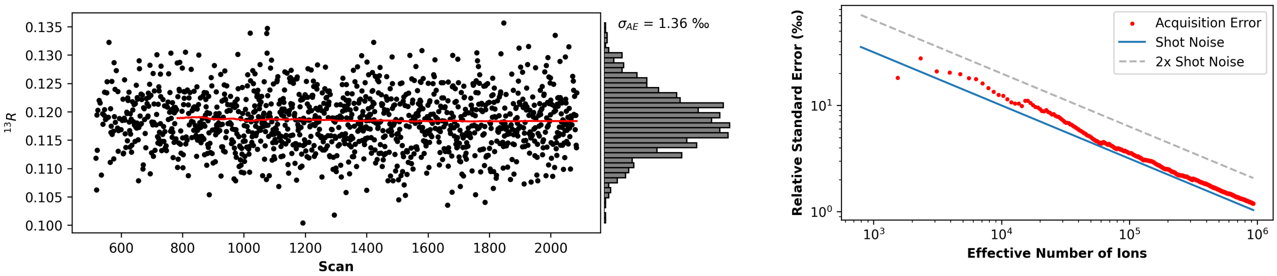

Replicate: 1; Solution: A; Concentration: 1.0 uM

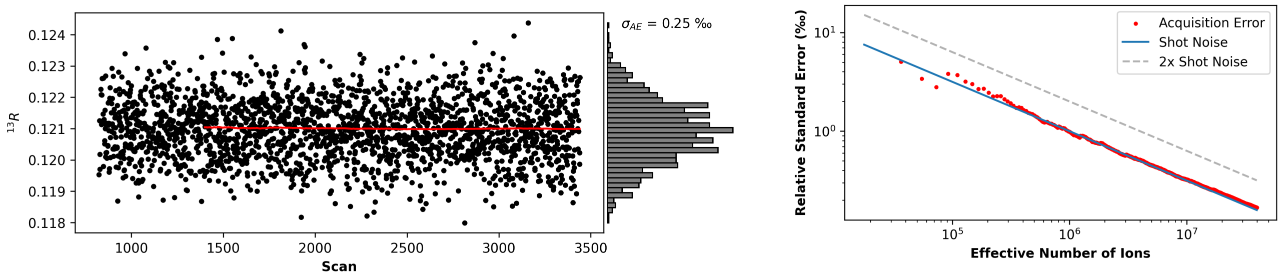

Replicate: 1; Solution: C; Concentration: 1.0 uM

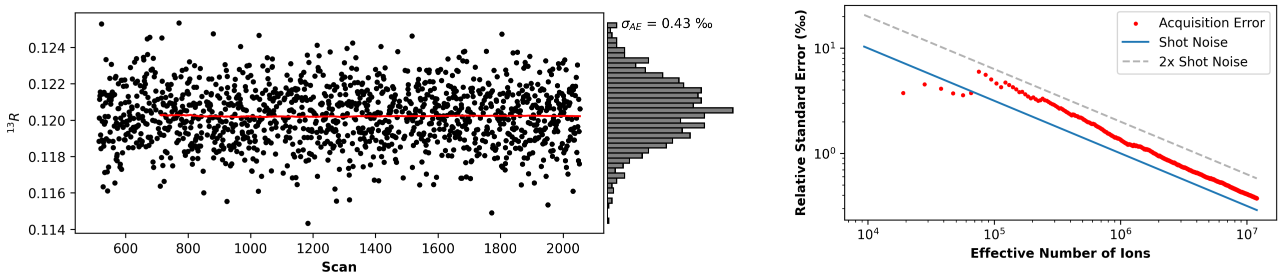

Replicate: 1; Solution: D; Concentration: 1.0 uM

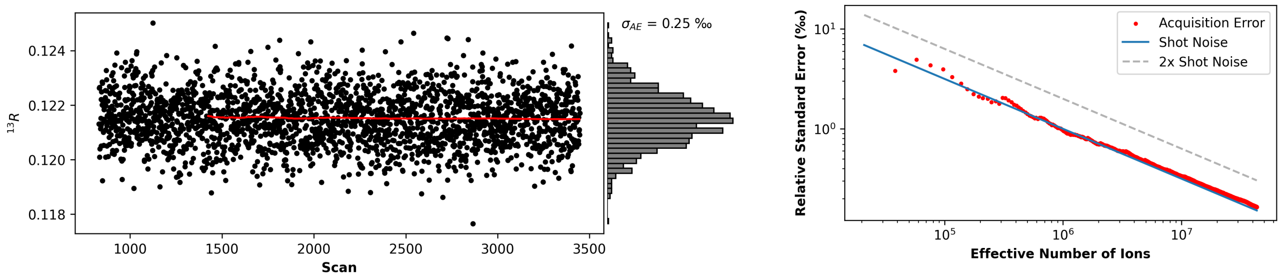

Replicate: 1; Solution: B; Concentration: 1.0 uM

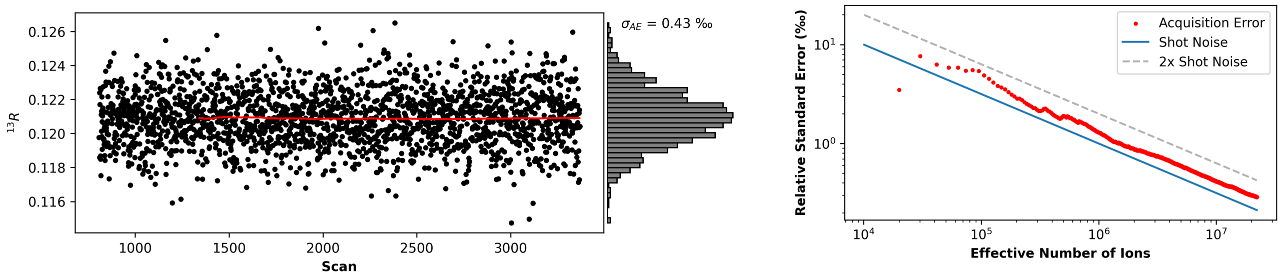

Replicate: 1; Solution: C; Concentration: 5.0 uM

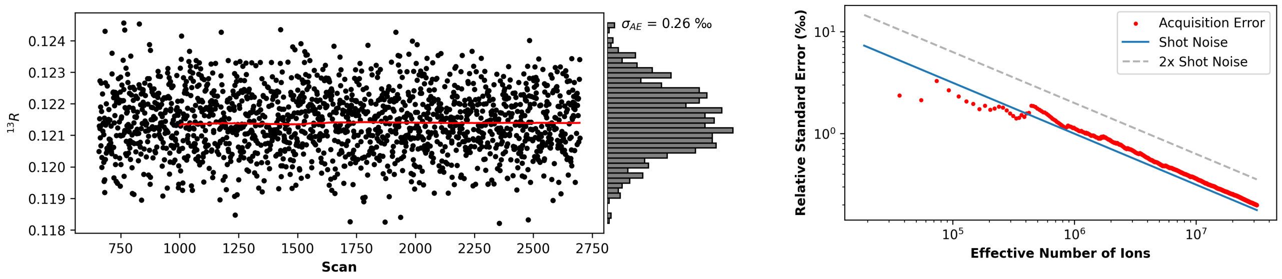

Replicate: 1; Solution: A; Concentration: 5.0 uM

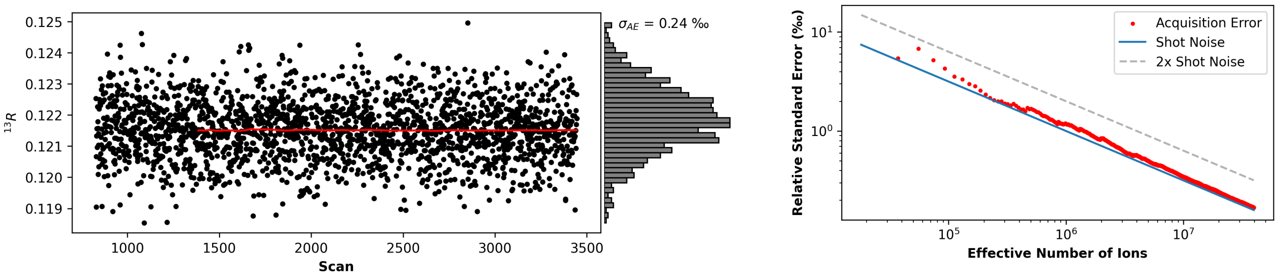

Replicate: 1; Solution: D; Concentration: 5.0 uM

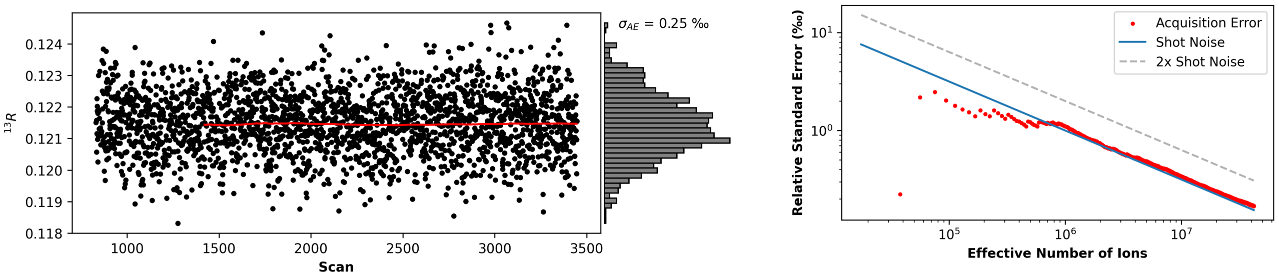

Replicate: 1; Solution: B; Concentration: 5.0 uM

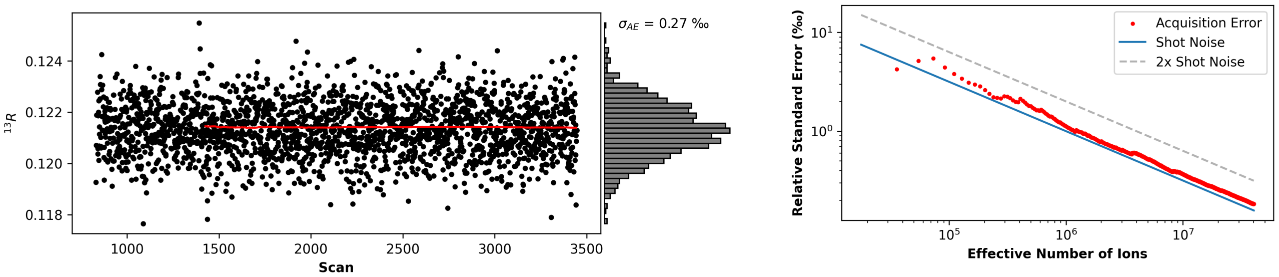

Replicate: 1; Solution: A; Concentration: 25.0 uM

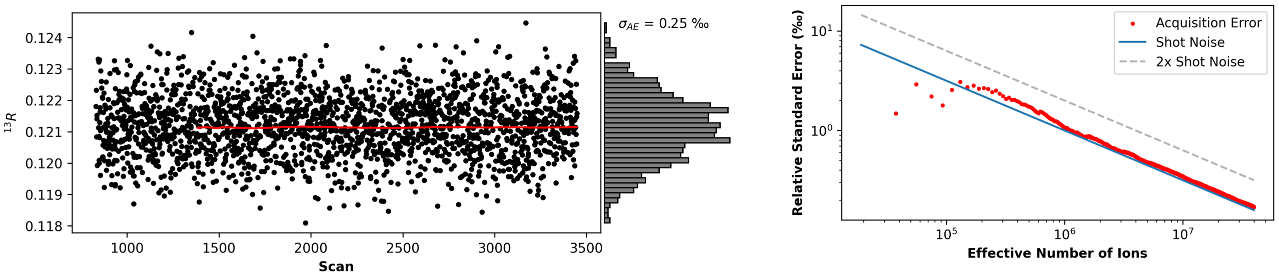

Replicate: 1; Solution: C; Concentration: 25.0 uM

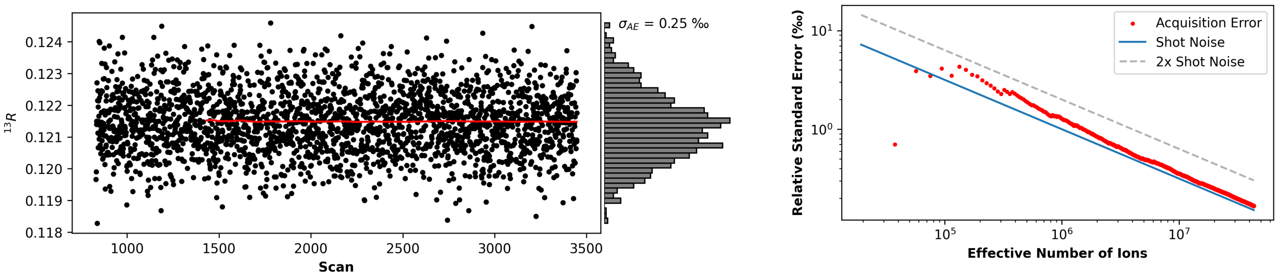

Replicate: 1; Solution: B; Concentration: 25.0 uM

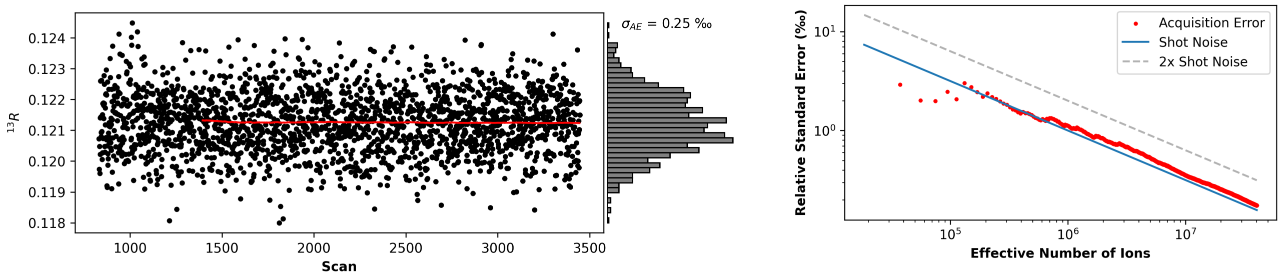

Replicate: 1; Solution: D; Concentration: 25.0 uM

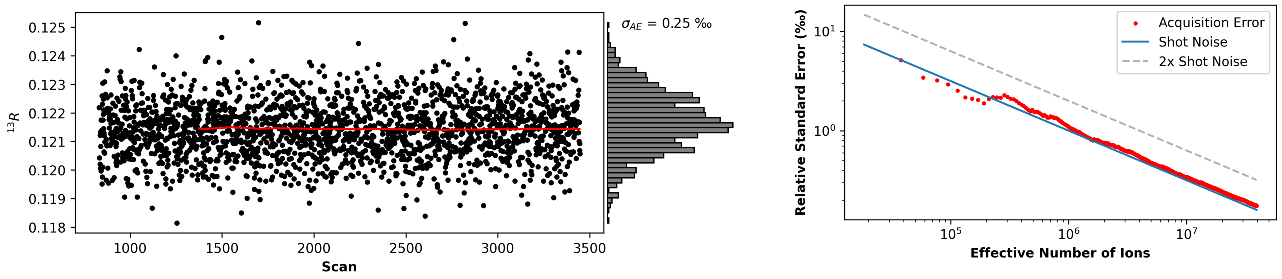

Replicate: 1; Solution: B; Concentration: 50.0 uM

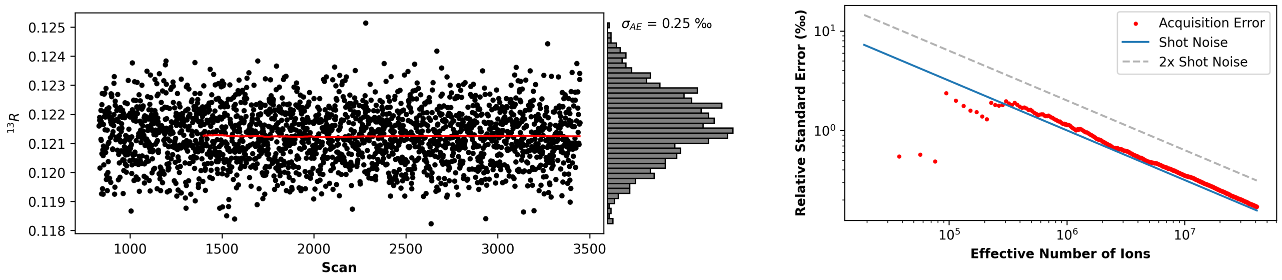

Replicate: 1; Solution: D; Concentration: 50.0 uM

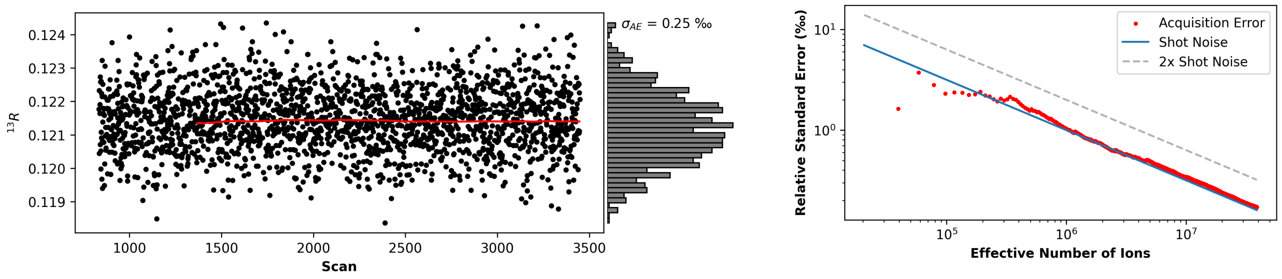

Replicate: 1; Solution: A; Concentration: 50.0 uM

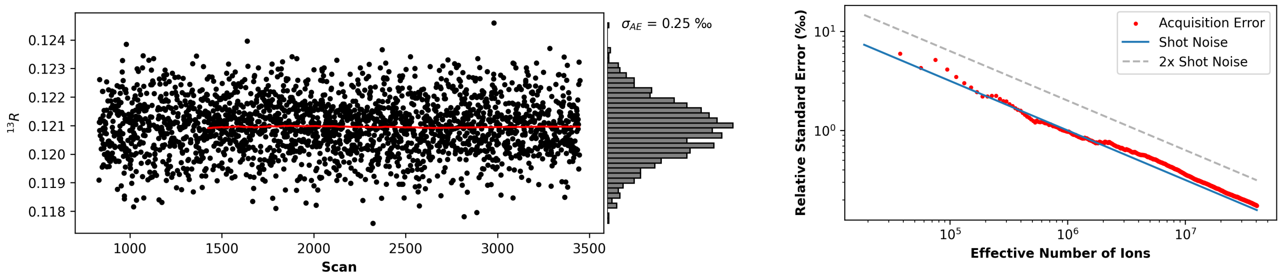

Replicate: 1; Solution: C; Concentration: 50.0 uM

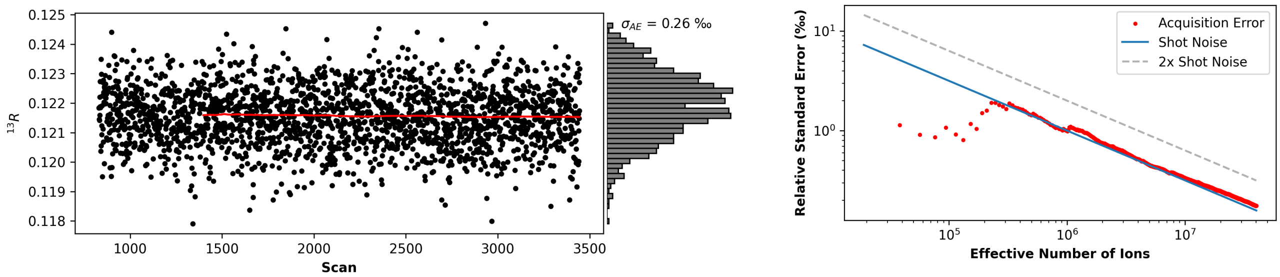

Replicate: 2; Solution: C; Concentration: 0.1 uM

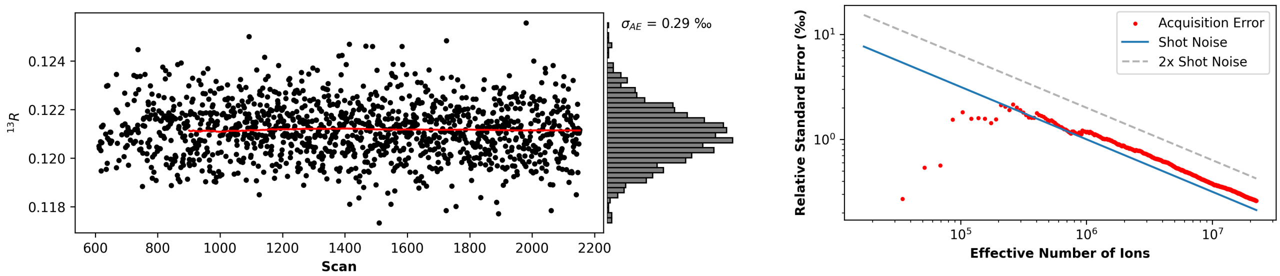

Replicate: 2; Solution: D; Concentration: 0.1 uM

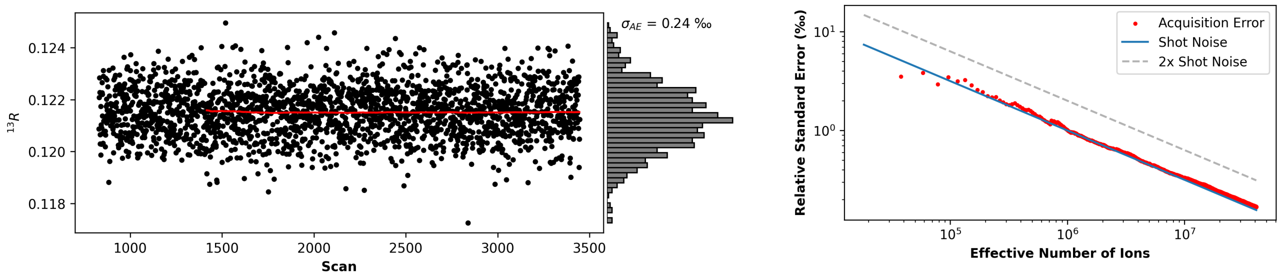

Replicate: 2; Solution: A; Concentration: 0.1 uM

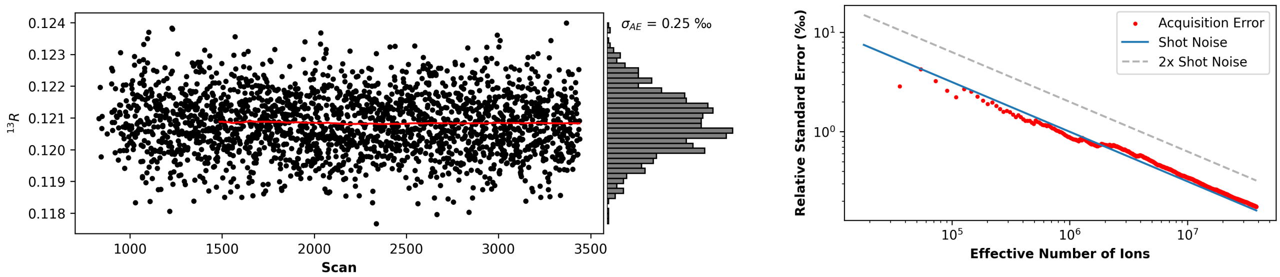

Replicate: 2; Solution: B; Concentration: 0.1 uM

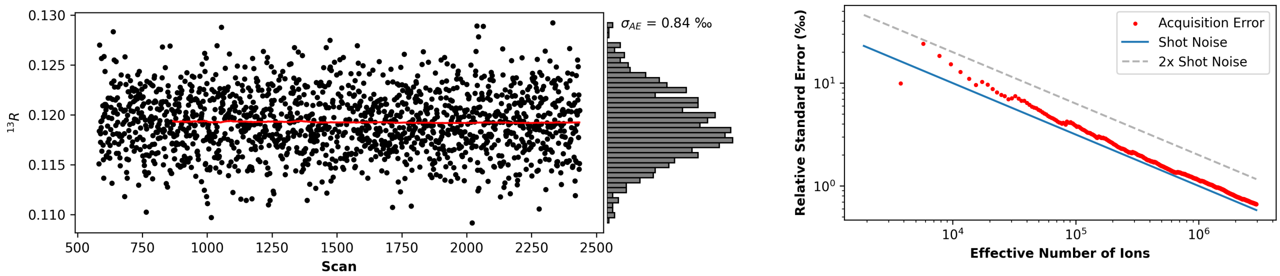

Replicate: 2; Solution: C; Concentration: 1.0 uM

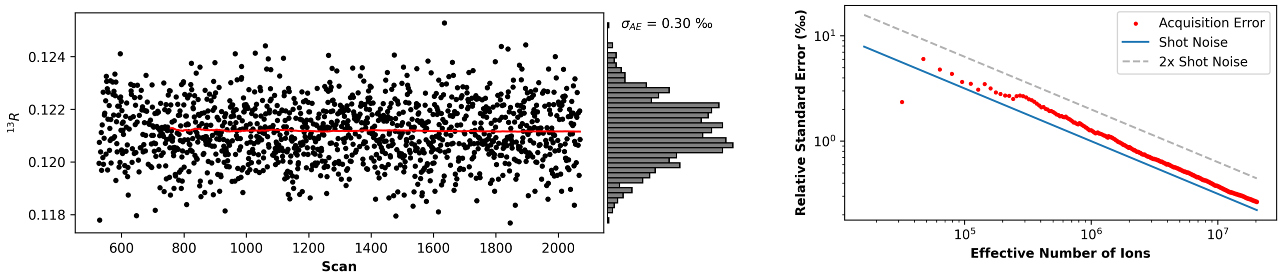

Replicate: 2; Solution: A; Concentration: 1.0 uM

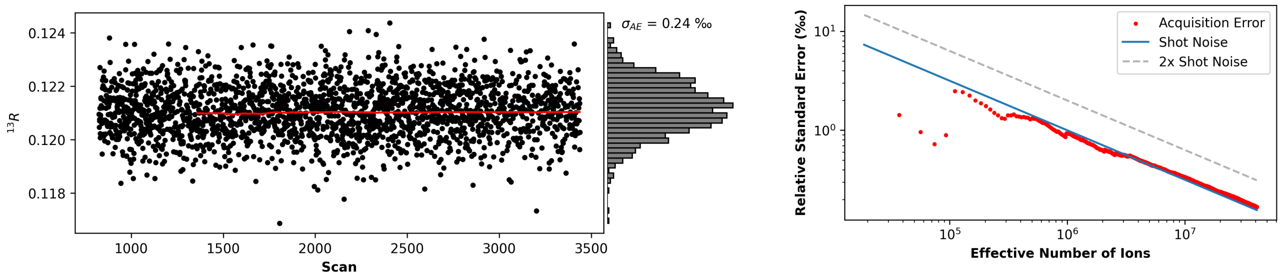

Replicate: 2; Solution: B; Concentration: 1.0 uM

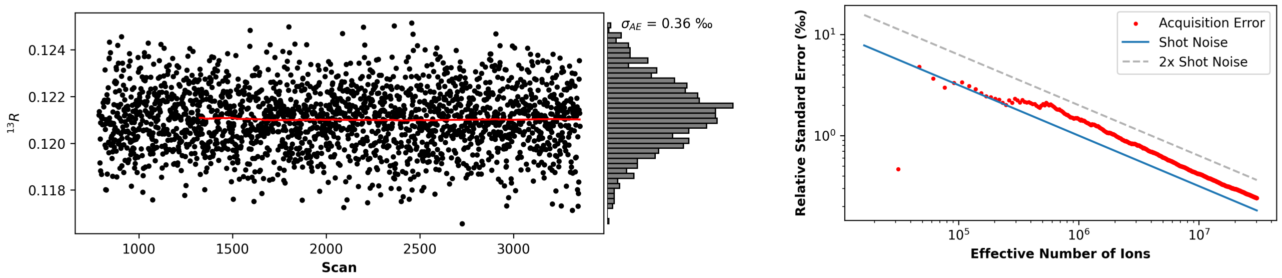

Replicate: 2; Solution: D; Concentration: 1.0 uM

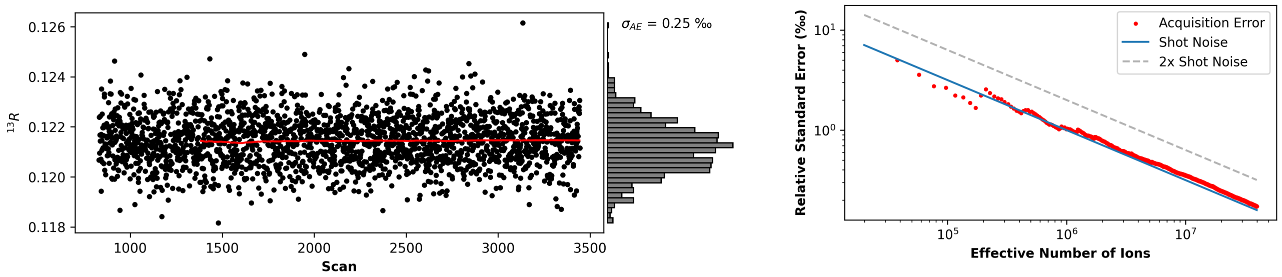

Replicate: 2; Solution: C; Concentration: 5.0 uM

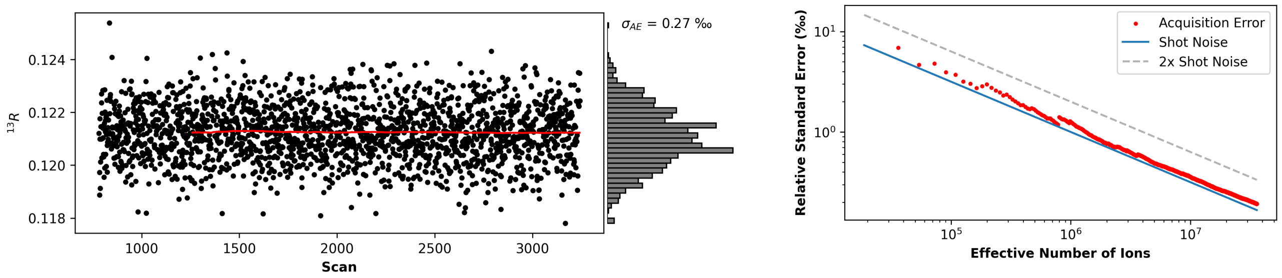

Replicate: 2; Solution: D; Concentration: 5.0 uM

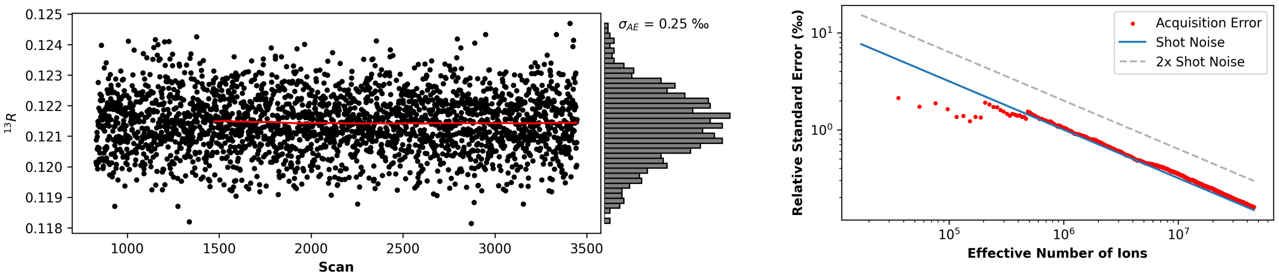

Replicate: 2; Solution: B; Concentration: 5.0 uM

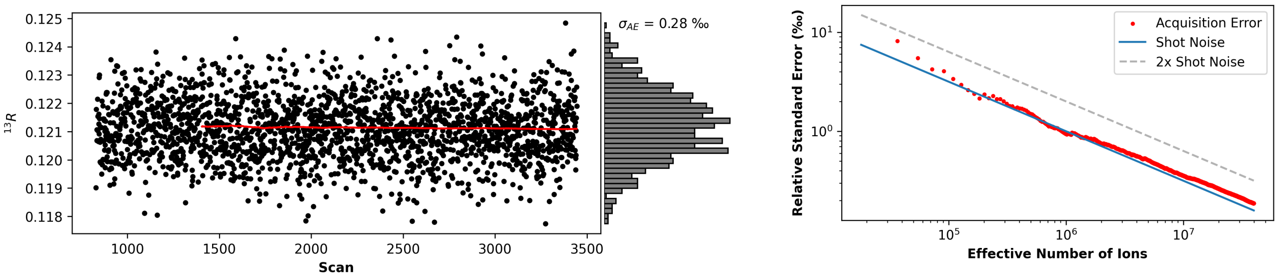

Replicate: 2; Solution: A; Concentration: 5.0 uM

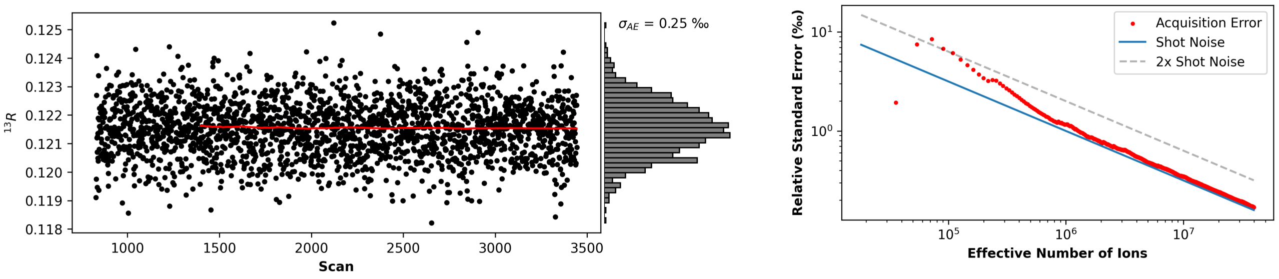

Replicate: 2; Solution: D; Concentration: 25.0 uM

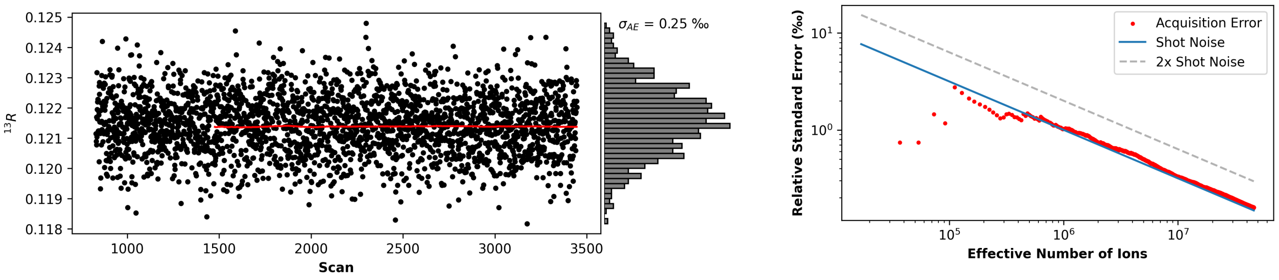

Replicate: 2; Solution: A; Concentration: 25.0 uM

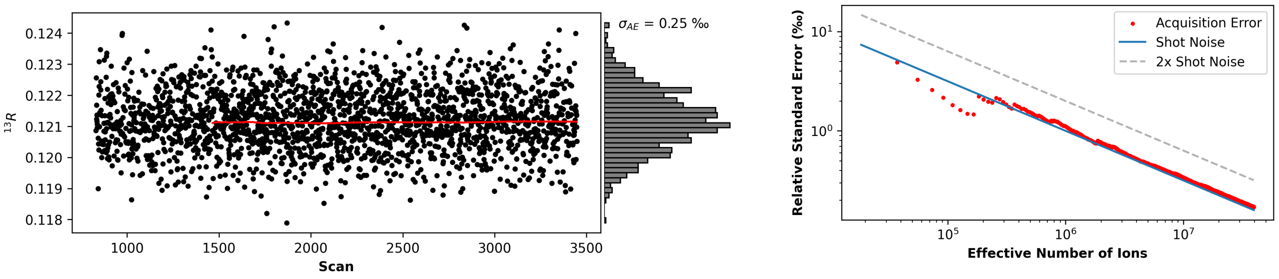

Replicate: 2; Solution: C; Concentration: 25.0 uM

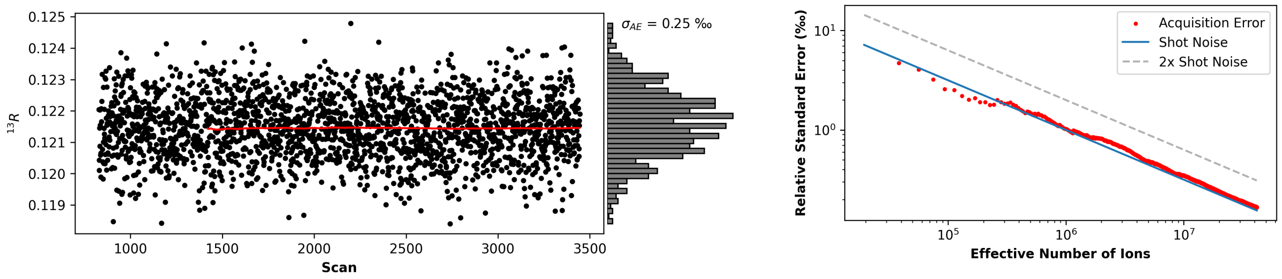

Replicate: 2; Solution: B; Concentration: 25.0 uM

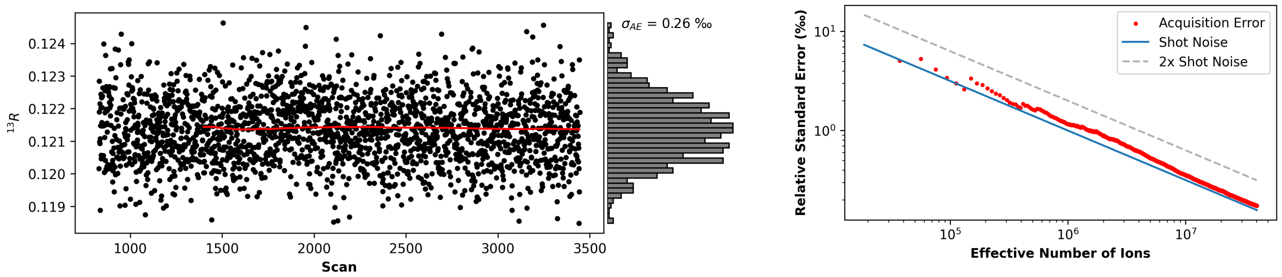

Replicate: 2; Solution: C; Concentration: 50.0 uM

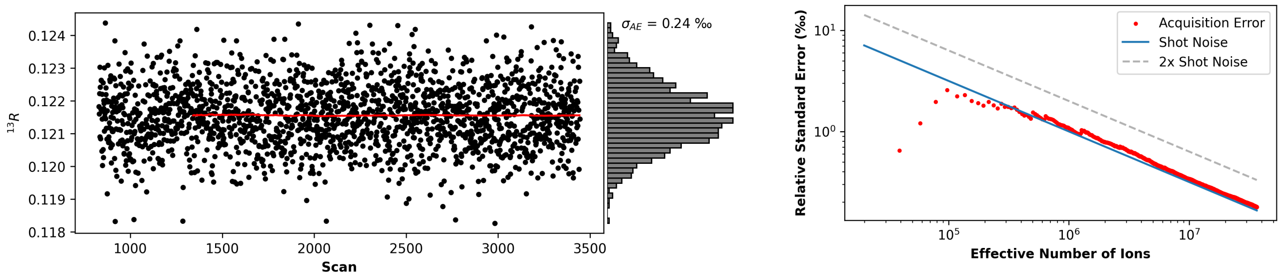

Replicate: 2; Solution: A; Concentration: 50.0 uM

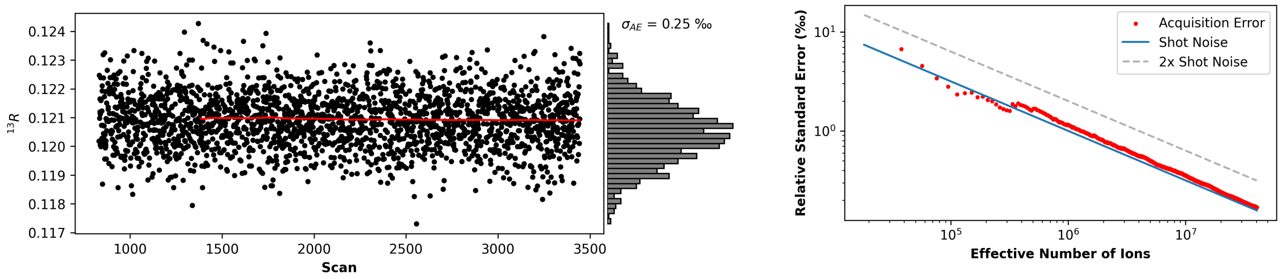

Replicate: 2; Solution: D; Concentration: 50.0 uM

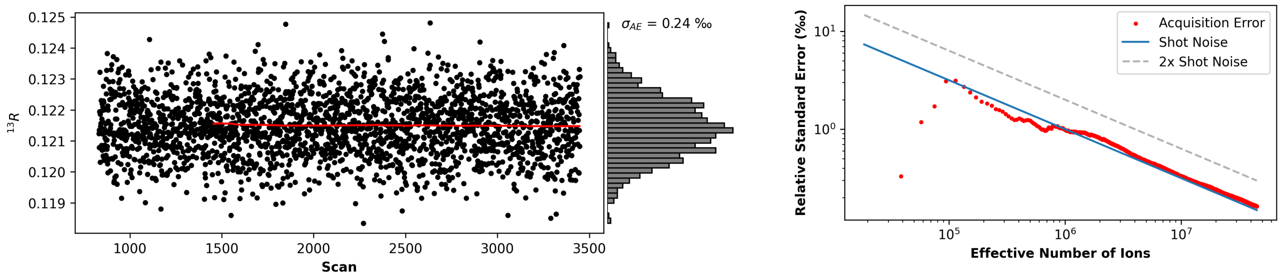

Replicate: 2; Solution: B; Concentration: 50.0 uM

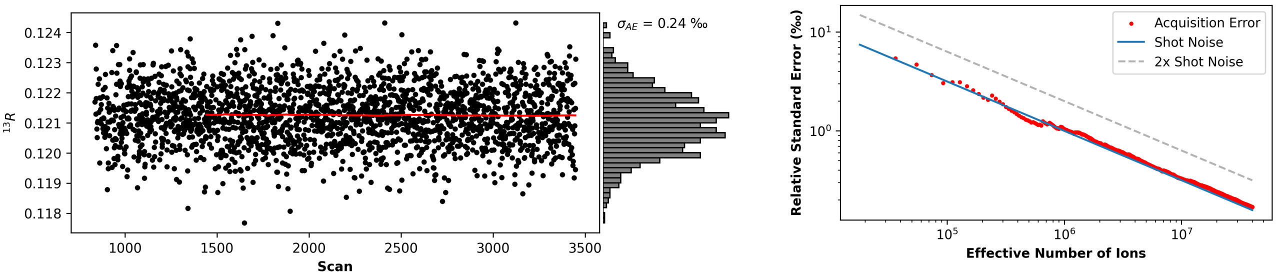

Replicate: 3; Solution: C; Concentration: 0.1 uM

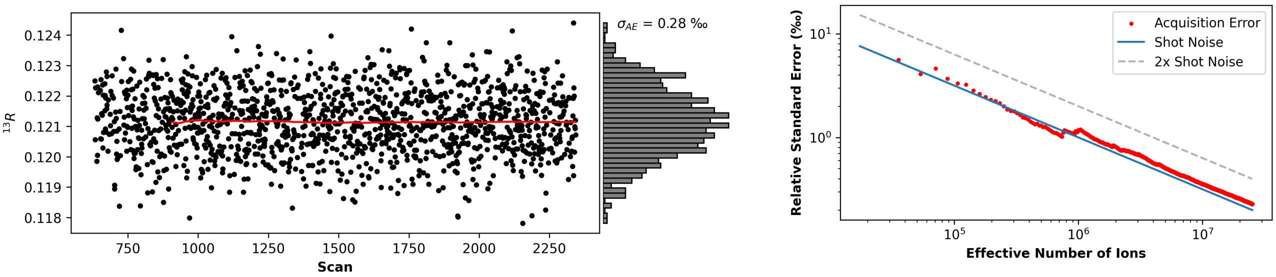

Replicate: 3; Solution: B; Concentration: 0.1 uM

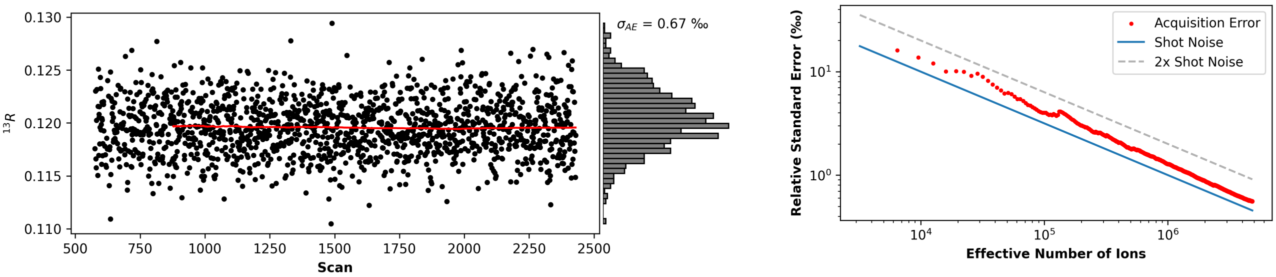

Replicate: 3; Solution: A; Concentration: 0.1 uM

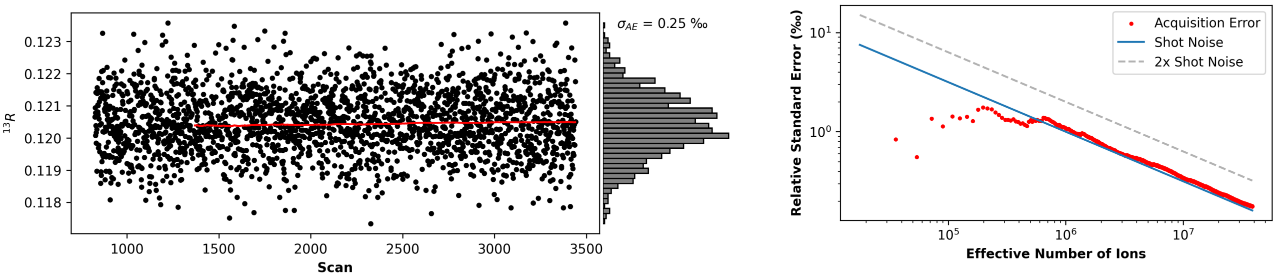

Replicate: 3; Solution: D; Concentration: 0.1 uM

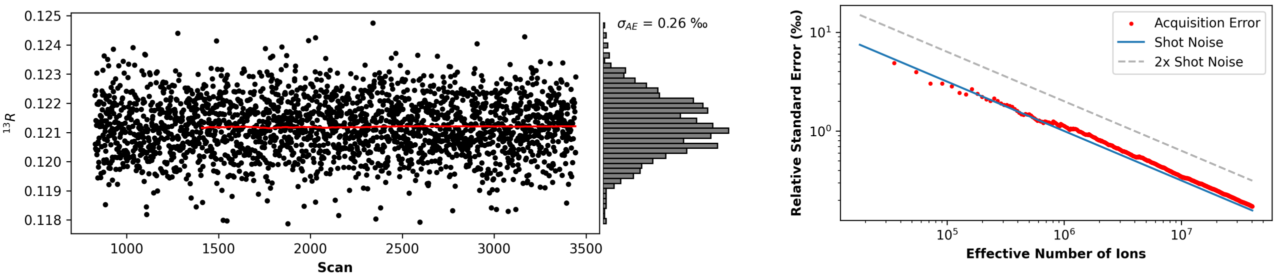

Replicate: 3; Solution: D; Concentration: 1.0 uM

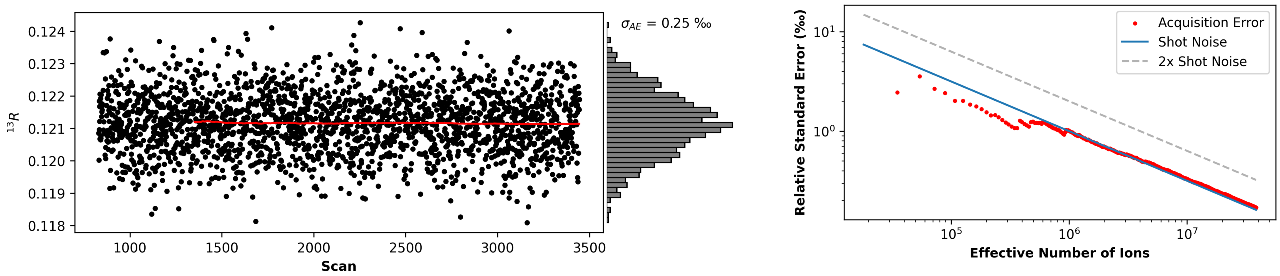

Replicate: 3; Solution: B; Concentration: 1.0 uM

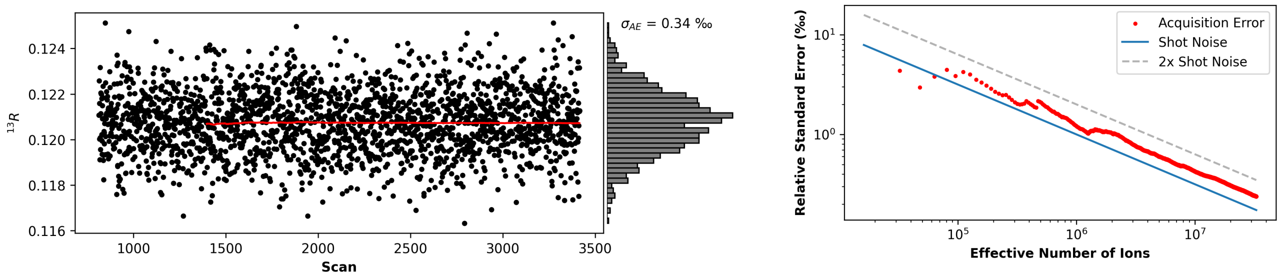

Replicate: 3; Solution: A; Concentration: 1.0 uM

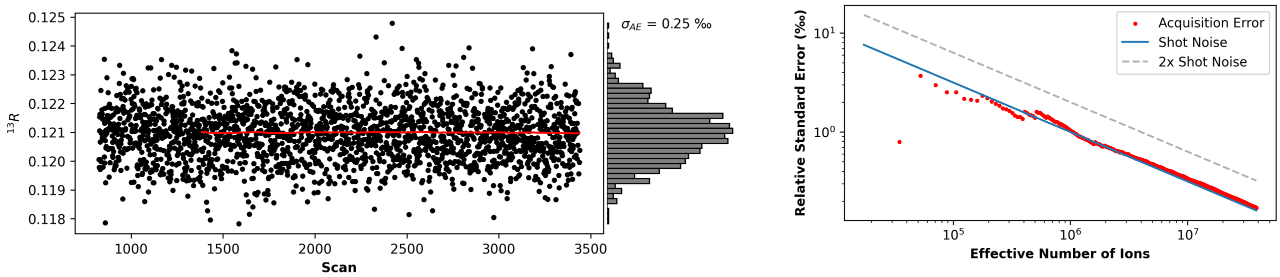

Replicate: 3; Solution: C; Concentration: 1.0 uM

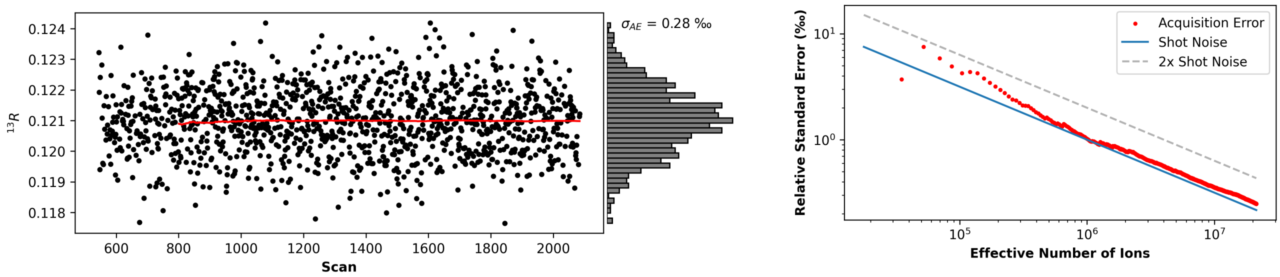

Replicate: 3; Solution: D; Concentration: 5.0 uM

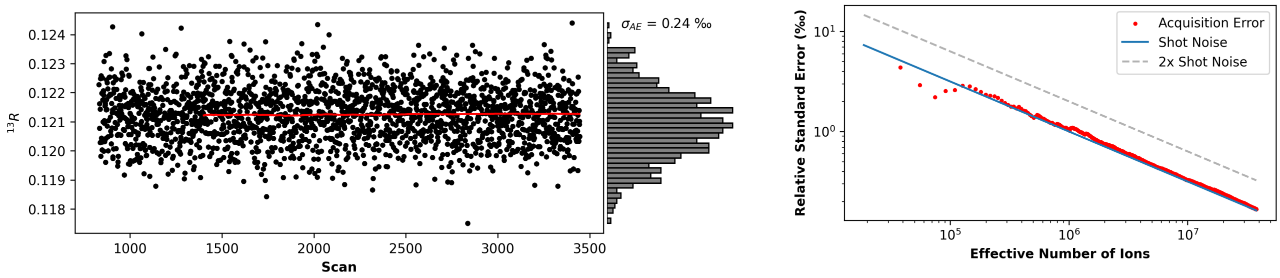

Replicate: 3; Solution: B; Concentration: 5.0 uM

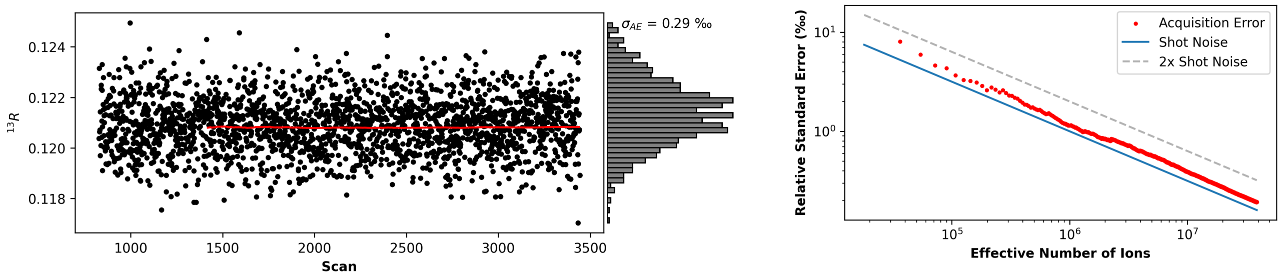

Replicate: 3; Solution: A; Concentration: 5.0 uM

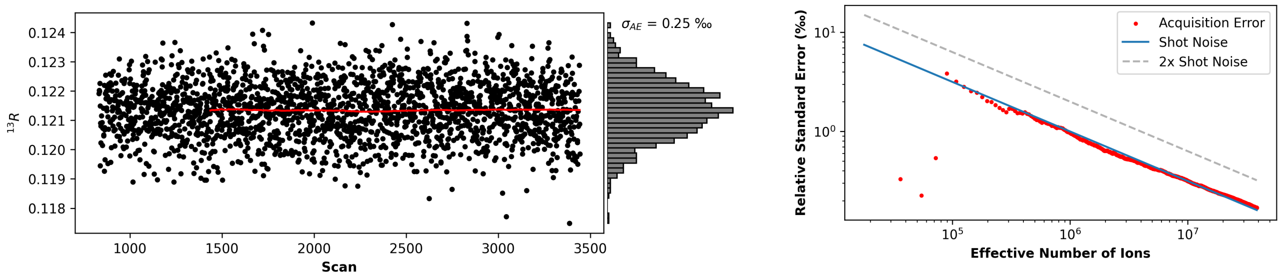

Replicate: 3; Solution: C; Concentration: 5.0 uM

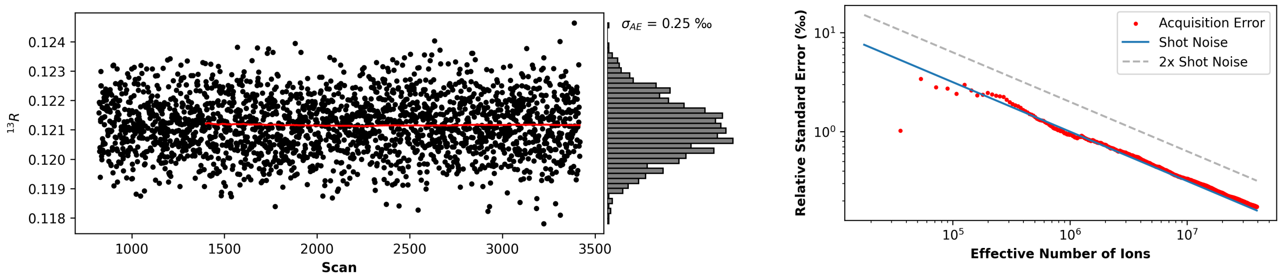

Replicate: 3; Solution: B; Concentration: 25.0 uM

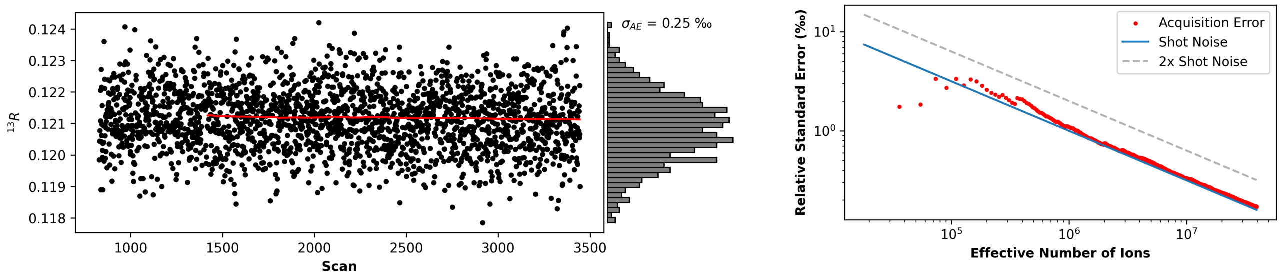

Replicate: 3; Solution: A; Concentration: 25.0 uM

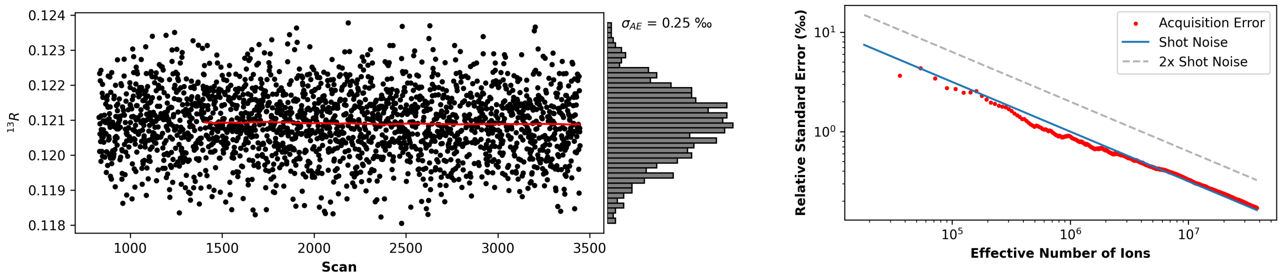

Replicate: 3; Solution: C; Concentration: 25.0 uM

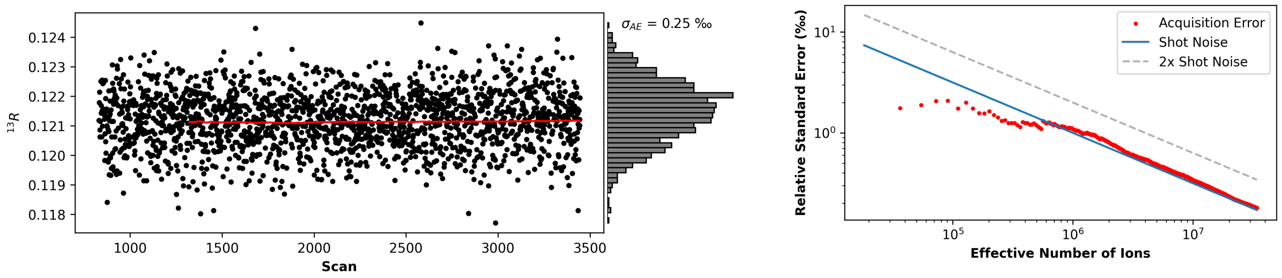

Replicate: 3; Solution: D; Concentration: 25.0 uM

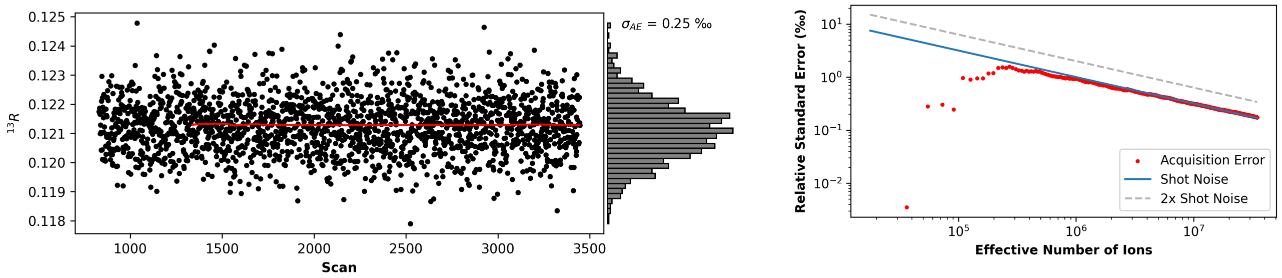

Replicate: 3; Solution: A; Concentration: 50.0 uM

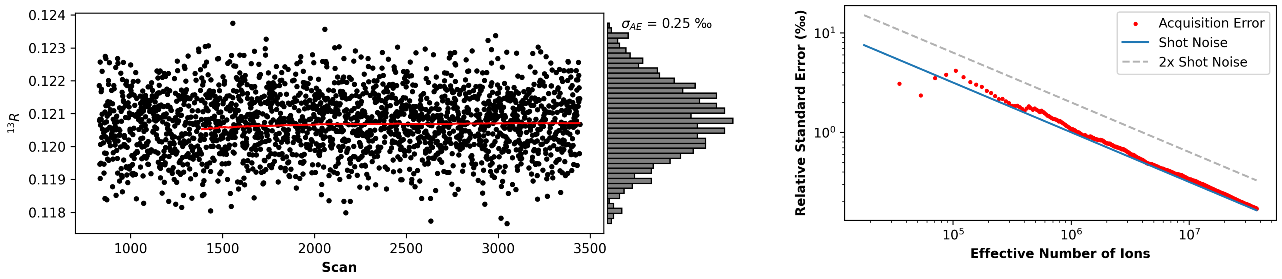

Replicate: 3; Solution: B; Concentration: 50.0 uM

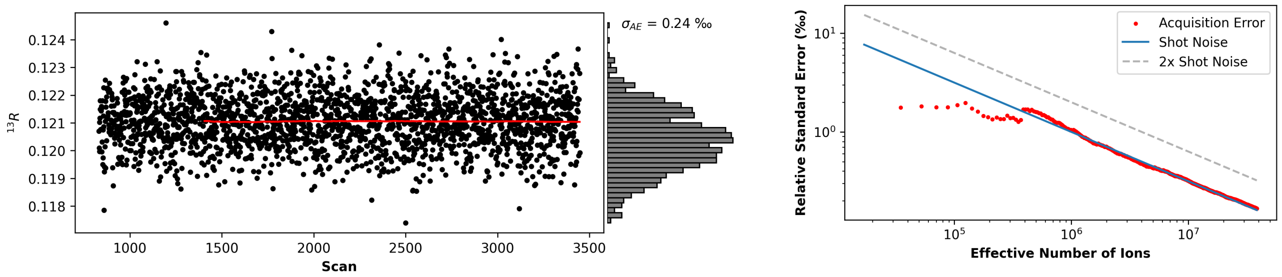

Replicate: 3; Solution: C; Concentration: 50.0 uM

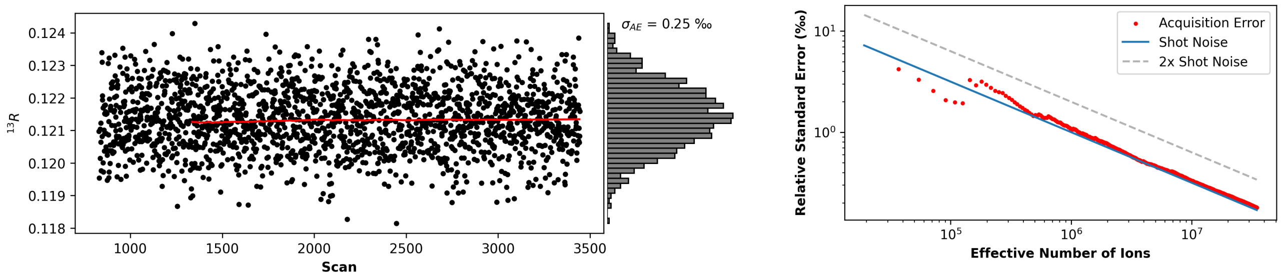

Replicate: 3; Solution: D; Concentration: 50.0 uM

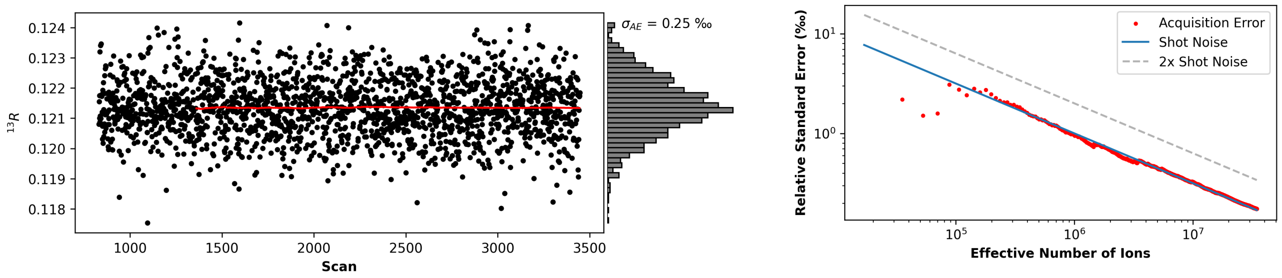

Replicate: 4; Solution: A; Concentration: 0.1 uM

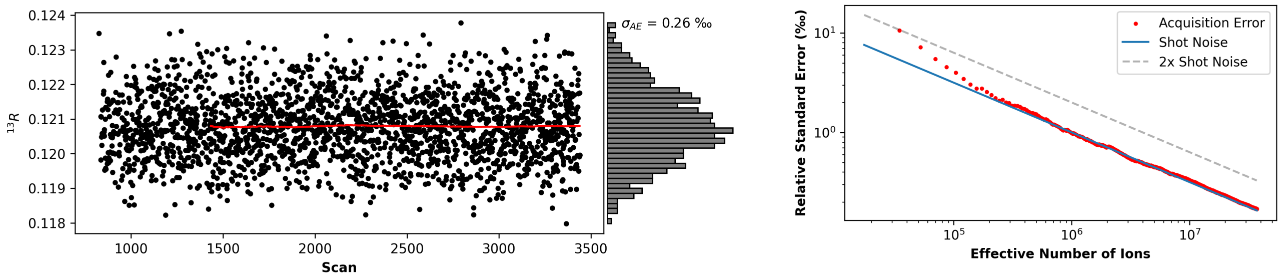

Replicate: 4; Solution: A; Concentration: 1.0 uM

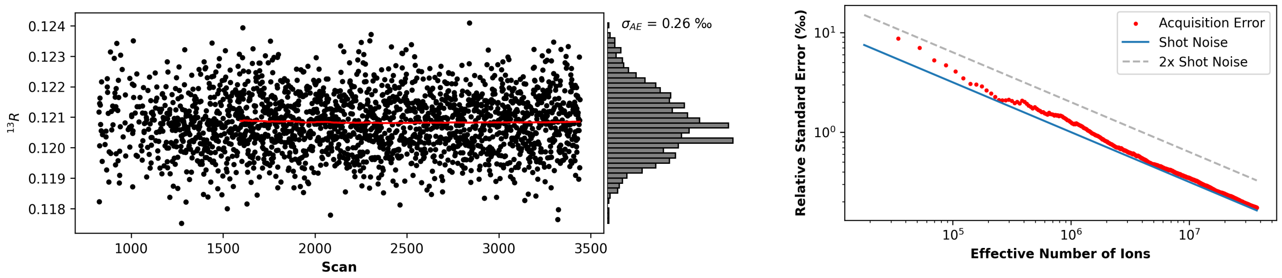

Replicate: 4; Solution: A; Concentration: 5.0 uM

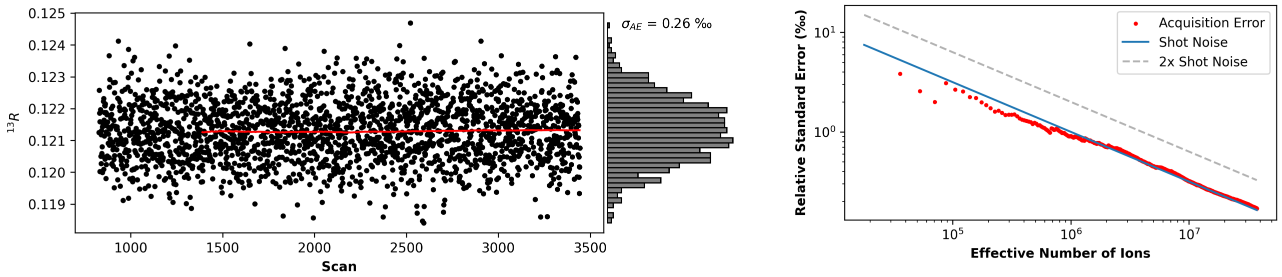

Replicate: 4; Solution: A; Concentration: 25.0 uM

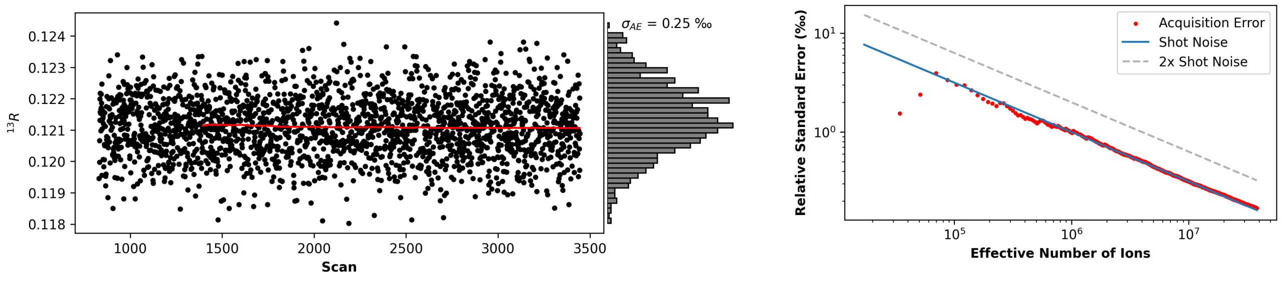

Replicate: 4; Solution: A; Concentration: 50.0 uM

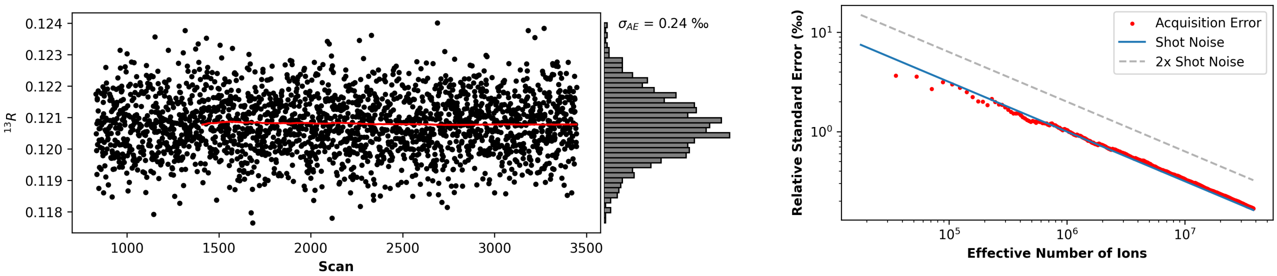

σ_{AE} = 0.24 ‰
^{13}R
Scan
Relative Standard Error (‰)
Effective Number of Ions
Acquisition Error
Shot Noise
2x Shot Noise

4. Virtual Bracketing and Delta Calculation Summary

Deltas were calculated by 'Standardization Of Block Average Ratios'

THN Mix

THN Mix - Standard Concentration: 0.1 uM; Sample Concentration: 0.1

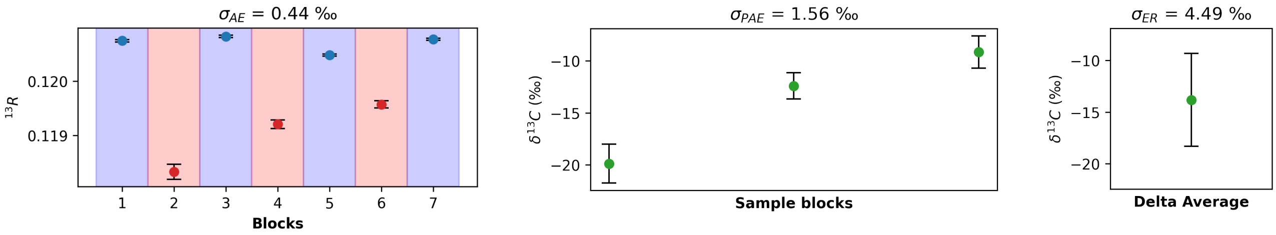

THN Mix - Standard Concentration: 1 uM; Sample Concentration: 0.1

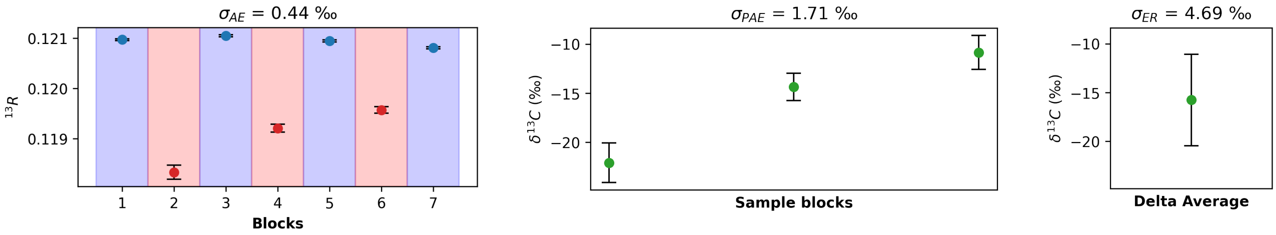

THN Mix - Standard Concentration: 5 uM; Sample Concentration: 0.1

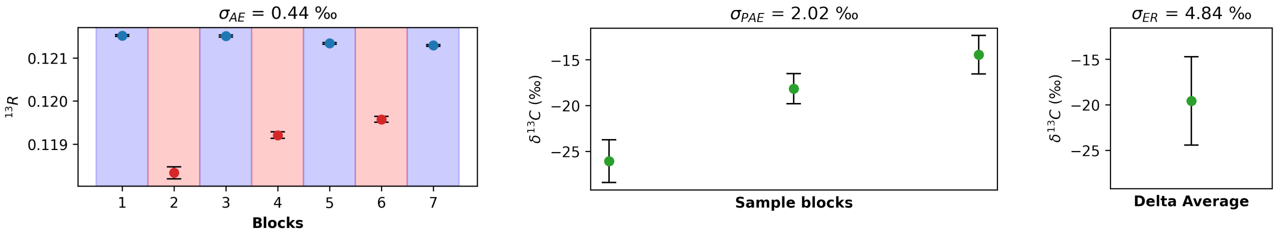

THN Mix - Standard Concentration: 25 uM; Sample Concentration: 0.1

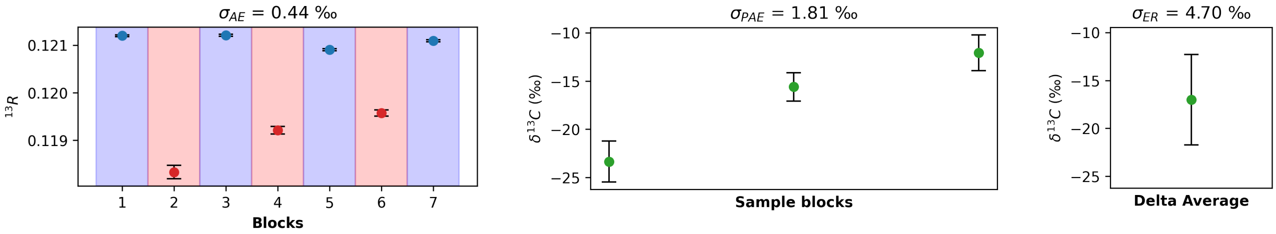

THN Mix - Standard Concentration: 50 uM; Sample Concentration: 0.1

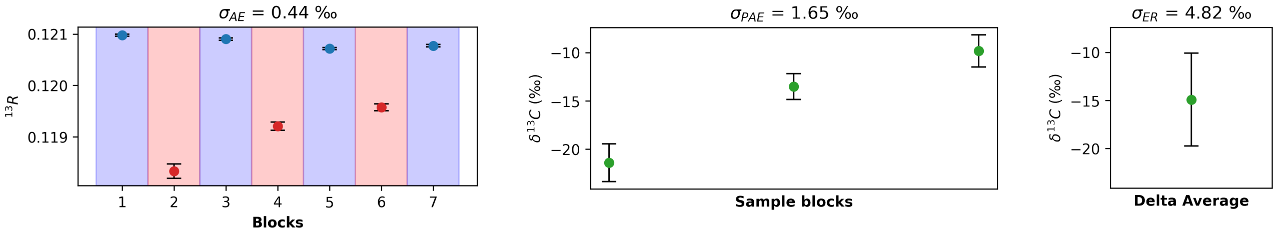

THN Mix - Standard Concentration: 0.1 uM; Sample Concentration: 1

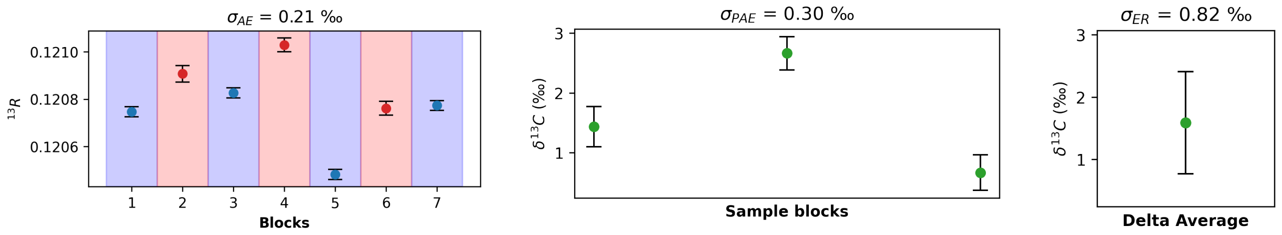

THN Mix - Standard Concentration: 1 uM; Sample Concentration: 1

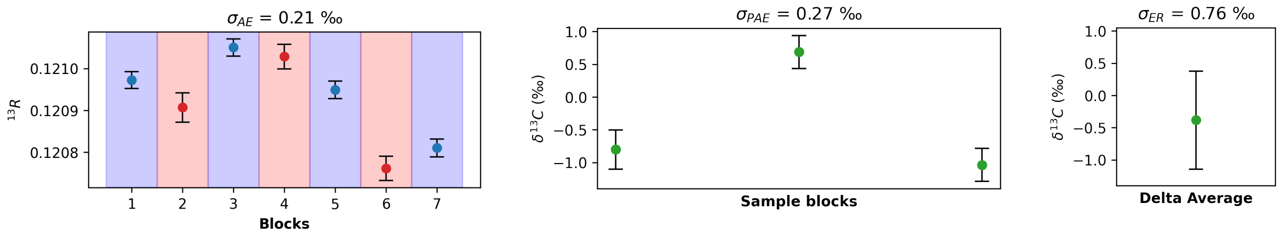

THN Mix - Standard Concentration: 5 uM; Sample Concentration: 1

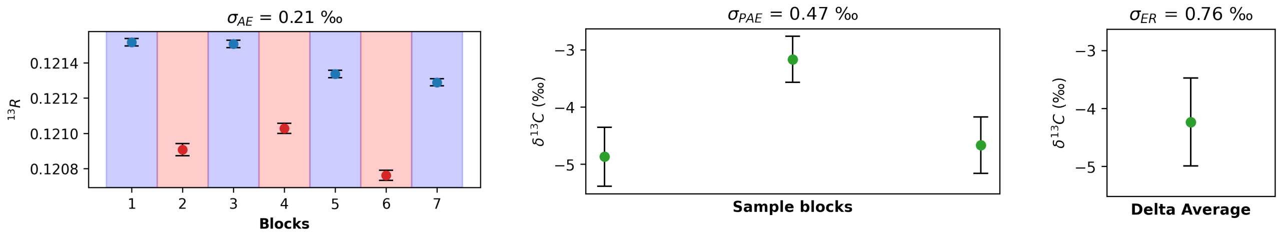

THN Mix - Standard Concentration: 25 uM; Sample Concentration: 1

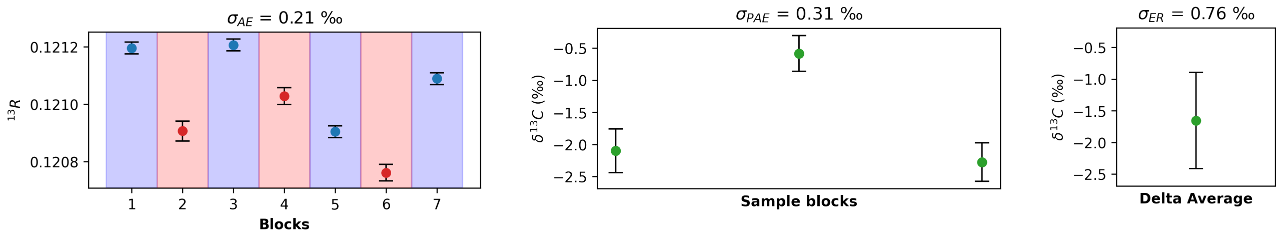

THN Mix - Standard Concentration: 50 uM; Sample Concentration: 1

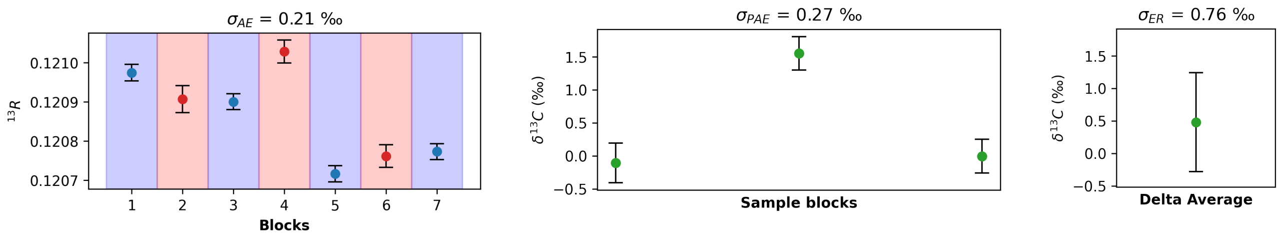

THN Mix - Standard Concentration: 0.1 uM; Sample Concentration: 5

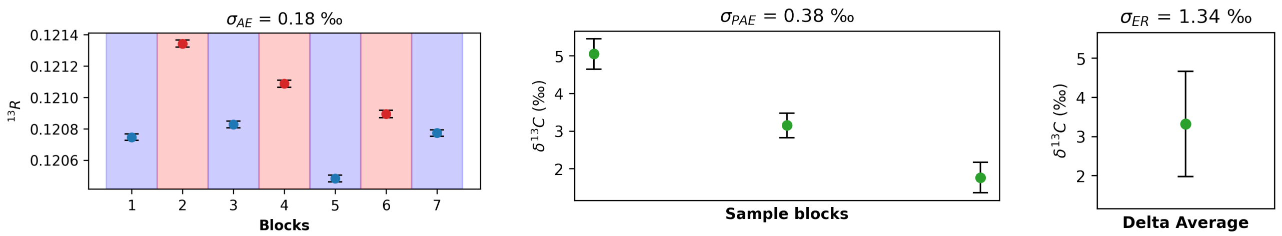

THN Mix - Standard Concentration: 1 uM; Sample Concentration: 5

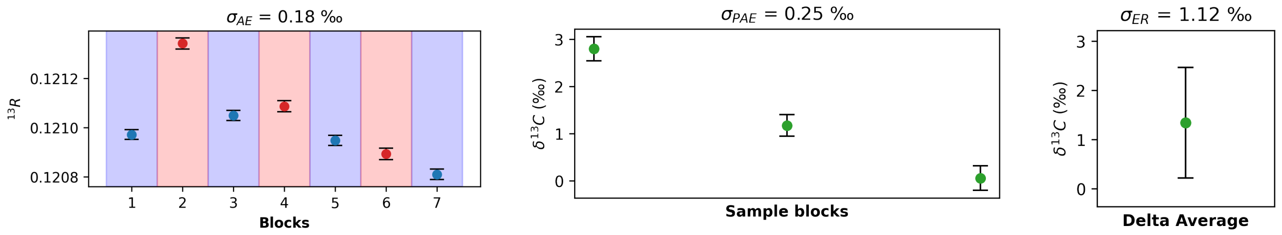

THN Mix - Standard Concentration: 5 uM; Sample Concentration: 5

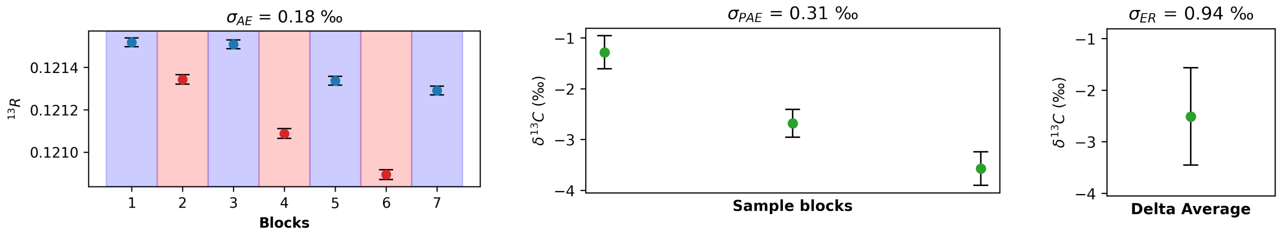

THN Mix - Standard Concentration: 25 uM; Sample Concentration: 5

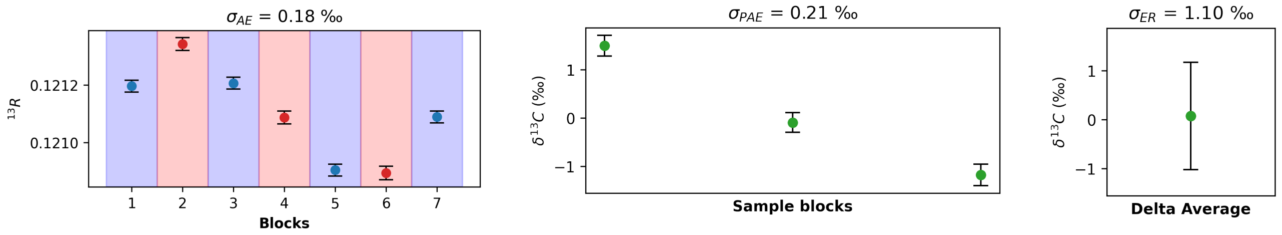

THN Mix - Standard Concentration: 50 uM; Sample Concentration: 5

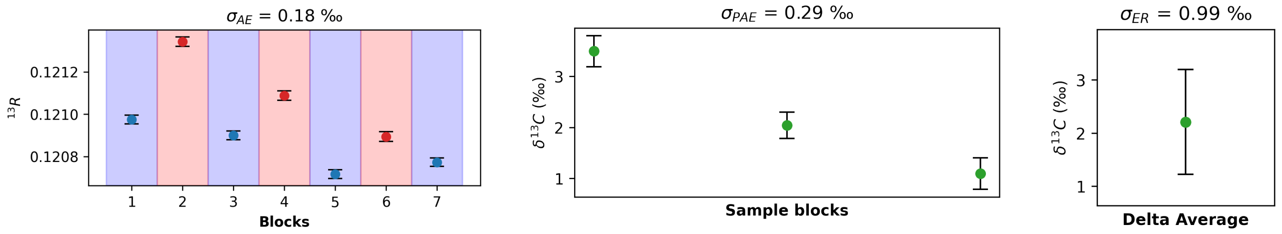

THN Mix - Standard Concentration: 0.1 uM; Sample Concentration: 25

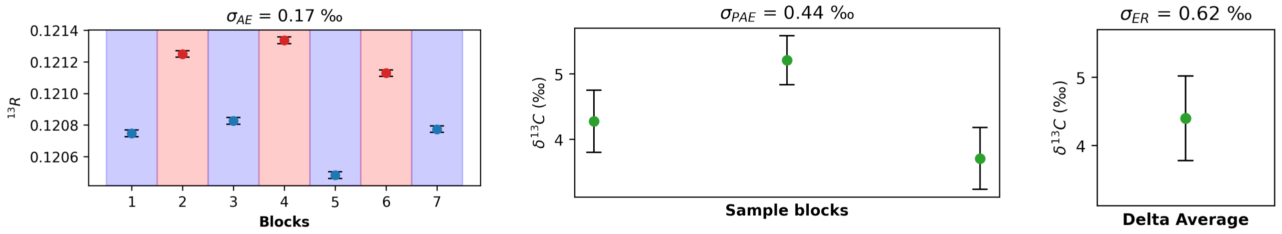

THN Mix - Standard Concentration: 1 uM; Sample Concentration: 25

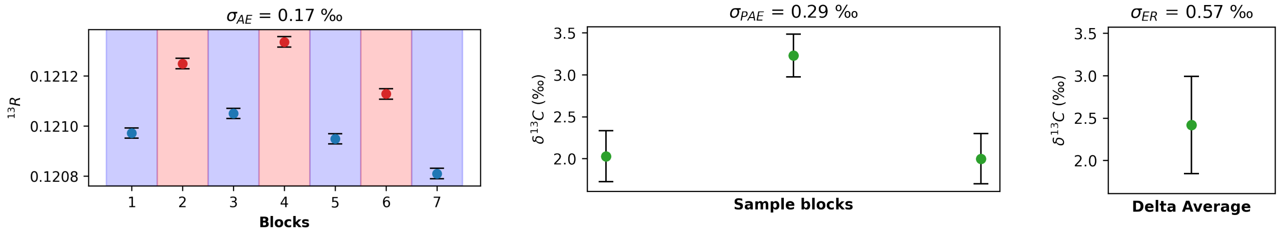

THN Mix - Standard Concentration: 5 uM; Sample Concentration: 25

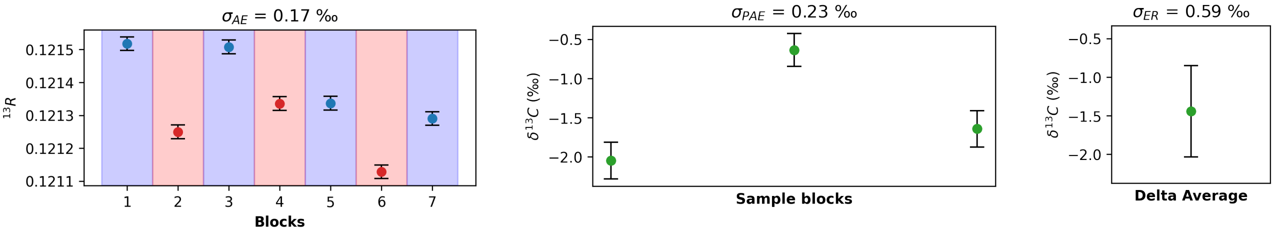

THN Mix - Standard Concentration: 25 uM; Sample Concentration: 25

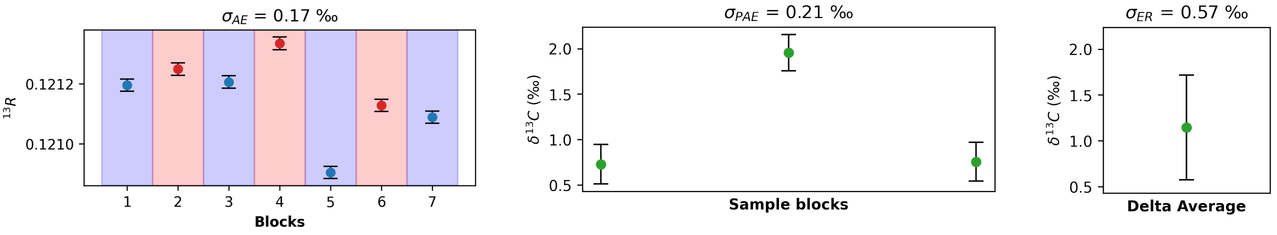

THN Mix - Standard Concentration: 50 uM; Sample Concentration: 25

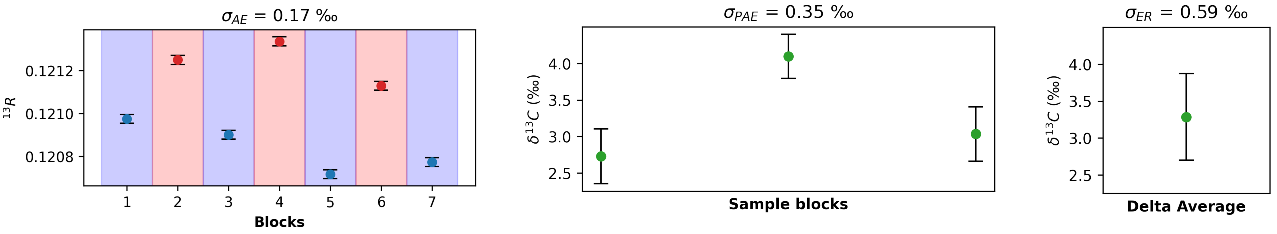

THN Mix - Standard Concentration: 0.1 uM; Sample Concentration: 50

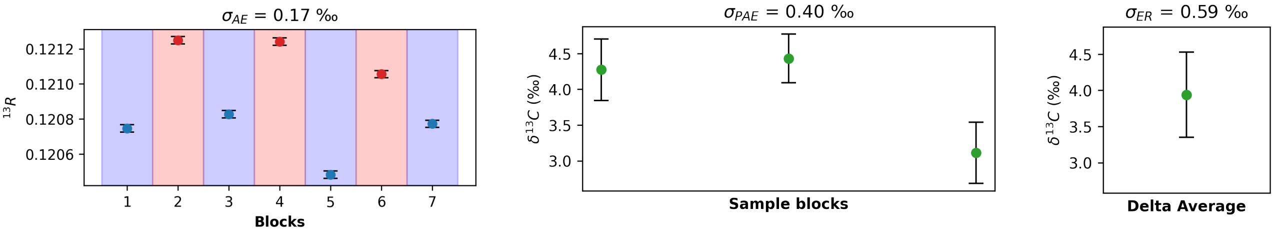

THN Mix - Standard Concentration: 1 uM; Sample Concentration: 50

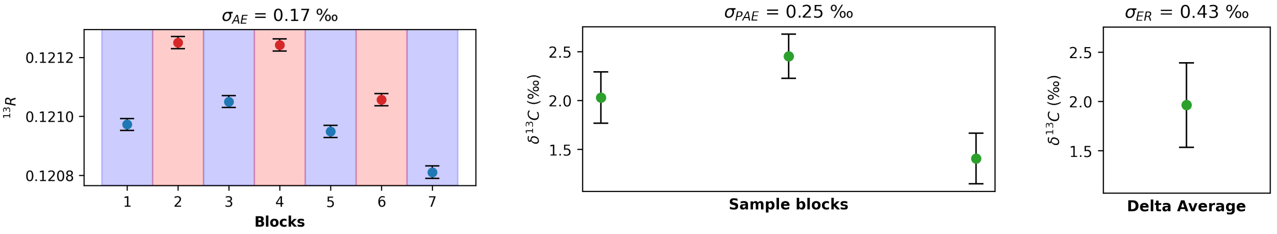

THN Mix - Standard Concentration: 5 uM; Sample Concentration: 50

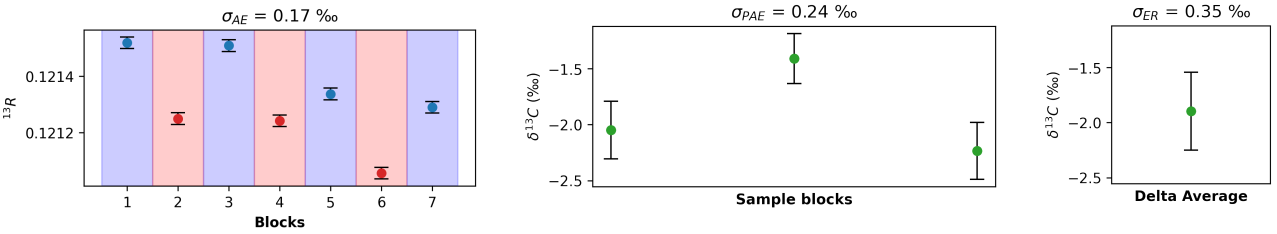

THN Mix - Standard Concentration: 25 uM; Sample Concentration: 50

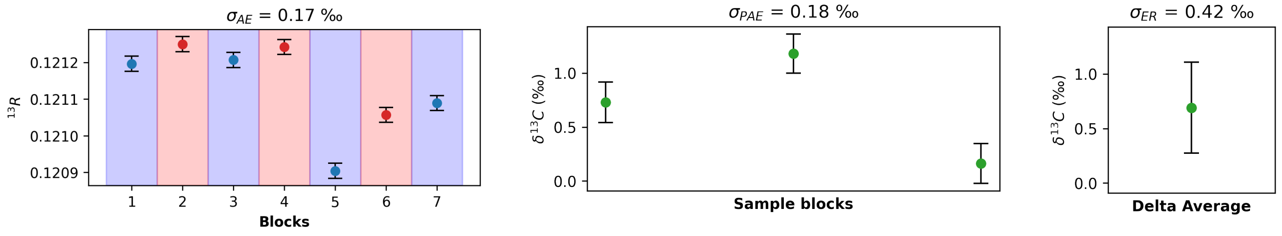

THN Mix - Standard Concentration: 50 uM; Sample Concentration: 50

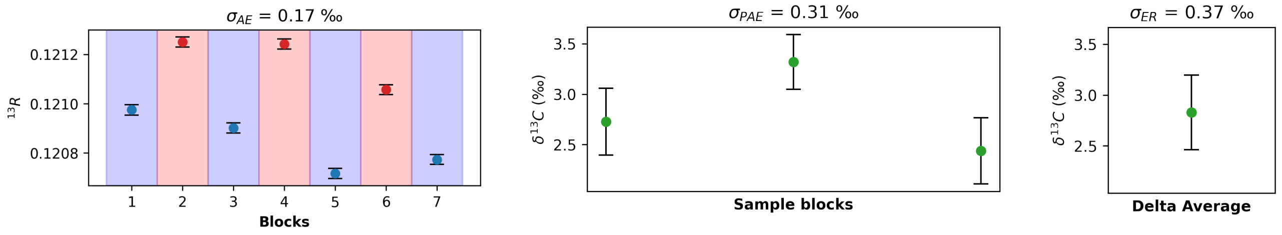

THN Mix + NH4OH 1%

THN Mix + NH4OH 1% - Standard Concentration: 0.1 uM; Sample Concentration: 0.1

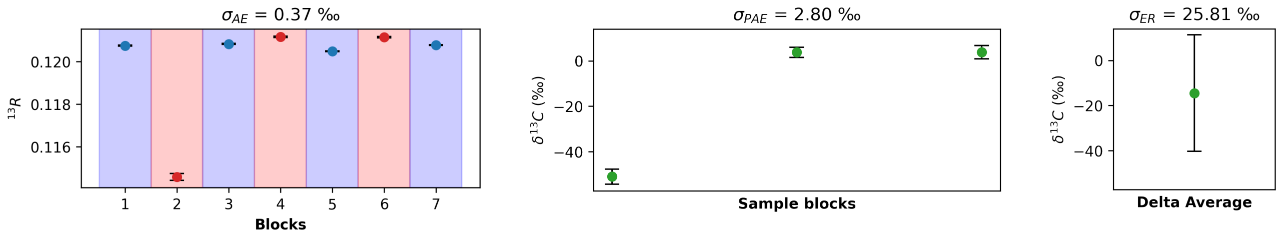

THN Mix + NH4OH 1% - Standard Concentration: 1 uM; Sample Concentration: 0.1

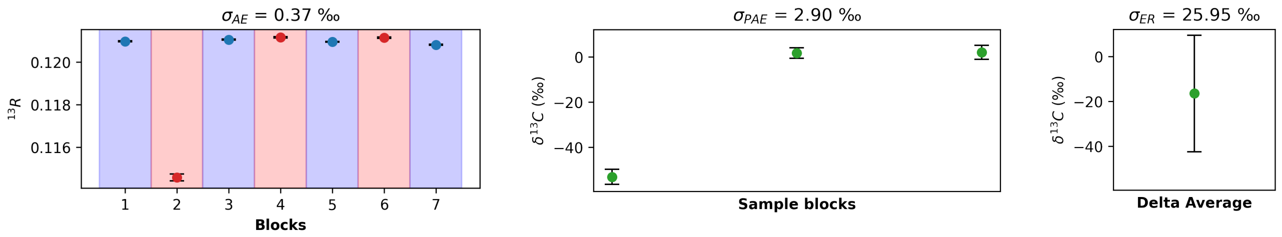

THN Mix + NH4OH 1% - Standard Concentration: 5 uM; Sample Concentration: 0.1

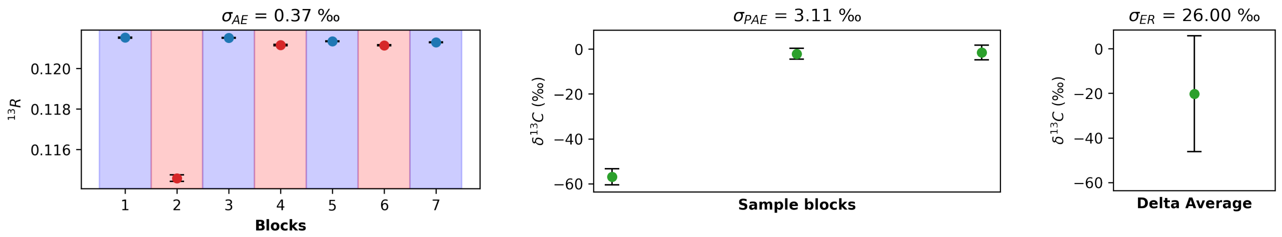

THN Mix + NH4OH 1% - Standard Concentration: 25 uM; Sample Concentration: 0.1

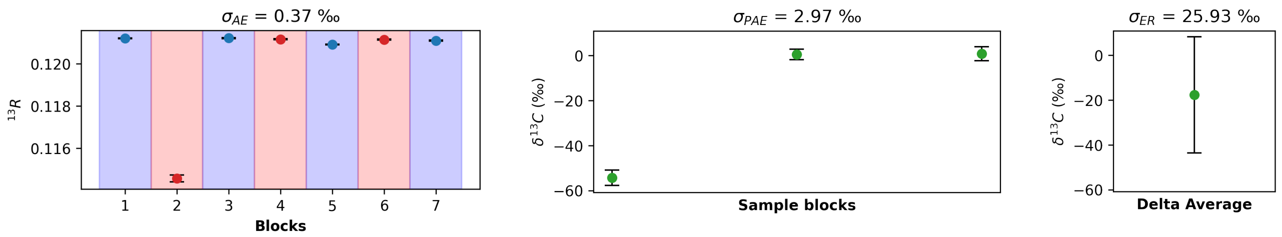

THN Mix + NH4OH 1% - Standard Concentration: 50 uM; Sample Concentration: 0.1

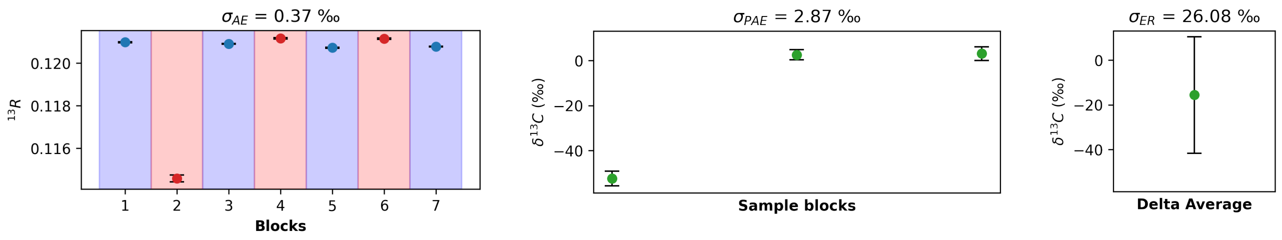

THN Mix + NH4OH 1% - Standard Concentration: 0.1 uM; Sample Concentration: 1

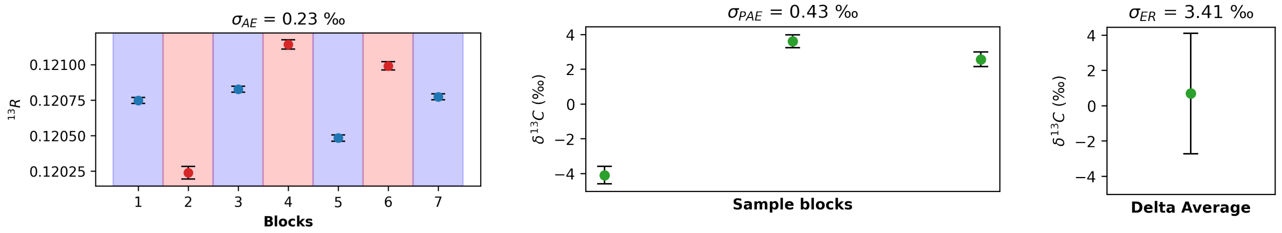

THN Mix + NH4OH 1% - Standard Concentration: 1 uM; Sample Concentration: 1

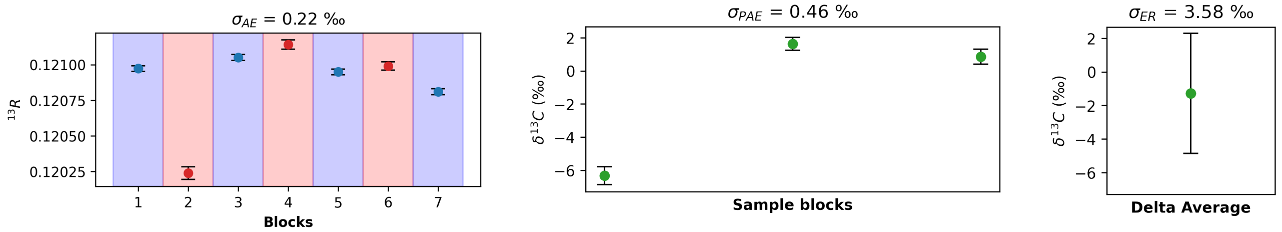

THN Mix + NH4OH 1% - Standard Concentration: 5 uM; Sample Concentration: 1

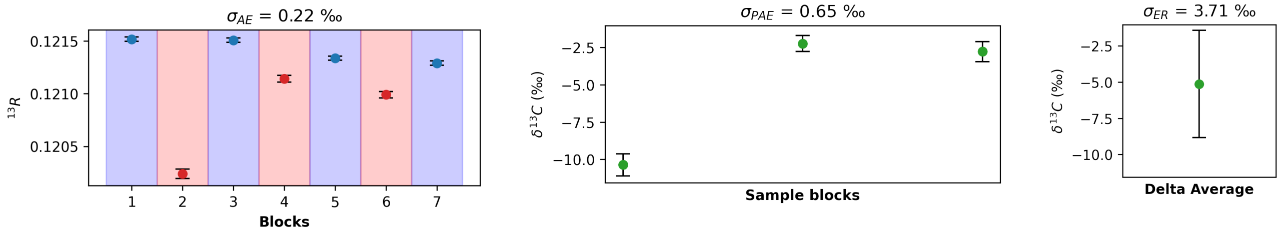

THN Mix + NH4OH 1% - Standard Concentration: 25 uM; Sample Concentration: 1

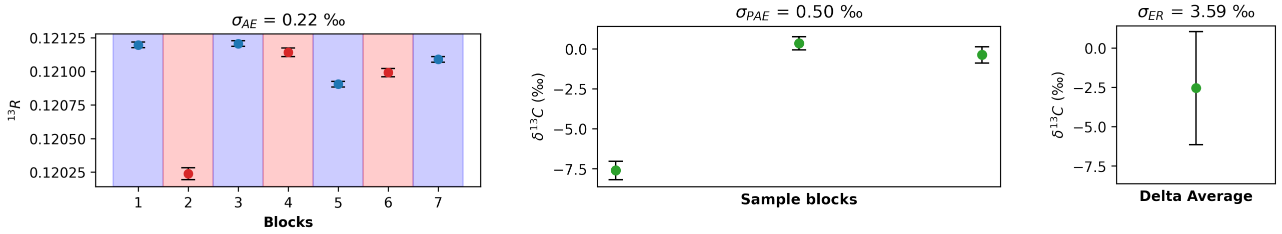

THN Mix + NH4OH 1% - Standard Concentration: 50 uM; Sample Concentration: 1

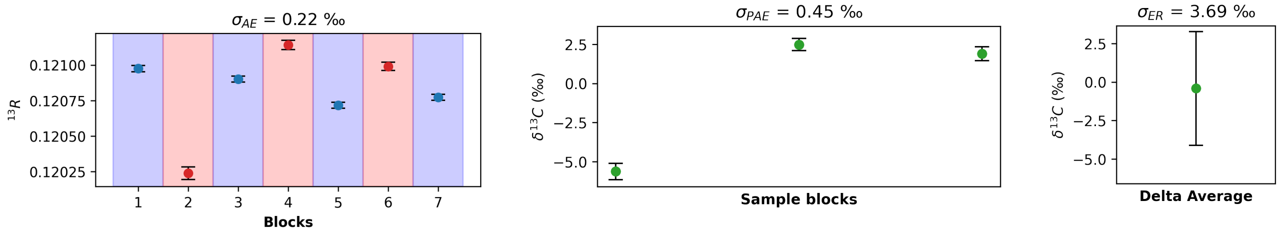

THN Mix + NH4OH 1% - Standard Concentration: 0.1 uM; Sample Concentration: 5

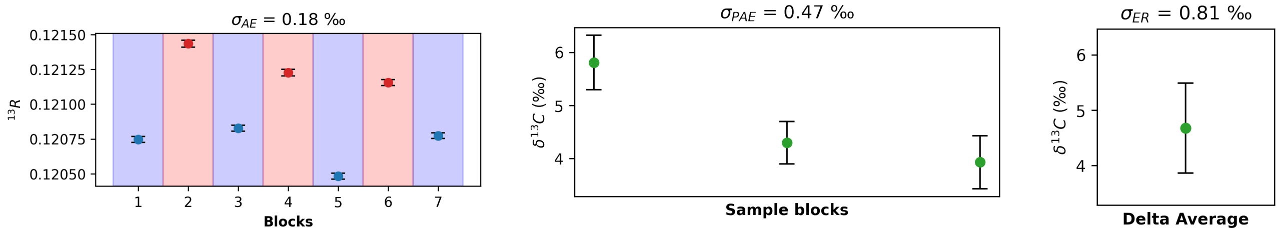

THN Mix + NH4OH 1% - Standard Concentration: 1 uM; Sample Concentration: 5

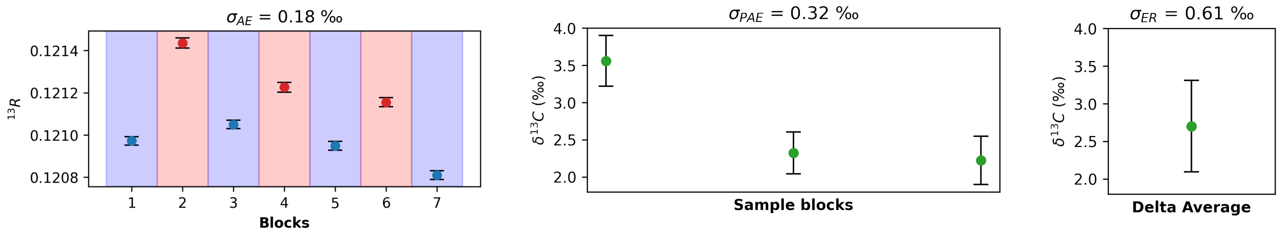

THN Mix + NH4OH 1% - Standard Concentration: 5 uM; Sample Concentration: 5

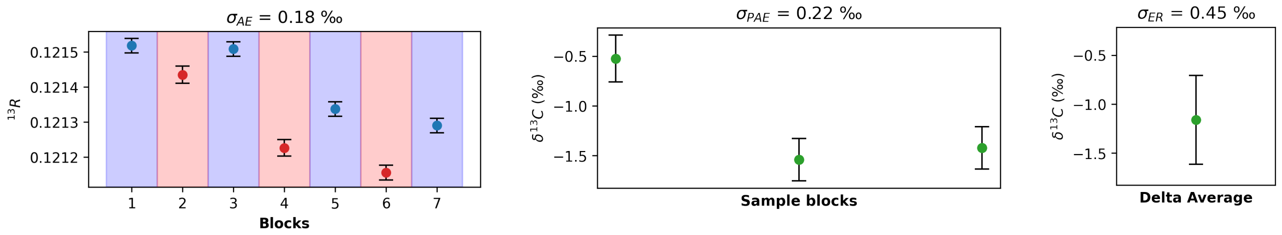

THN Mix + NH4OH 1% - Standard Concentration: 25 uM; Sample Concentration: 5

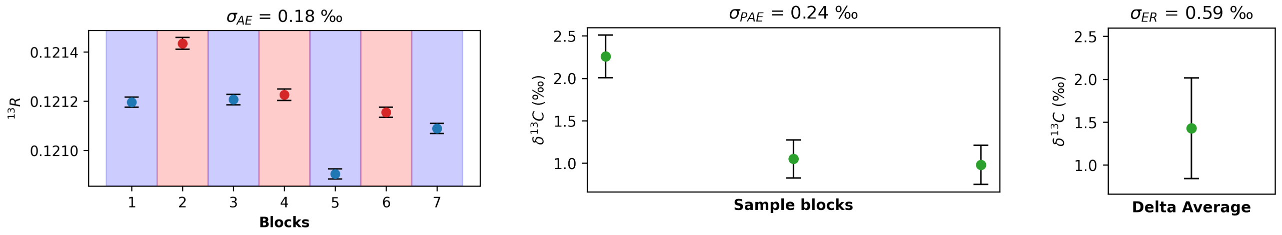

THN Mix + NH4OH 1% - Standard Concentration: 50 uM; Sample Concentration: 5

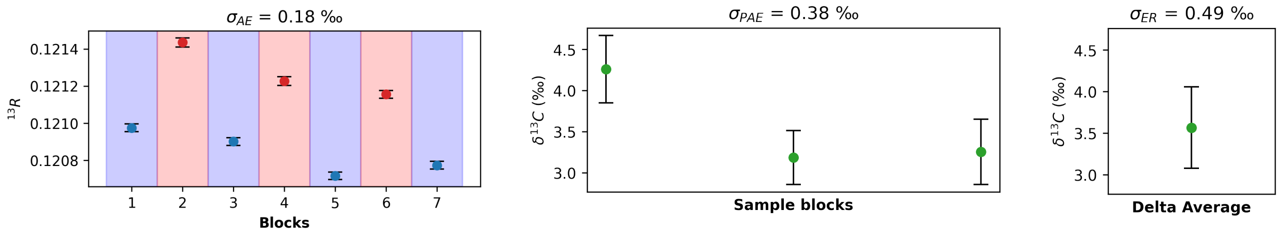

THN Mix + NH4OH 1% - Standard Concentration: 0.1 uM; Sample Concentration: 25

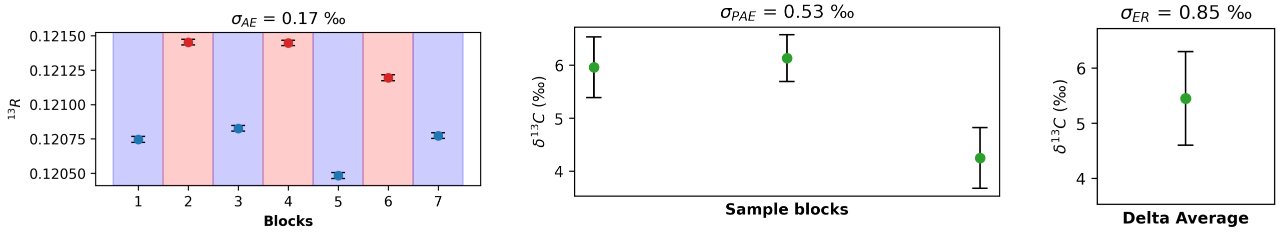

THN Mix + NH4OH 1% - Standard Concentration: 1 uM; Sample Concentration: 25

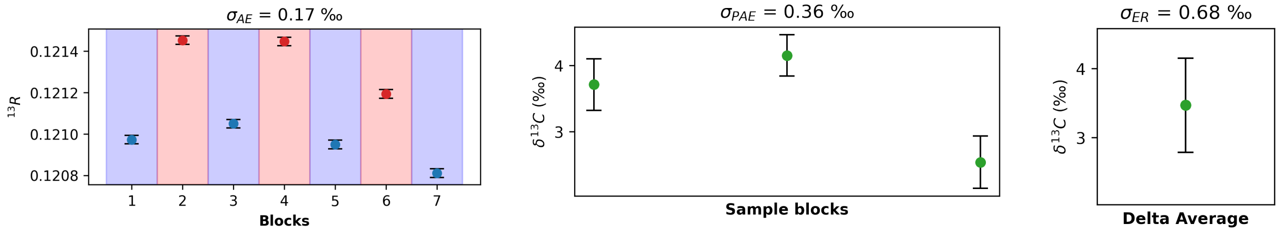

THN Mix + NH4OH 1% - Standard Concentration: 5 uM; Sample Concentration: 25

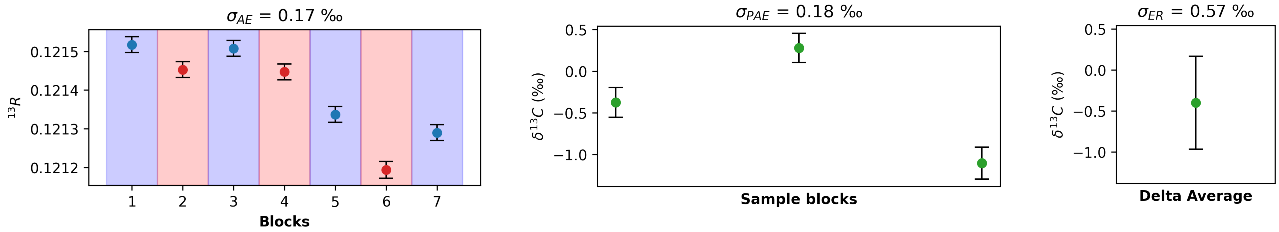

THN Mix + NH4OH 1% - Standard Concentration: 25 uM; Sample Concentration: 25

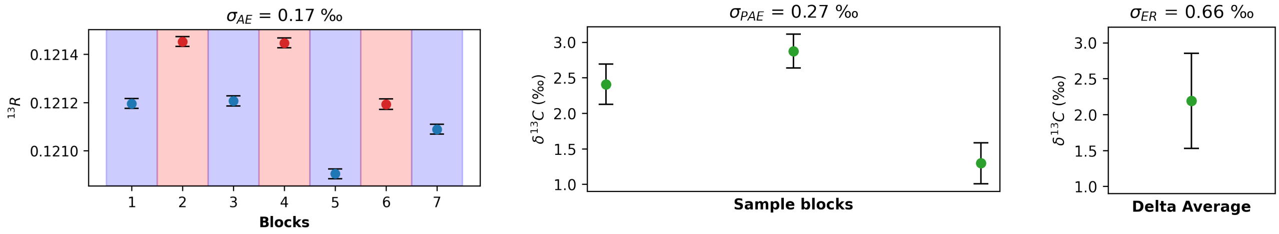

THN Mix + NH4OH 1% - Standard Concentration: 50 uM; Sample Concentration: 25

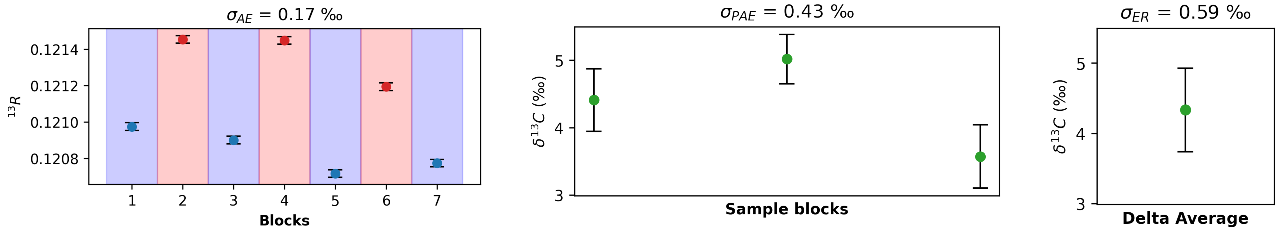

THN Mix + NH4OH 1% - Standard Concentration: 0.1 uM; Sample Concentration: 50

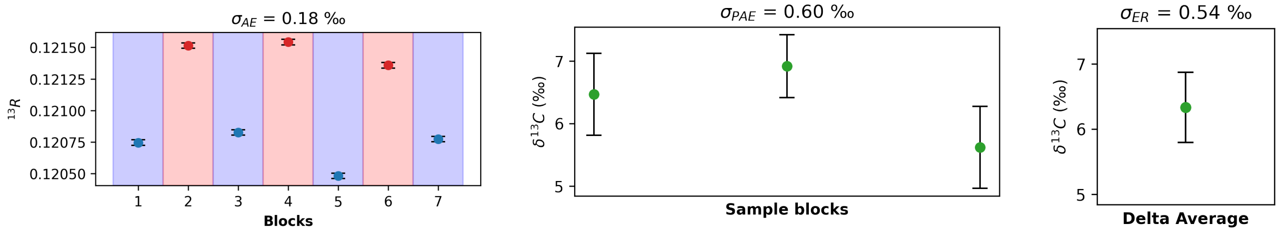

THN Mix + NH4OH 1% - Standard Concentration: 1 uM; Sample Concentration: 50

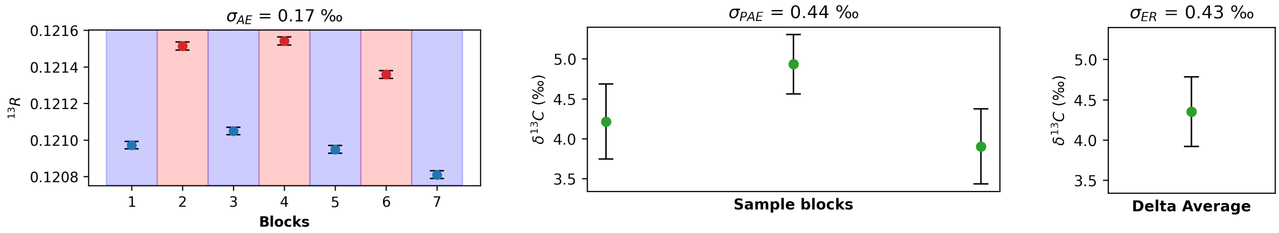

THN Mix + NH4OH 1% - Standard Concentration: 5 uM; Sample Concentration: 50

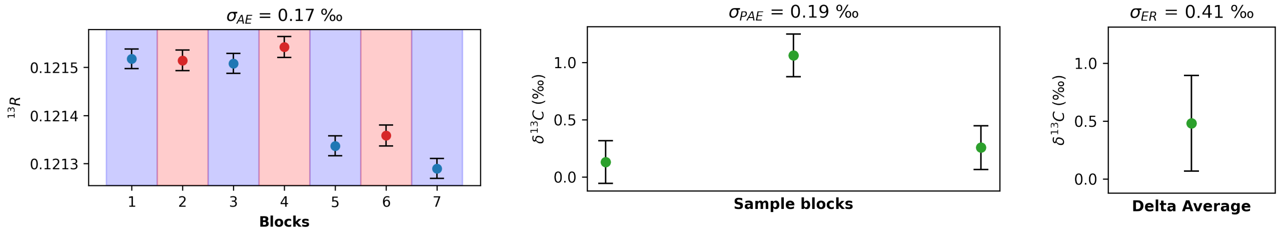

THN Mix + NH4OH 1% - Standard Concentration: 25 uM; Sample Concentration: 50

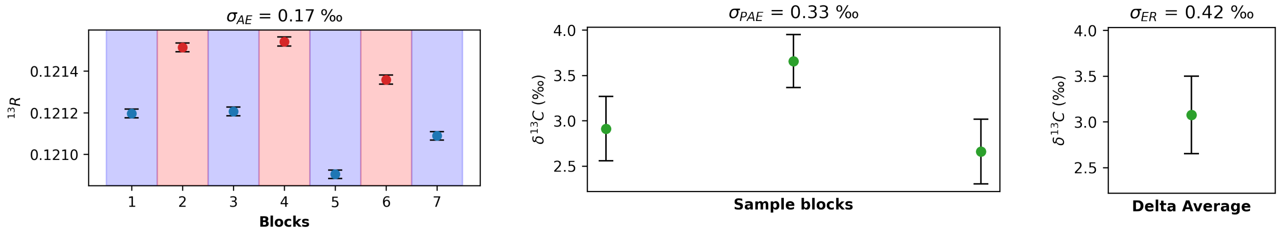

THN Mix + NH4OH 1% - Standard Concentration: 50 uM; Sample Concentration: 50

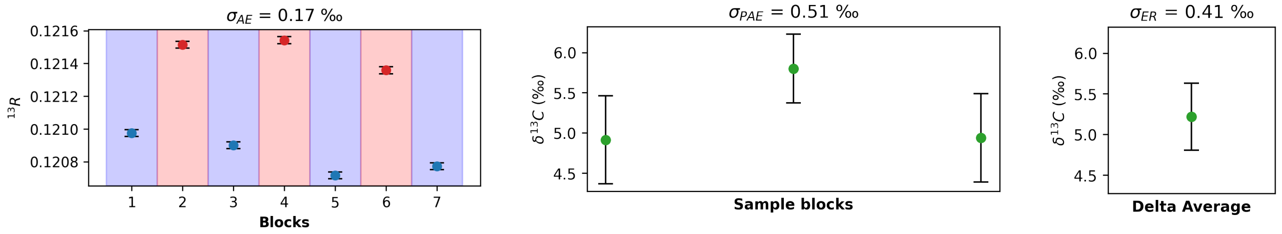

THN Mix (without ATC) + NH4OH 1%

THN Mix (without ATC) + NH4OH 1% - Standard Concentration: 0.1 uM; Sample Concentration: 0.1

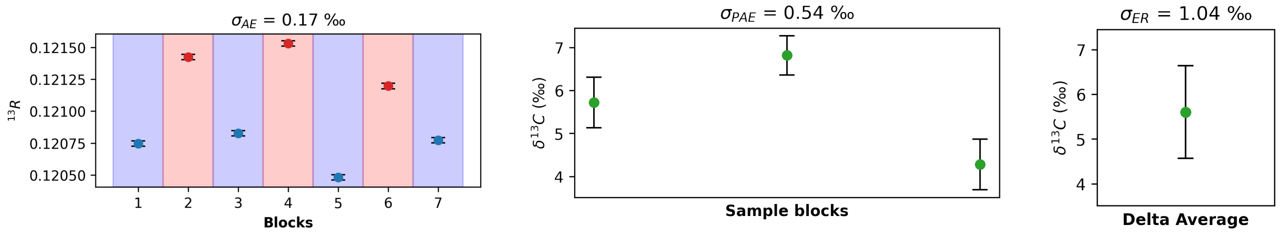

THN Mix (without ATC) + NH4OH 1% - Standard Concentration: 1 uM; Sample Concentration: 0.1

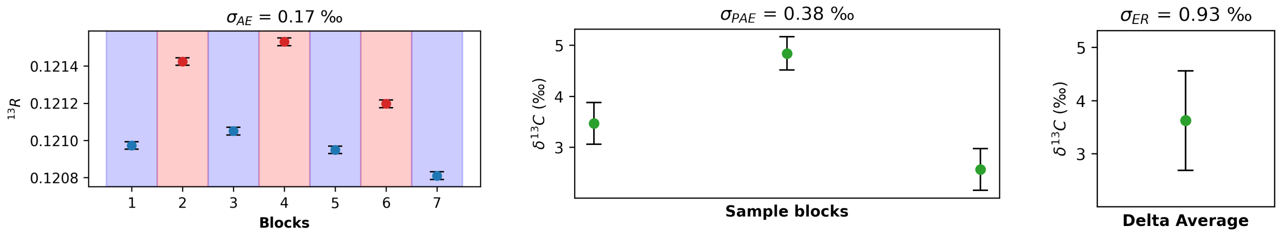

THN Mix (without ATC) + NH4OH 1% - Standard Concentration: 5 uM; Sample Concentration: 0.1

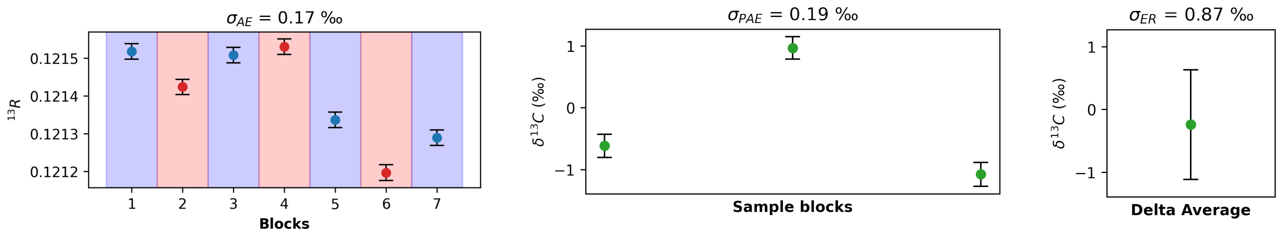

THN Mix (without ATC) + NH4OH 1% - Standard Concentration: 25 uM; Sample Concentration: 0.1

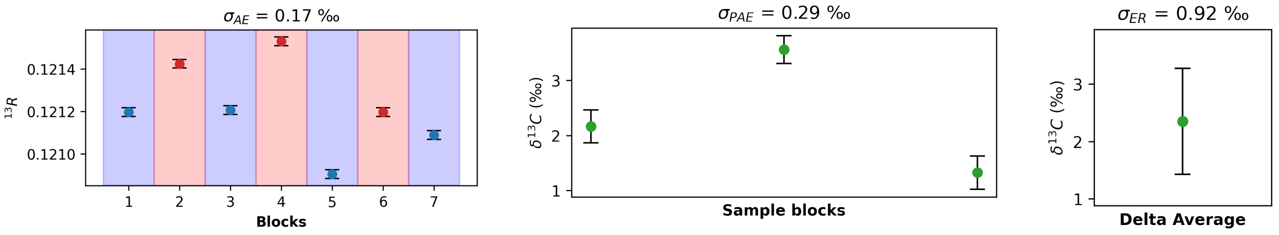

THN Mix (without ATC) + NH4OH 1% - Standard Concentration: 50 uM; Sample Concentration: 0.1

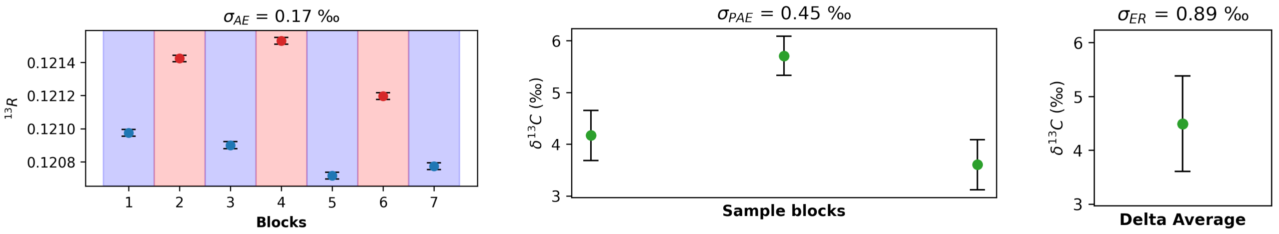

THN Mix (without ATC) + NH4OH 1% - Standard Concentration: 0.1 uM; Sample Concentration: 1

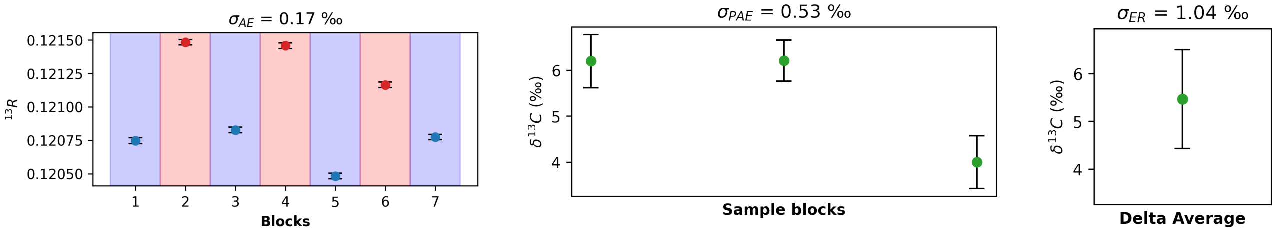

THN Mix (without ATC) + NH4OH 1% - Standard Concentration: 1 uM; Sample Concentration: 1

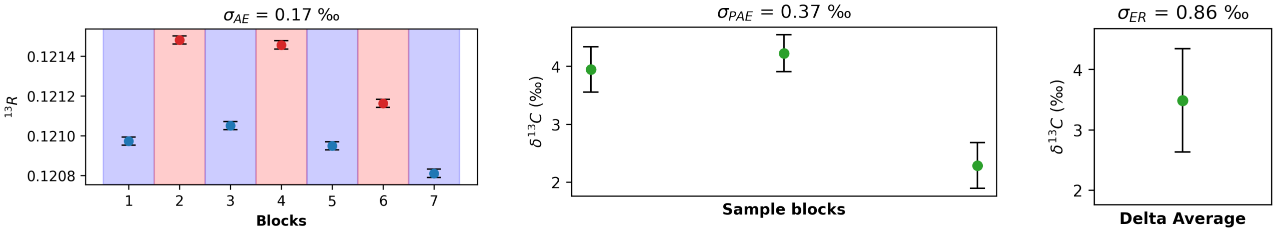

THN Mix (without ATC) + NH4OH 1% - Standard Concentration: 5 uM; Sample Concentration: 1

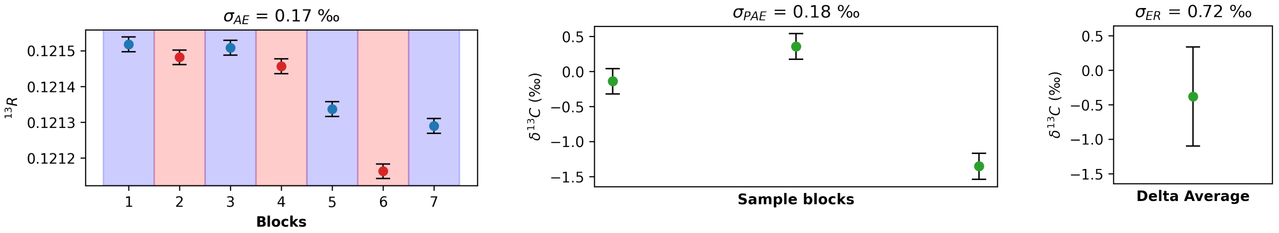

THN Mix (without ATC) + NH4OH 1% - Standard Concentration: 25 uM; Sample Concentration: 1

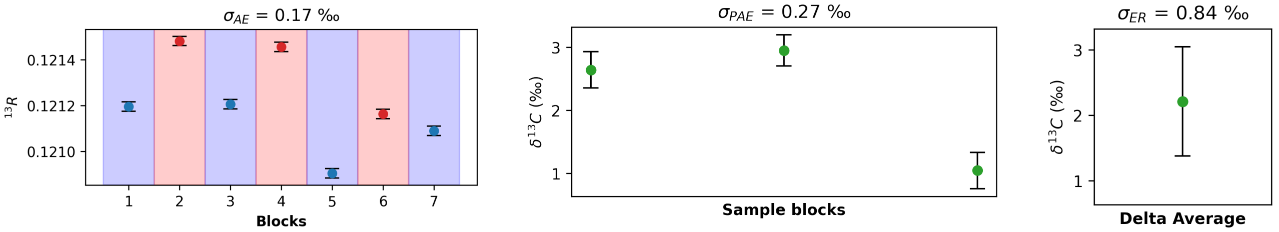

THN Mix (without ATC) + NH4OH 1% - Standard Concentration: 50 uM; Sample Concentration: 1

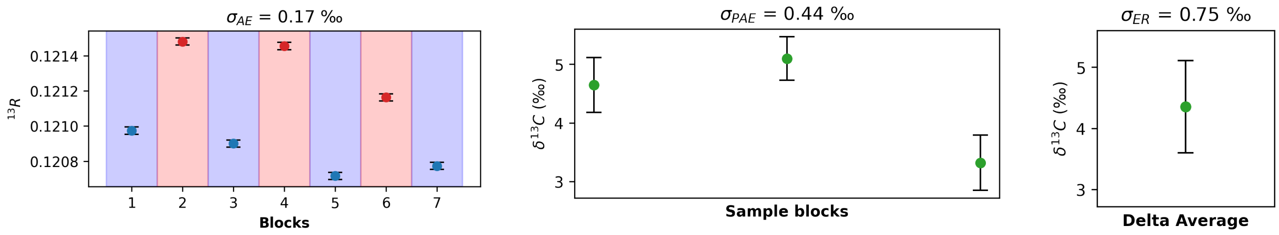

THN Mix (without ATC) + NH4OH 1% - Standard Concentration: 0.1 uM; Sample Concentration: 5

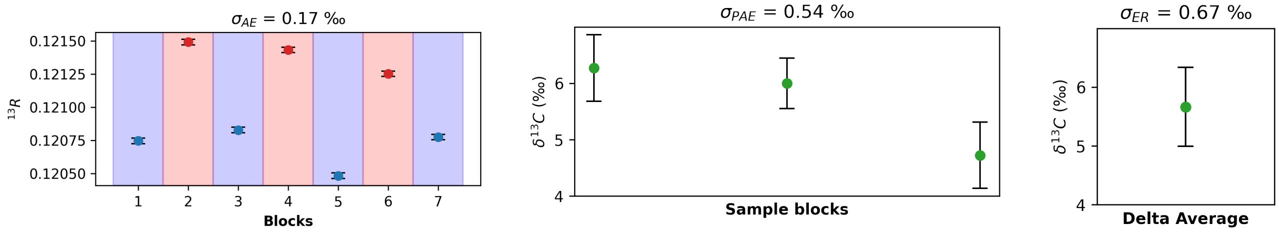

THN Mix (without ATC) + NH4OH 1% - Standard Concentration: 1 uM; Sample Concentration: 5

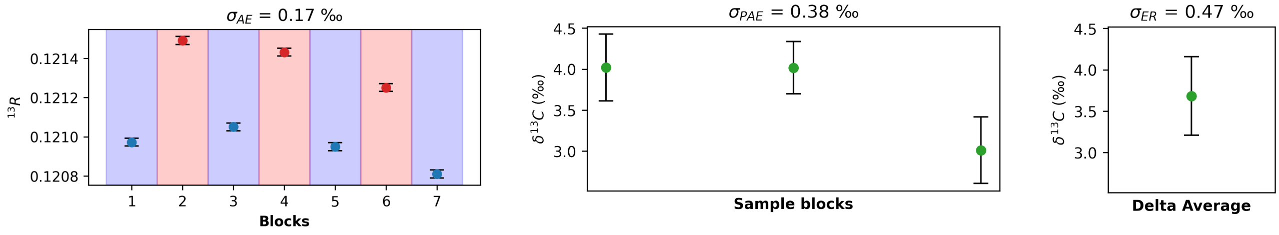

THN Mix (without ATC) + NH4OH 1% - Standard Concentration: 5 uM; Sample Concentration: 5

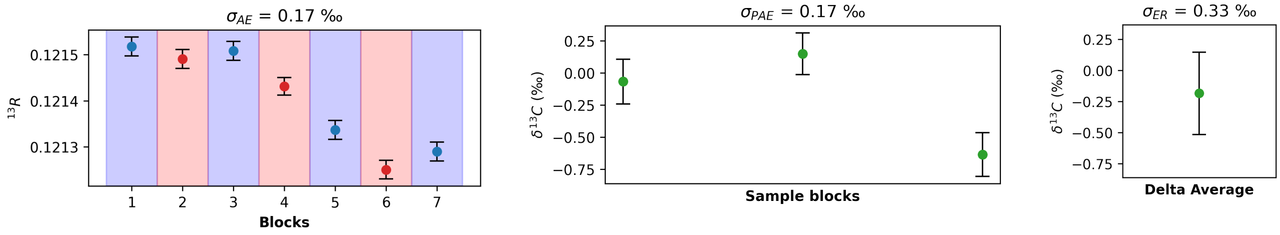

THN Mix (without ATC) + NH4OH 1% - Standard Concentration: 25 uM; Sample Concentration: 5

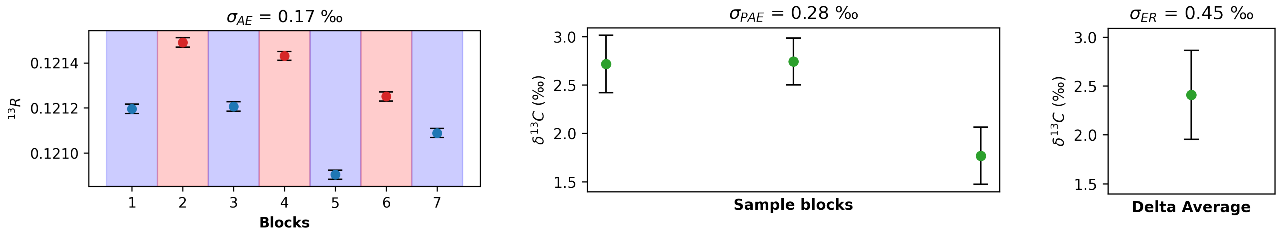

THN Mix (without ATC) + NH4OH 1% - Standard Concentration: 50 uM; Sample Concentration: 5

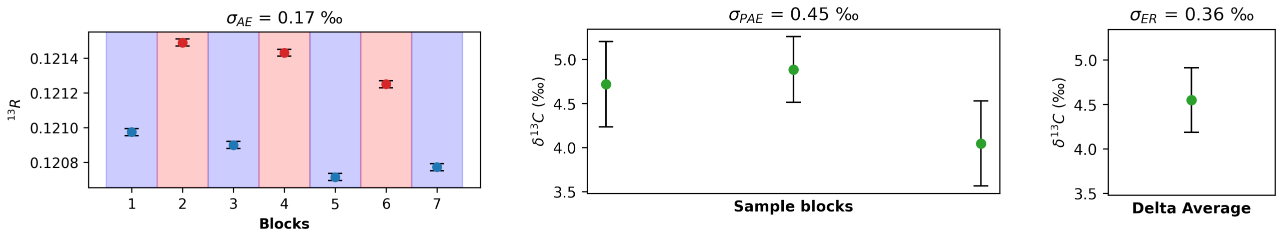

THN Mix (without ATC) + NH4OH 1% - Standard Concentration: 0.1 uM; Sample Concentration: 25

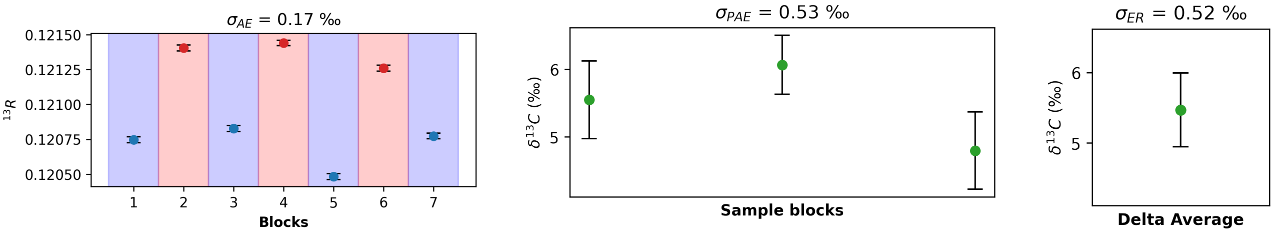

THN Mix (without ATC) + NH4OH 1% - Standard Concentration: 1 uM; Sample Concentration: 25

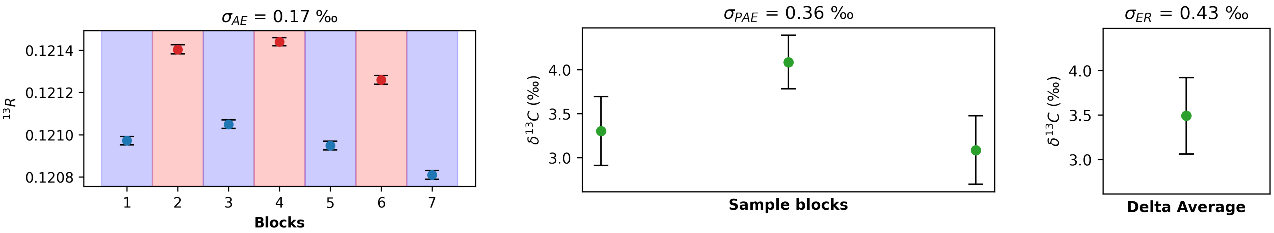

THN Mix (without ATC) + NH4OH 1% - Standard Concentration: 5 uM; Sample Concentration: 25

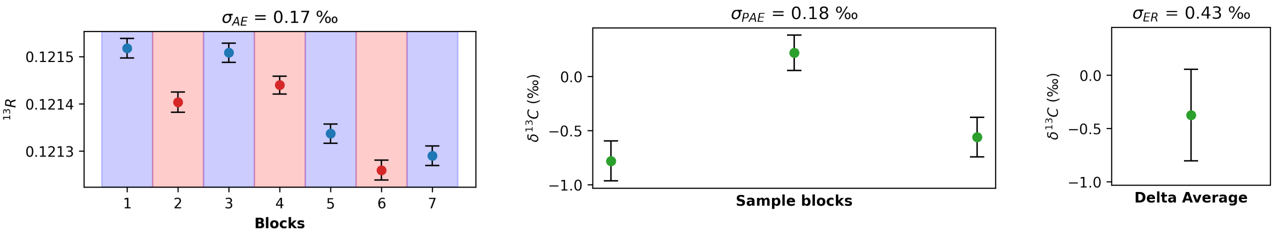

THN Mix (without ATC) + NH4OH 1% - Standard Concentration: 25 uM; Sample Concentration: 25

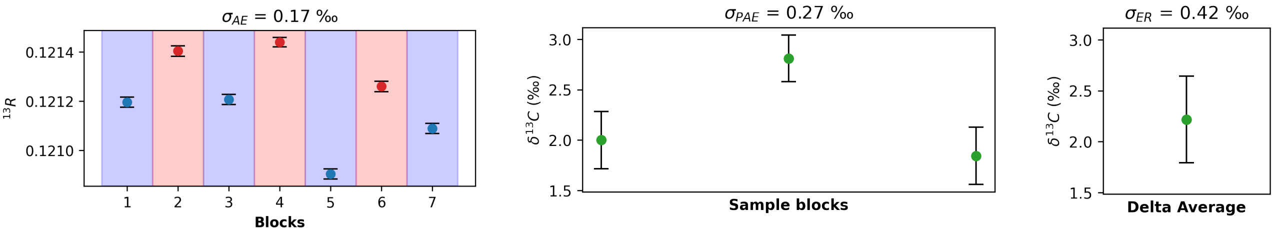

THN Mix (without ATC) + NH4OH 1% - Standard Concentration: 50 uM; Sample Concentration: 25

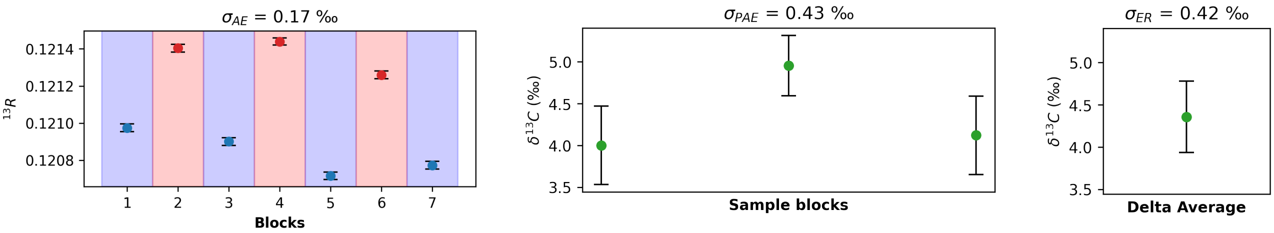

THN Mix (without ATC) + NH4OH 1% - Standard Concentration: 0.1 uM; Sample Concentration: 50

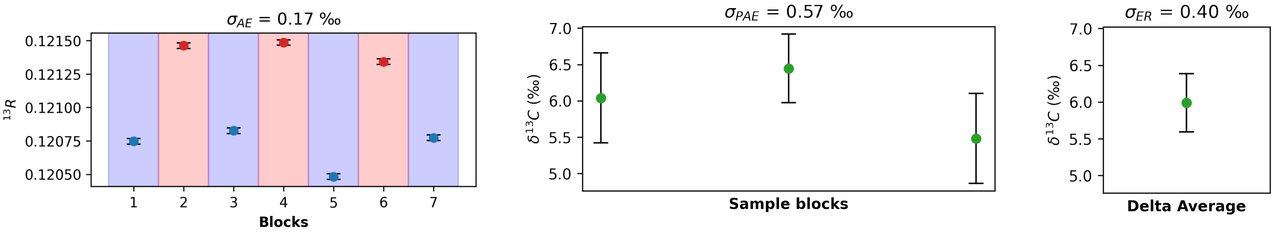

THN Mix (without ATC) + NH4OH 1% - Standard Concentration: 1 uM; Sample Concentration: 50

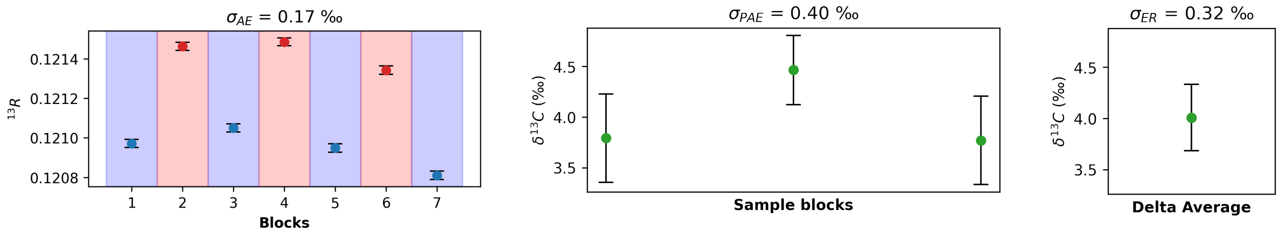

THN Mix (without ATC) + NH4OH 1% - Standard Concentration: 5 uM; Sample Concentration: 50

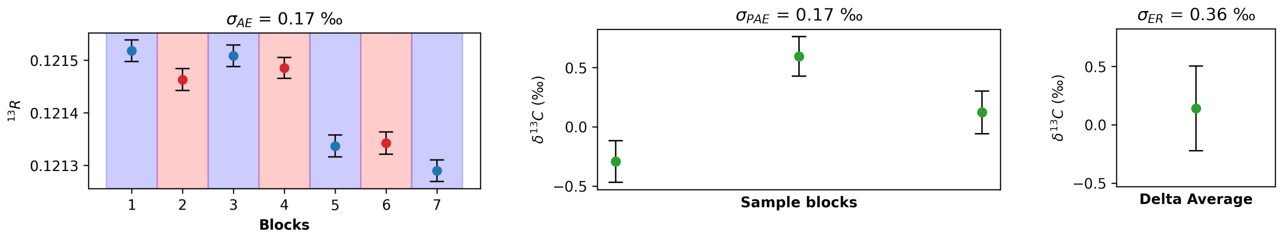

THN Mix (without ATC) + NH4OH 1% - Standard Concentration: 25 uM; Sample Concentration: 50

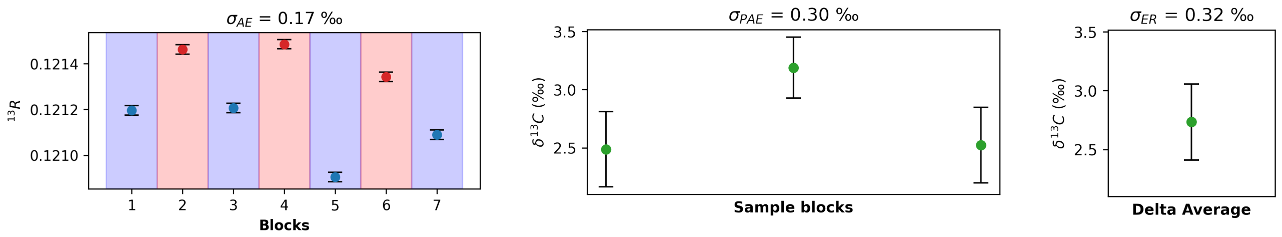

THN Mix (without ATC) + NH4OH 1% - Standard Concentration: 50 uM; Sample Concentration: 50

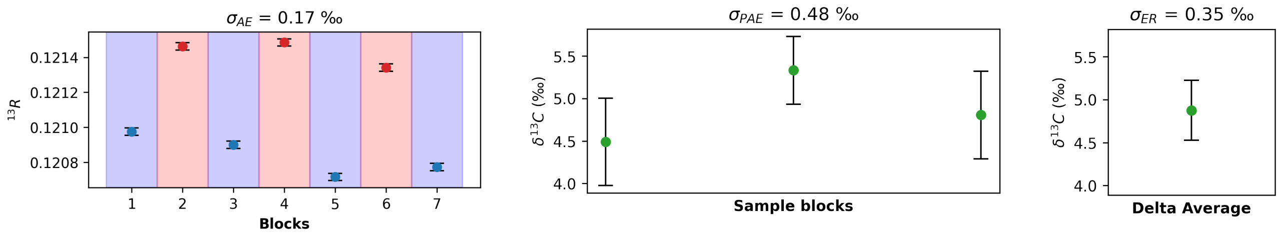

Supplement: Supplementary file 2 [file ac5c07111_si_002.pdf]
